# Cytotoxic Rocaglate Derivatives from Leaves of *Aglaia perviridis*

**DOI:** 10.1038/srep20045

**Published:** 2016-01-28

**Authors:** Fa-Liang An, Xiao-Bing Wang, Hui Wang, Zhong-Rui Li, Ming-Hua Yang, Jun Luo, Ling-Yi Kong

**Affiliations:** 1 State Key Laboratory of Natural Medicines, Department of Natural Medicinal Chemistry, China Pharmaceutical University, 24 Tong Jia Xiang, Nanjing 210009, People’s Republic of China

## Abstract

Rocaglates are a series of structurally complex secondary metabolites with considerable cytotoxicity that have been isolated from plants of the *Aglaia* genus (Meliaceae). A new rocaglate (aglapervirisin A, **1**) and its eight new biosynthetic precursors of rocaglate (aglapervirisins B-J, **2**–**9**) together with five known compounds, were isolated from the leaves of *Aglaia perviridis*. Their structures were elucidated based on a joint effort of spectroscopic methods [IR, UV, MS, ECD, 1D- and 2D-NMR, HRESIMS], chemical conversion and single-crystal X-ray diffraction. Among these isolates, three (**1, 10–11)** were silvestrols, a rare subtype rocaglates, exhibiting notable cytotoxicity against four human tumor cell lines, with IC_50_ values between 8.0 and 15.0 nM. Aglapervirisin A (**1**) induces cell cycle arrest at the G2/M-phase boundary at concentration 10 nM accompanied by reductions in the expression levels of Cdc2 and Cdc25C in HepG2 cells after 72h co-incubation, and further induces the apoptosis of HepG2 cells at concentrations over 160 nM.

Rocaglate, a class of structurally complex secondary metabolites from plants of the *Aglaia* genus (Meliaceae), have attracted great attentions for their considerable cytotoxicity^1^,^2^,^3^,^4^,^5^. Rocaglates could block cell cycle progression^6^,^7^,^8^,^9^,^10^,^11^,^12^,^13^ from G2 to M and exhibited promising activity in human tumor cell lines and xenograft models. Their cytostatic effects are comparable to those of established anticancer drugs such as vinblastine sulphate, actinomycin D, and hydroxycamptothecine^14^,^15^. Rocaglates possess cyclopenta[*b*]benzofuran skeleton, which originates from the [3 + 2] cyclization products of flavonol and diamide (cyclopenta[*bc*]benzofurans). Silvestrols, featuring an unprecedented dioxanyloxy unit attached to phenyl ring A of the cyclopenta[*b*]benzofuran skeleton, is a rare subtype of rocaglate, mostly of marked cytotoxicity^8^,^11^,^13^. The structure complexity and the potent activities of silvestrols have attracted significant interest in their biosynthesis and total synthesis^16^,^17^,^18^,^19^.

In the plant kingdom, silvestrol analogues are characteristically and exclusively present in *Aglaia* species, and only four silvestrols have been reported^4^ as yet. Certain *Aglaia* species have been used as traditional medicines for treating fever, cough, diarrhoea, and contused wounds^4^,^5^. In continuation of the discovery of novel and bioactive natural products from plants of the Meliaceae family^20^,^21^,^22^, the species *A. perviridis*, a wild shrub indigenous to Yunnan Province of China^20^,^21^,^22^, was investigated to find cytotoxic rocaglates, particularly silvestrols. As a result, three silvestrol analogues including a new one (aglapervirisin A, **1**), eight new biosynthetic precursors of rocaglates (cyclopenta[*bc*]benzopyrans, **2**–**9**), and three known precursors (**12**–**14**) were isolated and purified from the leaves of *A. perviridis* (Fig. 1). Their structures were mainly elucidated through comprehensive analysis using spectroscopic methods, including IR, UV, MS, HRESIMS, 1D-NMR and 2D-NMR. The absolute configuration of **1** was determined by ECD analysis and chemical conversion, and that of **2** was established by single-crystal X-ray diffraction using Cu K*α* radiation. These isolates (except for **6** and **12**) were evaluated for their cytotoxicity against four human cancer cell lines: three silvestrol analogues (**1**, **10** and **11**) showed potent activity with IC_50_ values between 8.0 and 15.0 nM. Of them, **1** induced cell cycle arrest by reducing the Cdc2 and Cdc25C expression levels in a dose-dependent manner and induced the apoptosis of these cells at concentrations over 160 nM. Herein, we report the separation and structural elucidation of these isolated rocaglate derivatives, as well as the bioassay results.

## Results and Discussion

**Figure 2:**
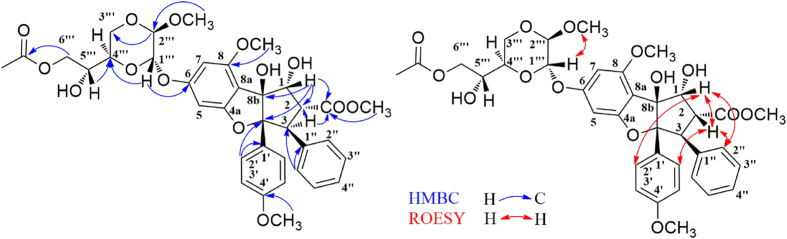
Selected key HMBC and ROESY correlations observed for 1.

Aglapervirisin A (**1**), 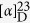 −82.1 (*c*, 0.11, MeOH), was obtained as colourless powder with the molecular formula C_28_H_27_NO_8_, as deduced from the [M + Na]^+^ ion peak in HRESIMS data (C_36_H_40_O_14_Na, *m/z* 719.2313). The ^1^H NMR spectrum of **1** displayed resonances for the four aromatic protons of a 1,4-disubstituted benzene, five aromatic protons of a monosubstituted benzene, two aromatic protons of a 1,2,3,5-tetrasubstituted benzene, and four methoxy groups. Its 1D-NMR (Table 1) data, particularly the three characteristic proton signals at *δ*_H_ 5.11(1H, d, *J* = 7.0 Hz), *δ*_H_ 3.91 (1H, dd, *J* = 14.5, 7.0 Hz), and *δ*_H_ 4.32(1H, d, *J* = 14 Hz), featured a cyclopenta[*b*]benzofuran derivative nature of **1**^23^. A series of proton signals at *δ*_H_ 3.5 to 5.5, carbon signals at *δ*_C_ 60.0 to 70.0, and two characteristic acetal carbons at *δ*_C_ 93.7 and 95.3, suggested the presence of a dioxanyloxy unit in the structure of **1**. The above-described analysis indicated that compound **1** was a silvestrol (**10**) analogue. Compared with **10**, the presence of an acetyl group in **1** was evidenced by characteristic NMR signals (*δ*_H_ 1.89, *δ*_C_ 51.9, 171.7) and 42 mass unit more than **10**. The key HMBC correlation (Fig. 2) from H-6′′′ (*δ*_H_ 4.08 and 3.99) to the acetyl group (*δ*_C_ 20.5) indicated that the acetyl group was located at 6′′′-OH. The key ROESY correlations (Fig. 2) of H-1/H-2, H-2′, 6′ and H-2/H-2′, 6′, H-2″, 6″, also observed for **10**^23^, indicate that H-1, H-2, 1,4-disubstituted benzene and monosubstituted benzene were co-facial. Thus, the planar structure and relative configuration of **1** was determined. The similar ECD spectra of **1** and silvestrol (**10**) indicated that the absolute stereochemistry of the basic skeleton of **1** was the same as that of silvestrol. However, the absolute configuration of C-5′′′ in **1** was difficult to determine based on the ECD comparison. To completely determine the absolute configuration of **1**, the acetyl derivative of **1** (**1a**), silvestrol (**10a**) and episilvestrol (**11a**) (see Figure S16) were prepared by acylation. The identical HRESIMS (**1a**, *m/z* 756.2857, [M + NH_4_]^+^, **10a**, *m/z,* 756.2859, [M + NH_4_]^+^), retention times in HPLC (**1a***, t*_R_ = 8.1 min, **10a**, *t*_R_ = 8.1 min, 70% MeOH- H_2_O), and the NMR data (see in SI), as well as the similar optical values obtained for **1a** and **10a** and the different retention times in HPLC (**1a***, t*_R_ = 8.1 min, **11a**, *t*_R_ = 9.3 min, 70% MeOH- H_2_O) and the NMR data (see in SI) obtained for **1a** and **11a**, indicate that the absolute configuration of **1** was consistent with that of silvestrol (**10**)^23^. The C-5′′′ in **1** was adopted as *R* based on the *R* configuration of C*-*5′′′ in compound **10**^23^. Thus, the structure of **1** was determined, and named as aglapervirisin A.

Aglapervirisin B (**2**) was obtained as colourless crystal (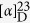 −28.3) and gave a sodiated molecular ion [M + Na]^+^ at *m/z* 675.2672 (calcd 675.2677) in the HRESIMS corresponding to a molecular formula of C_38_H_40_N_2_O_8_, which requires 20 indices of hydrogen deficiency. In the ^1^H NMR spectrum, the 16 aromatic hydrogen signals in the low-field region (*δ*_H_ 6.09–7.85) presented four benzene rings, including two monosubstituted benzene rings, a 1,4-disubstituted benzene ring, and a 1,2,3,5-tetrasubstituted benzene ring. Two amide protons at *δ*_H_ 6.47 (NH-17) and 5.26 (NH-12), and the remaining methylenes suggested the presence of a 1,4-butanediamide chain. These characteristic proton signals revealed that **2** was a cyclopenta[*bc*]benzopyran derivative^24^,^25^,^26^,^27^.

**Figure 3:**
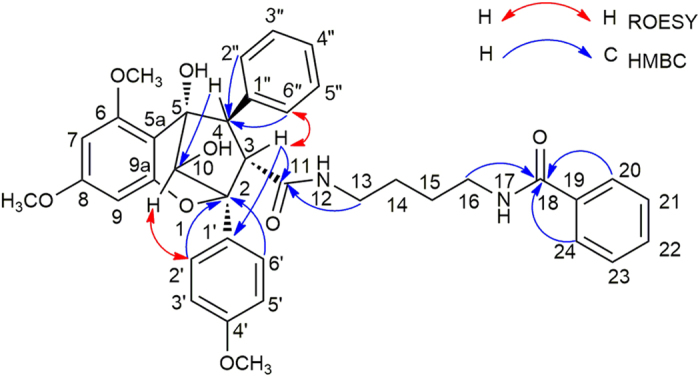
Selected key HMBC and ROESY correlations observed for 2.

**Figure 4:**
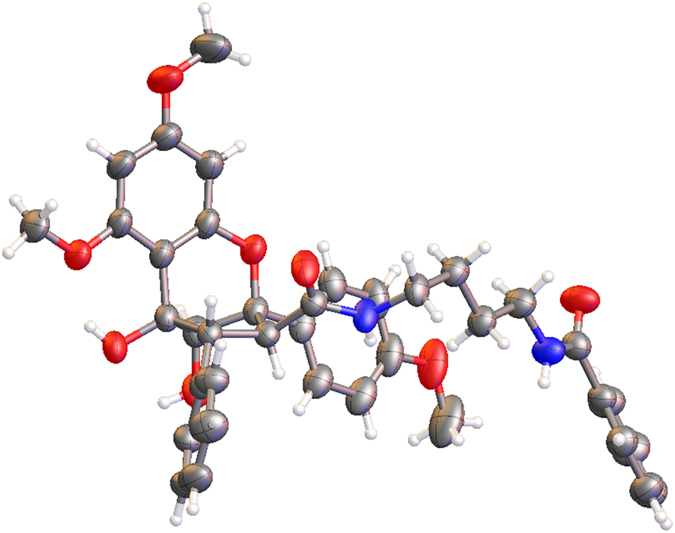
Single-crystal X-ray structure of 2.

In the HMBC spectrum (Fig. 3), a cross peak between signals at *δ*_H_ 7.76 (H-20, 24) and *δ*_C_ 167.6 (C-18) established the connection of one of the monosubstituted benzene rings to the butanediamide chain. The correlated peaks from H-3 to C-11 and from H-4 to C-1″ and C-2″, 6″ allowed the placement of the 1, 4-butanediamide moiety and the second monosubstituted benzene ring at C-3 and C-4, respectively. Thus, the planar structure of **2** was depicted in Fig. 3. The relative configuration of **2** was determined according to the coupling constants between H-3 and H-4, and the cross peaks in the ROESY spectrum (Fig. 3). The vicinal coupling constant value between H-3 and H-4 was 6.5 Hz, allowed the assignment of H-3*β* and H-4*α* configuration^24^,^25^,^26^,^27^. The ROESY correlations between H-10 and H-2″, 6″ and the lack of any correlation between H-3 and H-10 in compound **2** further confirmed the H-3*β* and H-4*α*. A single-crystal X-ray diffraction experiment (Fig. 4) using Cu K*α* radiation was performed, and the absolute configuration of five asymmetric carbons in **2** was unambiguously established as 2*R*, 3*S*, 4*R*, 5*R*, and 6*S*. The absolute configuration of such a flavonol and diamide [3 + 2] adduct containing a benzoyl-1,4-butanediamide moiety was determined for the first time via the single-crystal X-ray diffraction method.

Aglapervirisin C (**3**) was obtained as a white powder {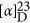 −22.1 (*c* 0.10, MeOH)}, and the molecular formula of C_38_H_40_N_2_O_8_, which is equal to that found for **2**, was determined based on the HRESIMS data (*m/z*, 675.2675, [M + Na]^+^). The NMR data (Table 2) of **3** were similar to those of **2**, which indicated that **3** was also a cyclopenta[*bc*]benzopyran derivative with a benzoyl-1,4-butanediamide moiety^28^. However, the methine signals of H-10 at *δ*_H_ 4.67 and C-10 (*δ*_C_ 76.1) in **3** showed notable differences from those of **2** (*δ*_H_ 4.19, *δ*_C_ 82.6) (Table 1), which combined with the observed obvious ROESY correlation between H-3 (*δ*_H_ 3.51) and H-10 (*δ*_H_ 4.68) in **3**, absent in **2**, revealed that **2** and **3** were epimers at C-10.

Aglapervirisin D (**4**), 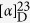 −0.3 (*c* 0.12, MeOH), was obtained as a white amorphous powder, and its molecular formula was elucidated to be C_38_H_38_N_2_O_8_ (*m/z* 673.2516, [M + Na]^+^) based on its ^13^C NMR data and HRESIMS, with one more degree of unsaturation than **2**. The similarity between the NMR data (Table 2) of **4** and **2** and the key HMBC correlations between H-3/ C-2″, 6″ and H-4/C-11 suggest that **4** was also a cyclopenta[*bc*]benzopyran^4^,^27^,^28^ derivative, similar to **2**. Compared with **2**, the presence of an additional carbonyl group (*δ*_C_ 209.5) and the absence of the characteristic signal of C-10 (*δ*_H_ 4.19, *δ*_C_ 82.6) indicated that **4** was an oxidation derivate at C-10 of **2**. This deduction was confirmed by the HMBC correlations between H-4 (*δ*_H_ 4.66) and C-10. The coupling constant *J*_(H-3, H-4)_ (11.5 Hz)^3^,^27^ together with the important ROESY correlation between H-4 to H-2′, 6′ (*δ*_H_ 7.30), made the assignments of H-3*α* and H-4*β*. Thus, the new compound **4** was assigned as an oxidation derivate at C-10 of **2**.

Aglapervirisin E (**5**) was obtained as a white powder {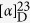 −4.4 (*c* 0.28, MeOH)} with a molecular formula of C_39_H_42_N_2_O_9_ according to the pseudomolecular ion at *m/z* 705.2784 [M + Na]^+^ (calcd C_39_H_42_N_2_O_9_Na, 705.2783). Four sets of signals for benzene-ring, including one monosubstituted, two *p*-disubstituted, and one 1,2,3,5-tetrasubstituted benzene rings, three methoxyl groups in the ^1^H NMR spectrum, and its similar HMBC correlations to those of **2** suggested that **5** was also a typical cyclopenta[*bc*]benzopyran^4^,^27^ derivative. The 1D-NMR data (Table 2) for **5** and **2** showed one more 4-hydroxybenzyl group [*δ*_H_ 7.06 (2H, d, *J* = 8.5 Hz, *δ*_C_ 129.4), *δ*_H_ 6.84 (2H, d, *J* = 8.5 Hz, *δ*_C_ 113.9)], and (*δ*_H_ 3.45, *δ*_C_ 43.1), in **5** than in **2**, which suggested that a *p*-hydroxybenzyl group moiety presented in **5** other than a benzoyl group in **2**. The obvious HMBC correlations from H-16 (*δ*_H_ 2.95) and H-19 (*δ*_H_ 3.45) to the amide carbonyl carbon (172.0, C-18) placed the *p*-hydroxybenzyl group at N-17. Thus, the planar structure of **5** was determined as depicted. In the ^1^H NMR spectrum of **5** (Table 3), an extremely upfield shifted methoxyl signal was observed at *δ*_H_ 3.09. The coupling constant value (8.5 Hz) of *J*_(H-3,H-4)_ was also differ greatly from that of **2** (6.5 Hz). These data characterized H-3*α* and H-4*β* in **5**^3^,^24^,^28^, respectively, in opposite to those in **2**. A key ROESY correlation between H-10 (*δ*_H_ 4.89) and H-3 (*δ*_H_ 3.86) confirmed that **5** had opposite relative configurations at position C-3 and C-4 compared with **2**, which also established an *endo* relationship between H-3 and H-10^28^. Thus, the structure of **5** was established as shown.

The molecular formula of aglapervirisin F (**6**) {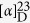
−5.4 (*c* 0.28, MeOH)} was determined to be C_38_H_40_N_2_O_9_ by HRESIMS (*m/z* 691.2625, [M + Na]^+^, calcd 691.2626), with one CH_2_ unit less than **5**. Its ^1^H and ^13^C NMR data, particularly the characteristic methoxyl signal at *δ*_H_ 3.10 and the vicinal coupling constant value (8.5 Hz) of H-4/H-3, resemble greatly with those of **5**, which indicated that the basic skeleton of **6** was the same as that of **5**. The absence of a characteristic methine (*δ*_H_ 3.45, *δ*_C_ 43.1, C-19) indicated that the *p*-hydroxyphenylacetyl group at N-17 in **5** was replaced by a 4-hydroxybenzoyl group moiety in **6**, as supported also by the observed HMBC correlations from H-16 (*δ*_H_ 3.17) and H-20/24 (*δ*_H_ 7.67) to C-18 (*δ*_C_ 170.0). The ROESY correlations of H-10/H-3 indicated that the relative configuration of **6** was also the same as that of **5**. Finally, the structure of **6** was assigned as shown.

Aglapervirisin G (**7**) was obtained as an optically active, white amorphous powder (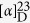 −22.1). The molecular formula of **7** was determined to be C_38_H_40_N_2_O_8_ (*m/z* 675.2675, [M + Na]^+^, calcd 675.2677), the same as **2**. The ^1^H NMR resonances of **7** resembled those of **2**, including five benzene ring signals, a characteristic singlet for H-10, and two apparent doublets (H-3, H-4), indicated that **7** was an isomer of **2**. The key HMBC correlations of H-3 (*δ*_H_ 4.25) /C-2″6″ (*δ*_C_ 129.4), H-2″6″ (*δ*_H_ 6.90) /C-3 (*δ*_C_ 53.8) and H-4 (*δ*_H_ 3.09) /C-11 (*δ*_C_ 169.3) in **7**, were not consistent with those of **2**, which indicated that the substituents at C-3 and C-4 were mutually exchanged in **7**^3^,^24^,^27^. Thus, the planar structure of **7** was determined. The relative configurations of H-3*α* and H-4*β* were determined based on the vicinal coupling constant (*J* = 9.0 Hz)^28^ and the ROESY correlations between H-3/H-2′, 6′, H-3/H-2″, 6″, H-4/H-2″, 6″, and H-4/NH-12^24^,^28^. The key cross peak between H-10 and H-4 indicated an *endo* relationship between H-10 and H-4^28^. Thus, the structure of **7** was proposed as depicted.

**Figure 5:**
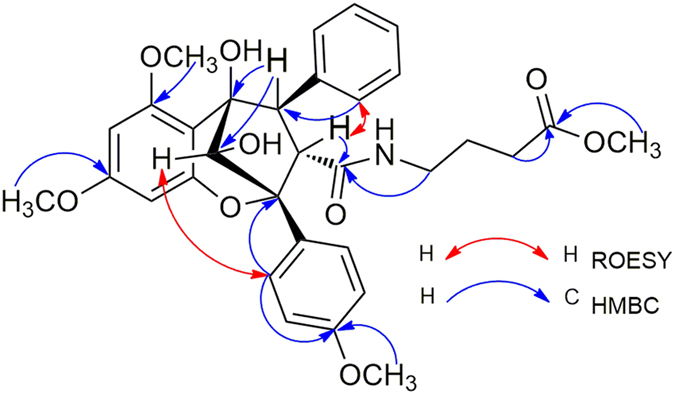
Selected key HMBC and ROESY correlations observed for 8.

**Figure 6:**
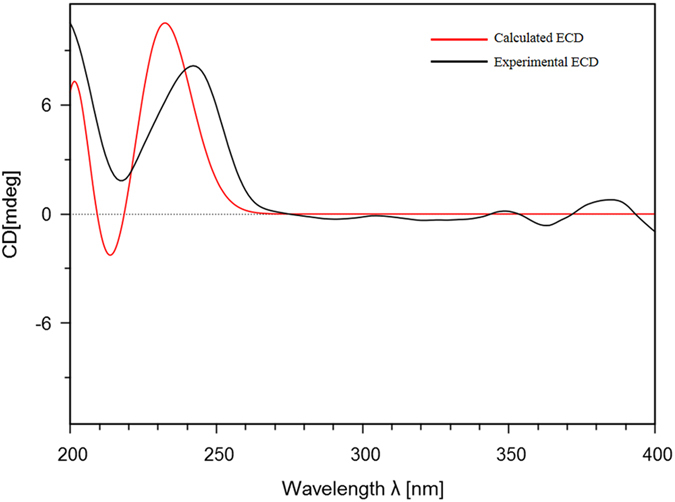
Theoretically calculated and experimentally determined ECD of 8.

Aglapervirisin H (**8**) was obtained as a colourless powder, 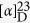 + 96.9 (*c* 0.10, MeOH), exhibited a sodicated molecular ion at *m/z* 600.2202 [M + Na]^+^ (calcd for C_32_H_35_NO_9_Na, 600.2204) in the HRESIMS. The eleven characteristic aromatic protons at *δ*_H_ (7.71−5.78) and 18 carbon signals observed in the ^1^H and ^13^C NMR data of **8**, indicated that this compound had four aromatic protons and six aromatic carbons less than **5**. The presence of a characteristic methoxyl at *δ*_H_ 3.62 and obvious HMBC correlations from H-15 (*δ*_H_ 1.94) and the methoxyl signal (*δ*_H_ 3.62) to a carbonyl carbon at *δ*_C_ 173.4 indicated that the benzoyl-1,4-butanediamide moiety in **2** was replaced by a 4-aminobutanoate methyl ester moiety in **8**^3^. The key HMBC correlations (Fig. 5) of the resonances of H-3 (*δ*_H_ 3.88) with C-11 (*δ*_C_ 173.8) and H-4 (*δ*_H_ 4.10) with C-2″, 6″ (*δ*_C_ 128.7) indicated that the basic connection of the core planar structure in **8** was the same as that of **5**. The characteristic deshielded shift of the 6-OMe (*δ*_H_ 3.08) signal and the coupling constant *J*_(H-3,H-4)_ (8.5 Hz) indicated that the relative configurations of **8** at C-3 and C-4 were the same as those of **5**^3^,^24^,^28^. The absence of a cross peak between H-10 (*δ*_H_ 4.89) and H-3 (*δ*_H_ 3.88) indicated an *exo* relationship between H-10 and H-3^28^. The absolute configuration of **8** was assigned as 2*R*, 3*R*, 4*S*, 5*R*, and 6*S* based on the calculated ECD (Fig. 6). Thus, **8** was a new cyclopenta[*bc*]benzopyran-type^4^,^27^ derivative, depicted in Fig. 5.

Aglapervirisin I (**9**) was obtained as a colourless powder {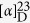 + 85.4 (*c,* 0.15, MeOH)} with a molecular formula of C_31_H_35_NO_8_, as deduced from its HRESIMS (*m/z* 572.2253 [M + Na]^+^, calcd 572.2255). The ^1^H and ^13^C NMR data of **9** were similar to those of **8**. The obvious differences between them were the presence of two multiplet protons at *δ*_H_ 3.40 and the absence of a carbonyl carbon and a methoxy group in **9**. The above information indicated that the methyl side chain was reduced to a hydroxymethyl group, as confirmed by the key HMBC correlations between H-15 (*δ*_H_ 1.10) and C-16 (*δ*_C_ 62.5). The coupling constant of *J*_(H-3,H-4)_ (8.5 Hz) and characteristic deshielded shift of the 6-OMe (*δ*_H_ 3.07) signal indicated that the planar structure and relative configuration of **9** were consistent with those of **8**. Furthermore, the absolute configuration of **9** was elucidated as 2*R*, 3*R*, 4*S*, 5*R*, and 6*S* based on its ECD data, which was similar to that of **8**. Thus, **9** was elucidated to be a new cyclopenta[*bc*]benzopyran derivative, and was named as aglapervirisin I.

Five known compounds were identified as silvestrol (**10**) (*m/z* 655.2387, [M + H]^+^), episilvestrol (**11**) (*m/z* 655.2380, [M + H]^+^), foveoglin A (**12**) (*m/z* 653.2854, [M + H]^+^), foveoglin B (**13**) (*m/z* 653.2856, [M + H]^+^), and cyclofoveoglin (**14**) (*m/z* 651.2701, [M + H]^+^) based on comparisons of their ^1^H NMR, ^13^C NMR, and ESIMS data with reported values in the published literatures^23^,^28^.

### Aglapervirisin A (1) inhibits proliferation of HepG2 cells by causing G2/M-phase arrest in HepG2 cells

The cytotoxicity of all of the isolates (except for **6** and **12**) against four human tumour cell lines was tested. Silvestrol analogues (**1**, **10**, and **11**) exhibited significant cytotoxicity towards the tested tumor cell lines and that the cyclopenta[*bc*]benzopyrans (**2**–**5**, **7**–**9**, **13**, **14**) were inactive or only of moderate cytotoxicity (Table 4).

**Figure 7:**
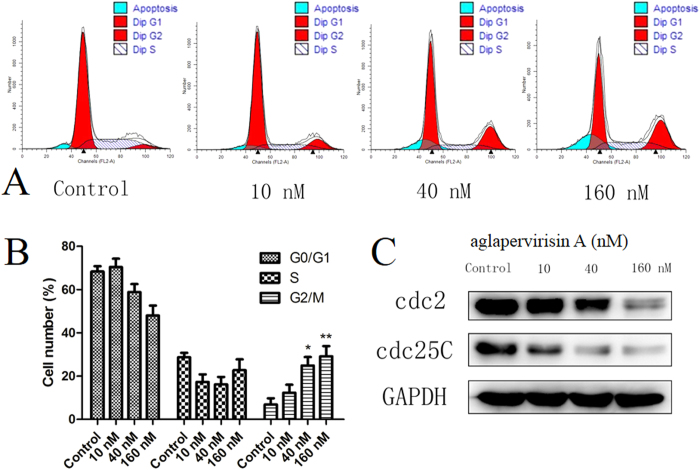
Compound 1 induces cell cycle arrest and apoptotic cell death in HepG2 cells. (**A**) Compound **1** induces cell cycle arrest at the G2/M boundary. The cells were treated with vehicle or with **1** at 0 *μ*M, 6.25 *μ*M, 12.5 *μ*M, or 25 *μ*M for 48 h, and the cell cycle distribution was assessed by flow cytometry; (**B**) Percentages of cells in different phases of the cell cycle; **(C)** The levels of Cdc2 and Cdc25C were measured by western blotting using GAPDH as the loading control. Data are presented as the means ± SD of three experiments. *P < 0.05, **P < 0.01 compared to the control group.

Cell cycle arrest is one of the important factors that influence the proliferation of tumour cells^29^. Thus, the regulation of the cell cycle of HepG2 cells by aglapervirisin A (**1**), a new silvestrol analogue possessing significant cytotoxicity, was investigated through flow cytometry analysis. Treatment of HepG2 cells with **1** at concentrations of 40 or 160 nM for 48 h increased percentage of HepG2 cells arrested at the G2/M boundary from 10% to 25% or 35%, respectively. These results indicated that **1** can induce the arrest of HepG2 cells at the G2/M boundary in a dose-dependent manner, similarly to hydroxycamptothecin^14^,^15^, as shown in Fig. 7. The western blotting results indicated that **1** down-regulates the expression of Cdc2 and Cdc25C, which may be responsible for the induction of G2/M arrest.

### Aglapervirisin A (1) causes HepG2 cell death by apoptosis

**Figure 8:**
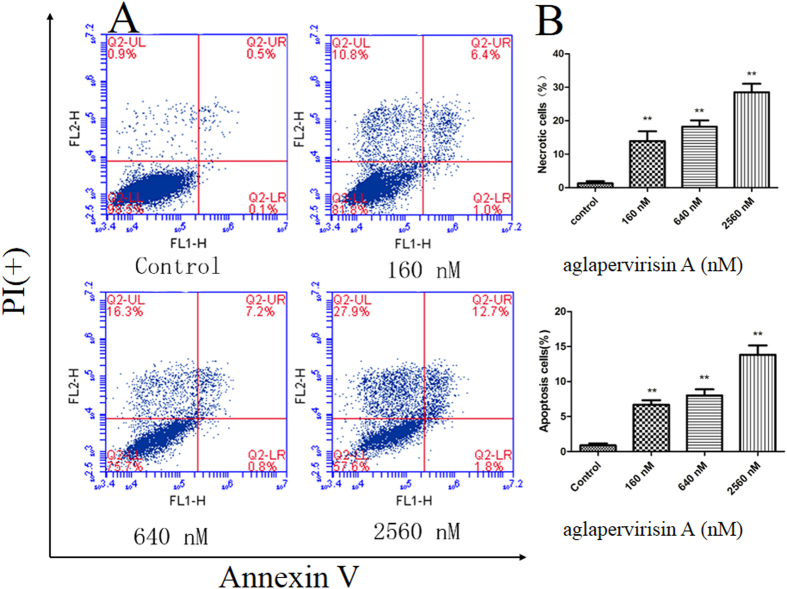
Analysis of apoptosis in 1-treated HepG2 cells. (**A**) The cells were treated with **1** at 160 nM, 640 nM or 2560 nM for 72 h. (**B**) The numbers of apoptotic cell were calculated by flow cytometry. Data are presented as the means ± SD of three experiments. *P < 0.05, **P < 0.01 compared to the control group.

The induction of apoptosis is also a mechanism through which antitumour agents exert their therapeutic effects^30^,^31^. Thus, the induction of apoptosis by **1** was also examined in this study (Fig. 7). The assay showed that incubation with **1** for 72 h induced the apoptosis of HepG2 cells in a dose-dependent manner. The percentages of apoptotic cells were 6.2% and 14.5% after treatment with **1** at concentrations of 160 and 2560 nM, respectively, as compared with 0.6% in the negative control group. Thus, these results demonstrated that **1** induced HepG2 cell death by apoptosis (Fig. 8).

## Conclusion

In summary, this paper describes the isolation and structural elucidation of a rare silvestrol analogue (aglapervirisin A, **1**), eight new biosynthetic precursors of rocaglate (cyclopenta[*bc*]benzopyrans, aglapervirisins A-H, **2**–**9**), and five known analogues isolated from the leaves of *A. perviridis*. The absolute configurations of **1**, **2**, and **8** were confirmed through chemical conversion, single-crystal X-ray diffraction, and quantum chemical calculations of ECD, respectively. The new silvestrol analogue **1** showed significant cytotoxicity at the nanomolar level against several cancer cell lines and a further mechanistic study indicated that this cytotoxicity was associated with the induction of G2/M phase arrest through reductions in the expression levels of Cdc2 and Cdc25C and the induction of apoptosis. Taken together, these findings indicate that the isolated compounds are potential natural prodrugs with anticancer activity.

## Methods

### General Experimental Procedures

The optical rotations were measured on a JASCO P-1020 polarimeter at room temperature. The melting points were measured using an X-4 digital display micromelting apparatus and are uncorrected. The IR spectra were recorded on a Bruker Tensor 27 spectrometer using KBr pellets. The 1D- and 2D-NMR spectra were measured on a Bruker AVIII-500 NMR instrument (^1^H: 500 MHz, ^13^C: 125 MHz) using TMS as the internal standards. HRESIMS was performed on an Agilent 6529B Q-TOF mass instrument using electrospray ionization. All of the solvents used were of analytical grade (Jiangsu Hanbang Science and Technology. Co., Ltd.). Silica gel (200–300 mesh, Qingdao Haiyang Chemical Co., Ltd, China), Sephadex LH-20 (Pharmacia, Sweden), MCI (Mitsubishi, Japan) and RP-C18 silica (40–63 *μ*m, Fuji, Japan) were used for the column chromatography. Preparative HPLC was conducted using an Agilent 1260 Series instrument with a Shim-Pak RP-C18 column (20 × 200 mm), at a flow rate of 10.0 mL/min, and detection by a binary channel UV detector. The fractions obtained from CC were monitored by TLC with precoated silica gel GF_254_ (Qingdao Haiyang Chemical Co., Ltd, China) plates.

### Plant Material

Air-dried leaves of *A. perviridis* Hiern were collected from Xishuangbanna, Yunnan Province, People’s Republic of China, in June 2013, and were identified by Professor Shun-Cheng Zhang, Xishuangbanna Tropical Botanical Garden, Chinese Academy of Sciences, People’s Republic of China. A voucher specimen (No. AA201308) was deposited in the Department of Natural Medicinal Chemistry, China Pharmaceutical University.

### Extraction and Isolation

The air-dried leaves (15.0 kg) were percolated with 95% aqueous ethanol (4 × 80 L) at room temperature. After removal of the solvent under reduced pressure, the crude extract (1510.5 g) was suspended in H_2_O (1.5 L) and partitioned with petroleum ether (3 × 1 L). The petroleum ether (PE) extract (502.7 g) was suspended in PE and was reverse partitioned with 50% aqueous methanol. The objective extract (100 g) was subjected to a macroporous resin (D101) column and was eluted with water and EtOH (90:10, 50:10, 30:70, 5:95, v/v) to obtain four fractions (A-D). Fraction C (20.5 g) was separated by chromatography on a silica gel column and eluted with a gradient of PE-acetone (20:1 to 5:1, v/v) to yield five fractions (C1-C5). Fraction C1-C5 were then separated by chromatography on a Sephadex LH-20 column and purified by semi-preparative-HPLC with MeOH-H_2_O, to obtain **1** (10 mg), **2** (50 mg), **3** (30 mg), **4** (10 mg), **5** (5 mg), **6** (6 mg), **7** (3 mg), **8** (2 mg), **9** (5 mg), **10** (18 mg), **11** (17 mg), **12** (300 mg), **13** (12 mg), and **14** (9 mg).

### X-ray crystallographic data for 2

C_38_H_40_N_2_O_8_ (*M* = 652.72): orthorhombic, space group P2_1_2_1_2_1_ (no. 19), *a* = 7.5679 (3) Å, *b* = 9.6137 (3) Å, *c* = 46.6023 (14) Å, *V* = 3390.6 (2) Å^3^, *Z* = 4, *T* = 291 (2) K, *μ* (Cu K*α*) = 0.734 mm^−1^, *Dcalc* = 1.279 g/mm^3^, 17740 reflections measured (7.588 ≤ 2*θ* ≤ 139.168), 6251 unique (*R*_int_ = 0.0376, *R*_sigma_ = 0.0396) which are used in all of the calculations. The final *R*_1_ was 0.0477 [I > 2*σ* (I)], and *wR*_2_ was 0.1591 (all data). Flack parameter: −0.07(14).

A colorless crystal of **2** was obtained from a mixture of MeOH and H_2_O. The crystal data were obtained using a Bruker Smart 1000 CCD with a graphite monochromator, using Cu K*α* radiation at 291 (2) K. The crystal was tested with a diffractometer using Olex2^32^, and the structure was solved through direct methods with the ShelXS^33^ structure solution program and refined with the ShelXL^33^ refinement package using least squares minimization. The crystallographic data for **2** were deposited in the Cambridge Crystallographic Data Centre (deposition number CCDC 1042328). Copies of these data can be obtained free of charge via the Internet at www.ccdc.cam.ac.uk/conts/retrieving.html or from the Cambridge Crystallographic Data Centre, 12 Union Road, Cambridge CB21EZ, UK [fax (+44) 1223 336 003; e-mail: deposit@ccdc.cam.ac.uk].

### Theoretical calculated and experimental observed ECD of 8

The phenomena of ECD have been extensive applied in the determination of the absolute configurations of natural chiral molecules^34^. The conformations were generated and optimized by Gaussian 09 package^35^. For the TD calculations Gaussian 09 was used. TDDFT calculations employed the B3LYP functional and the 6-311+g (d, 2p) basis set (Nstates = 40, root = 3). An overall ECD spectrum was generated on the basis of Boltzmann weighting of 5 individual conformers (SI-Table 1) applying a shift based upon the difference between observed and calculated UV spectra. Comparisons of the experimental and calculated spectra were done using SpecDis with UV shift (36 nm) and a half-bandwidth of 0.21 eV. Finally, the calculated ECD spectrum of conformer 4 was adjacent to experimental ECD data. Through comparison with the experimental ECD of **8**, the absolute configurations of **8** were assigned as 2*R*, 3*R*, 4*S*, 5*R*, and 6*S*, respectively.

### Determination of Cytotoxic Activities

As reported recently in the literatures, rocaglates exert significant cytotoxic activities^24^,^25^,^26^,^27^,^28^. In this investigation, the IC_50_ values in human leukemic (HL-60), colon cancer (HT-29), human breast cancer (MCF-7), and human liver hepatocellular carcinoma (HepG2) cell lines were determined to evaluate the cytotoxicity of the isolated compounds. The isolated compounds were evaluated based on their cytotoxic activities against the above-mentioned tumor cell lines. The silvestrol analogues exhibited more potent cytotoxic activity than the cyclopenta[*bc*]benzopyrans, as shown in Table 4. The cytotoxicity assay used in this study is based on the MTT method and was performed in 96-well microplates^36^. The cells were cultured in DMEM medium (Hyclone, Logan, UT, USA) with 10% foetal bovine serum in an atmosphere with 5% CO_2_ at 37 °C prior to the assay. Then, 150 *μ*L of the cell suspension was seeded into each well of 96-well the cell culture plates, and the cells were allowed to adhere for 12 h before testing. The initial density of the cells was 10^5^/mL. Each tumor cell line was exposed to each test compound at concentrations of 0.001, 0.01, 0.1, 1, and 10 *μ*M in triplicate for 48 h, and paclitaxel and *cis*-platinum were used as positive controls. Using the Reed-Muench method, the IC_50_ values were calculated based on the obatained cell viability^37^.

### Statistical analysis

Statistical analysis of the data was processed with GraphPad Prism 4.0 software. Statistical analysis of the data was expressed as mean ± SD. Values were analyzed by one-way analysis of variance (ANOVA) using SPSS version 12.0 software. *p* < 0.05 were considered statistically significant.

### Preparation of the acetyl derivatives of 1, 10, and 11

Compounds **1** (1.0 mg), **10** (2.0 mg), and **11** (1.3 mg) were acetylated with acetic anhydride (0.5 mL), CH_2_Cl_2_ (1 mL), and DMAP (0.5 mg), respectively, at room temperature for 2 h. The reaction products were purified by semi-preparative-HPLC (MeOH-H_2_O, 70%) to give compounds **1a** (0.5 mg, *t*_R_ = 8.1 min, 50.0% yield), **10a** (1.2 mg, *t*_R_ = 8.1 min, 60.0% yield), and **11a** (0.8 mg, *t*_R_ = 9.3 min, 61.5% yield).

#### Aglapervirisin A (1)

colourless powder; 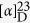 −82.1 (*c*, 0.11, MeOH); UV (MeOH) *λ*_*max*_ (log *ε*) 209 (4.73), 273 (3.86) nm; IR (KBr) *ν*_*max*_ 3424, 2944, 2840, 1620, 1516, 1454, 1385, 1252, 1220, 1201, 1148, 1114, 1049, 1007, 814, 696 cm^−1^; ECD (0.22 mg/mL, MeOH) *λ*_*max*_ (*ε*) 200 (24.30), 216 (−17.72); ^1^H and ^13^C NMR, see Table 1; negative ESIMS *m/z* 539.8 [M + Cl]^−^; positive ESIMS *m/z* 506.0 [M + H]^+^; HRESIMS *m/z* 719.2313 [M + Na]^+^ (calcd for C_36_H_40_O_14_Na, 719.2313).

#### Aglapervirisin B (2)

colourless crystals (MeOH); mp: 168–169 °C; 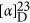 −28.3 (*c*, 0.10, MeOH); UV (MeOH) *λ*_*max*_ (log *ε*) 204 (4.85), 270 (3.49) nm; IR (KBr) *ν*_*max*_ 3485, 2933, 1633, 1532, 1455, 1200, 1150, 1100, 1050, 814, 702 cm^−1^; ECD (0.2 mg/mL, MeOH) *λ*_*max*_ (*ε*) 208 (−9.15), 226 (4.07); ^1^H and ^13^C NMR, see Table 2; negative ESIMS *m/z* 687.7 [M + Cl]^−^; positive ESIMS *m/z* 653.4 [M + H]^+^; HRESIMS *m/z* 653.2853 [M + H]^+^(calcd for C_38_H_41_N_2_O_8_, 653.2857); *m/z* 675.2672 [M + Na]^+^(calcd for C_38_H_40_N_2_O_8_Na, 675.2677).

#### Aglapervirisin C (3)

colourless powder; 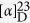 −22.1 (*c* 0.10, MeOH); UV (MeOH) *λ*_*max*_ (log *ε*) 203 (4.73), 269 (3.43) nm; IR (KBr) *ν*_*max*_ 3449, 2348, 1629, 1513, 1463, 1385, 1027, 915, 672 cm^−1^; ECD (0.2 mg/mL, MeOH) *λ*_*max*_ (*ε*) 212 (−0.83), 227 (0.24); ^1^H and ^13^C NMR, see Table 2; negative ESIMS *m/z* 687.5 [M + Cl]^−^; positive ESIMS *m/z* 653.5[M + H]^+^; HRESIMS *m/z* 653.2861 [M + H]^+^(calcd for C_38_H_41_N_2_O_8_, 653.2857); *m/z* 675.2675 [M + Na]^+^(calcd for C_38_H_40_N_2_O_8_Na, 675.2677).

#### Aglapervirisin D (4)

colourless powder; 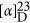 −3.4 (*c* 0.42, MeOH); UV (MeOH) *λ*_*max*_ (log *ε*) 204 (4.85) nm; IR (KBr) *ν*_*max*_ 3462, 2930, 1732, 1621, 1517, 1455, 1256, 1149, 1087, 1023, 833, 701 cm^−1^; ^1^H and ^13^C NMR, see Table 2; negative ESIMS *m/z* 685.5 [M + Cl]^−^; positive ESIMS *m/z* 651.5 [M + H]^+^; HRESIMS *m/z* 651.2694 [M + H]^+^ (calcd for C_38_H_39_N_2_O_8_, 651.2701); *m/z* 673.2516 [M + Na]^+^ (calcd for C_38_H_38_N_2_O_8_Na, 673.2520).

#### Aglapervirisin E (5)

colourless powder; 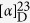 −4.4 (*c* 0.28, MeOH); UV (MeOH) *λ*_*max*_ (log *ε*) 203 (4.82), 273 (3.65) nm; IR (KBr) ν_*max*_ 3462, 2936, 1623, 1518, 1457, 1386, 1256, 1203, 1151, 1088, 835, 702 cm^−1^; ^1^H and ^13^C NMR, see Table 3; negative ESIMS *m/z* 717.5 [M + Cl]^−^; positive ESIMS *m/z* 683.5 [M + H]^+^; HRESIMS *m/z* 683.2964 [M + H]^+^ (calcd for C_39_H_43_N_2_O_9_, 683.2963); *m/z* 705.2784 [M + Na]^+^ (calcd for C_38_H_40_N_2_O_8_Na, 705.2783).

#### Aglapervirisin F (6)

colourless powder; 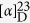
−5.4 (*c* 0.28, MeOH); UV (MeOH) *λ*_*max*_ (log *ε*) 210 (4.58) nm; IR (KBr) *ν*_*max*_ 3445, 2925, 2853, 2351, 1623, 1507, 1456, 1385, 1149, 823, 697, 663 cm^−1^; ^1^H and ^13^C NMR, see Table 3; negative ESIMS *m/z* 703.2 [M + Cl]^−^; positive ESIMS *m/z* 669.2 [M + H]^+^; HRESIMS *m/z* 669.2801 [M + H]^+^ (calcd for C_38_H_41_N_2_O_9_, 669.2807); *m/z* 691.2625 [M + Na]^+^ (calcd for C_38_H_40_N_2_O_9_Na, 691.2626).

#### Aglapervirisin G (7)

colourless powder; 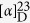 −35.8 (*c* 0.11, MeOH); UV (MeOH) λ_*max*_ (log *ε*) 203 (4.82), 273 (3.65) nm; IR (KBr) ν_*max*_ 3446, 2932, 1620, 1457, 1385, 1147, 1099, 1029, 830, 699 cm^−1^; ECD (0.22 mg/mL, MeOH) *λ*_*max*_ (*ε*) 214 (0.69), 236 (−1.12), 243 (−1.41); ^1^H and ^13^C NMR, see Table 2; negative ESIMS *m/z* 687.3 [M + Cl]^−^; positive ESIMS *m/z* 653.3 [M + H]^+^; HRESIMS *m/z* 653.2859 [M + H]^+^ (calcd for C_38_H_41_N_2_O_8_, 653.2857); *m/z* 675.2671 [M + Na]^+^ (calcd for C_38_H_40_N_2_O_8_Na, 675.2677).

#### Aglapervirisin H (8)

colourless powder; 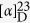 +96.9 (*c* 0.10, MeOH); UV (MeOH) *λ*_*max*_ (log *ε*) 203 (4.73), 273 (3.54), 279 (3.59) nm; IR (KBr) *ν*_*max*_ 3443, 2923, 1751, 1644, 1546, 1384, 1235, 1151, 1029, 703 cm^−1^; ECD (0.2 mg/mL, MeOH) *λ*_*max*_ (*ε*) 200 (10.50), 218 (1.83), 242 (8.15); ^1^H and ^13^C NMR, see Table 3; negative ESIMS *m/z* 612.4 [M + Cl]^−^; positive ESIMS *m/z* 578.4 [M + H]^+^; HRESIMS *m/z* 578.2381 [M + H]^+^ (calcd for C_32_H_36_NO_9_, 578.2385); *m/z* 600.2202 [M + Na]^+^ (calcd for C_32_H_35_NO_9_Na, 600.2204).

#### Aglapervirisin I (9)

colourless powder; 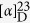 +85.4 (*c,* 0.15, MeOH); UV (MeOH) *λ*_*max*_ (log *ε*) 205 (4.69), 210 (4.69), 273 (3.27), 279 (3.17) nm; IR (KBr) *ν*_*max*_ 3446, 2931, 1623, 1519, 1457, 1149, 1007, 841, 701 cm^−1^; ECD (0.3 mg/mL, MeOH) *λ*_*max*_ (*ε*) 200 (14.90), 217 (2.05), 242 (9.90); ^1^H and ^13^C NMR, see Table 3; negative ESIMS *m/z* 584.4 [M + Cl]^−^; positive ESIMS *m/z* 550.4 [M + H]^+^; HRESIMS *m/z* 550.2431 [M + H]^+^ (calcd for C_31_H_36_NO_8_, 550.2435); *m/z* 572.2253 [M + Na]^+^ (calcd for C_31_H_35_NO_8_Na, 572.2255).

Compound **1a**: white amorphous powder. 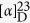 −44.4 (*c*, 0.05, MeOH); ^1^H NMR (CDCl_3_, 500 MHz), see Figure S10-1 in Supporting Information. HRESIMS *m/z* [M + NH_4_]^+^ 756.2857 (calcd for C_38_H_46_O_15_N, 756.2862).

Compound **10**: white amorphous powder. ^1^H NMR (CDCl_3_, 500 MHz), see Figure S11-1 in Supporting Information. HRESIMS *m/z* [M + H]^+^ 655.2387 (calcd for C_34_H_39_O_13_, 655.2385).

Compound **10a**: white amorphous powder. 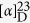 −52.9 (*c*, 0.12, MeOH); ^1^H NMR (CDCl_3_, 500 MHz), see Figure S11-3 in Supporting Information. HRESIMS *m/z* [M + NH_4_]^+^ 756.2864 (calcd for C_38_H_46_O_15_N, 756.2862).

Compound **11**: white amorphous powder. ^1^H NMR (CDCl_3_, 500 MHz), ^13^C NMR (CDCl_3_, 125 MHz), see Figure S12-1 in Supporting Information. HRESIMS *m/z* [M + H]^+^ 655.2380 (calcd for C_34_H_39_O_13_, 655.2385).

Compound **11a**: white amorphous powder. 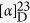 −61.5 (*c*, 0.08, MeOH); ^1^H NMR (CDCl_3_, 500 MHz), see Figure S12-3 in Supporting Information. HRESIMS *m/z* [M + NH_4_]^+^ 756.2859 (calcd for C_38_H_46_O_15_N, 756.2862).

Compound **12**: white amorphous powder. ^1^H NMR (CDCl_3_, 500 MHz), ^13^C NMR (CDCl_3_, 125 MHz), see Figure S13 in Supporting Information. HRESIMS *m/z* [M + H]^+^ 653.2854 (calcd for C_38_H_41_N_2_O_8_, 653.2857).

Compound **13**: white amorphous powder. ^1^H NMR (CDCl_3_, 500 MHz), ^13^C NMR (CDCl_3_, 125 MHz), see Figure S14 in Supporting Information. HRESIMS *m/z* [M + H]^+^ 653.2856 (calcd for C_38_H_41_N_2_O_8_, 653.2857).

Compound **14**: white amorphous powder. ^1^H NMR (CDCl_3_, 500 MHz), ^13^C NMR (CDCl_3_, 125 MHz), see Figure S15 in Supporting Information. HRESIMS *m/z* [M + H]^+^ 651.2703 (calcd for C_38_H_39_N_2_O_8_, 651.2701).

## Additional Information

**How to cite this article**: An, F.-L. *et al*. Cytotoxic Rocaglate Derivatives from Leaves of *Aglaia perviridis. Sci. Rep.* **6**, 20045; doi: 10.1038/srep20045 (2016).

## Supplementary Material

Supplementary Information

## Figures and Tables

**Figure 1:**
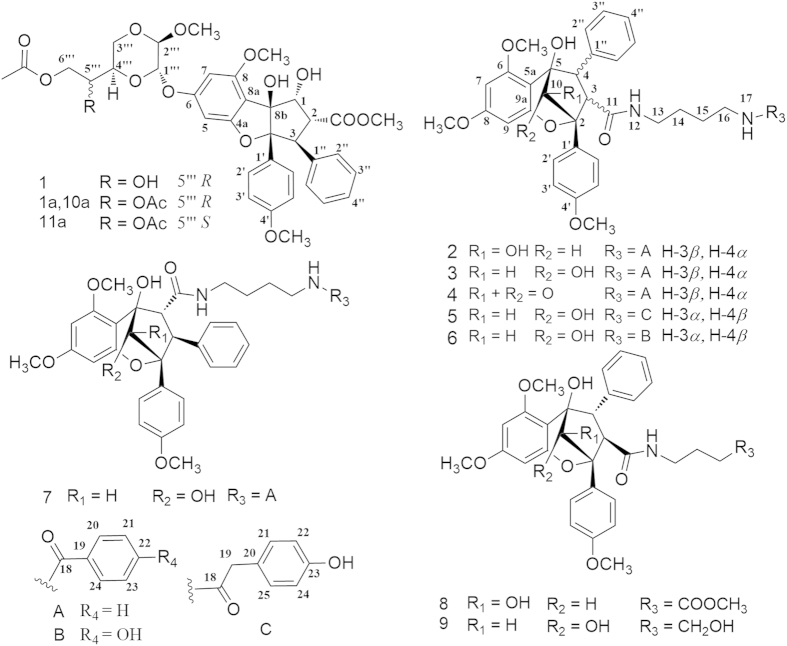
Chemical structures of compounds 1–9, 1a, 10a and 11a.

**Table 1:** ^1^H NMR and ^13^C NMR Spectroscopic Data for Compound 1.

**Proton No.**	**1**^a^		**Proton No.**	**1**^a^	
	***δ***_**H**_	**δ**_**C**_		***δ***_**H**_	**δ**_**C**_
1	5.11 d (6.5)	79.8	3′5′	6.72 d (8.0)	112.7
2	3.91 dd (14.0 7.0)	50.5	4′		158.8
3	4.32 d (14.5)	55.0	1″		136.9
3a		102.0	2″6″	6.88 m	127.9
4a		160.7	3″5″	7.11 m	127.7
5	6.46 d (2.0)	93.4	4″	7.11 m	126.6
6		159.7	1′′′	5.41 s	93.7
7	6.31 d (2.0)	93.6	2′′′	4.67 s	95.3
8		157.2	3′′′	4.25 t (7.5) 3.61 d (8.5)	59.0
8a		110.0	4′′′	3.83 td (6.0, 2.0)	68.6
8b		93.5	5′′′	4.24 td (11.0, 2.0)	67.1
8-OMe	3.94 s	56.0	6′′′	4.08 dd (11.0, 6.5) 3.99 dd (11.0, 6.5)	64.1
4′-OMe	3.77 s	55.1	COOCH_3_-2	3.67 s	51.9
2′′′-OMe	3.56 s	55.1	COOCH_3_-2		170.4
1′		126.4	6′′′-CH_3_CO	1.89 s	20.5
2′6′	7.15 d (8.0)	129.1	6′′′-CH_3_CO		171.0

^a^^1^H NMR spectra measured at 500 MHz, ^13^C NMR spectra measured at 125 MHz; ^a^was obtained in CDCl_3_. The assignments are based on the 2D-NMR spectra.

**Table 2:** ^1^H NMR and ^13^C NMR Spectroscopic Data for Compounds 2–4 and 7.

**Proton No.**	**2**^a^		**3**^a^		**4**^a^		**7**^a^	
	***δ***_**H**_	***δ***_**C**_	***δ***_**H**_	***δ***_**C**_	***δ***_**H**_	***δ***_**C**_	***δ***_**H**_	***δ***_**C**_
2		87.3		84.5		98.6		87.5
3	4.29 d (6.5)	58.4	3.51 d (5.5)	54.7	3.33 d (11.5)	54.3	4.25 d (9.5)	62.9
4	4.42 d (6.5)	60.5	4.32 d (5.5)	59.8	4.66 d (11.5)	59.5	3.09 d (9.5)	53.8
5		80.0		79.8		89.8		80.9
5a		112.3		108.2		105.7		101.8
6		156.2		158.3		159.0		159.5
7	6.10 d (2.0)	92.9	6.12 d (2.0)	93.3	6.17 d (2.0)	93.6	6.08 d (2.0)	93.2
8		160.7		160.7		165.3		161.2
9	6.18 d (2.0)	94.3	6.20 d (2.0)	93.9	6.34 d (2.0)	90.4	6.20 d (2.0)	94.8
9a		153.2		153.6		160.6		154.2
10	4.19 s	82.6	4.68 s	76.1		209.5	4.78 d (5.5)	74.2
11		169.7		168.5		168.6		169.3
13	3.27 m 2.88 m	38.8	3.32 m 2.92 m	39.1	3.27 m 2.85 m	39.3	3.15 m 3.20 m	39.4
14	1.33 m	26.2	1.51 m	27.7	1.16 m	26.3	1.50 m	26.7
15	1.33 m	27.5	1.40 m	26.3	1.24 m 1.16 m	27.2	1.50 m	27.5
16	3.34m	39.8	3.42 m	39.9	3.27 m	39.9	3.41 m	39.7
18		167.6		167.7		167.8		167.7
19		134.8		134.7		134.7		134.7
20, 24	7.77 d (7.5)		7.76 d (7.0)	128.6	7.80 d (7.5)	127.1	7.76 d (7.0)	127.1
21, 23	7.40 t (7.5)	128.6	7.43 t (7.0)	127.0	7.45 t (7.5)	129.0	7.42 t (7.0)	128.7
22	7.48 t (7.5)	131.4	7.50 t (7.0)	131.5	7.51 t (7.5)	131.6	7.50 t (7.0)	131.5
6-OMe	3.87 s	56.3	3.86 s	56.1	3.78 s	55.9	3.81 s	56.1
8-OMe	3.72 s	55.5	3.72 s	55.5	3.85 s	55.9	3.71 s	55.3
4′-OMe	3.82 s	55.5	3.83 s	55.5	3.74 s	55.4	3.68 s	55.4
NH-12	5.25 t (5.5)		5.08 t (5.5)		5.88 t (6.5)		6.48 br t (6.0)	
NH-17	6.47 t (5.5)		6.48 t (5.5)		6.40 t (6.5)		5.99 br t (6.0)	
1′		129.9		131.1		125.4		129.5
2′6′	7.84 d (8.5)	128.5	7.71 d (8.5)	128.8	7.30 d (8.5)	127.5	7.29 d (8.5)	128.8
3′5′	7.02 d (8.5)	114.2	7.03 d (8.5)	114.8	6.87 d (8.5)	114.0	6.66 d (8.5)	113.4
4′		159.8		159.8		159.8		159.0
1″		140.9		141.5		138.9		140.5
2″6″	7.52 d (7.5)	130.1	7.38 d (7.0)	129.2	7.04 d (8.5)	128.3	6.90 d (7.0)	129.4
3″5″	7.35 t (7.5)	128.4	7.37 t (7.0)	127.2	7.20 m (overlap)	129.0	7.00 t (7.0)	128.2
4″	7.26 brd s	126.9	7.31 t (7.0)	127.1	7.20 m (overlap)	127.5	6.95 t (7.0)	126.5

^a^^1^H NMR spectra measured at 500 MHz, ^13^C NMR spectra measured at 125 MHz; ^a^was obtained in CDCl_3_. The assignments are based on the 2D-NMR spectra.

**Table 3:** 1H NMR and ^13^C NMR Spectroscopic Data for Compounds 5–6 and 8–9.

**Proton No.**	**5**^**a**^		**Proton No.**	**6**^**b**^		**Proton No.**	**8**^**a**^		**Proton No.**	**9**^**a**^	
	***δ***_**H**_ **multi**	***δ***_**C**_		***δ***_**H**_	***δ***_**C**_		***δ***_**H**_	**δ**_**C**_		***δ***_**H**_	**δ**_**C**_
2		87.0	2		85.0	2		87.0	2		87.0
3	3.86 d (8.5)	61.8	3	4.09 d (8.5)	62.2	3	3.88 d (8.5)	61.8	3	3.88 d (8.5)	62.0
4	4.06 d (8.5)	61.8	4	3.91 d (8.5)	62.5	4	4.10 d (8.5)	62.0	4	4.12 d (8.5)	61.8
5		83.6	5		88.0	5		83.3	5		83.4
5a		106.1	5a		107.6	5a		106.1	5a		106.2
6		159.0	6		154.2	6		159.0	6		159.0
7	5.78 d (2.0)	93.0	7	6.04 d (2.0)	95.0	7	6.04 d (2.0)	93.0	7	5.77 d (2.0)	92.9
8		161.2	8		162.7	8		161.2	8		161.2
9	6.03 d (2.0)	94.0	9	5.89 d (2.0)	93.6	9	5.78 d (2.0)	94.0	9	6.03 d (2.0)	93.9
9a		153.0	9a		160.4	9a		153.0	9a		153.0
10	4.89 s	78.8	10	4.68 s	79.7	10	4.89 s	79.0	10	4.87 d (5.0)	78.9
11		173.6	11		175.8	11		173.8	11		173.3
13	2.75 m 2.85 m	39.1	13	2.91 m 2.83 m	40.2	13	2.96 m 2.86 m	39.1	13	2.90 m 2.85 m	39.5
14	0.94 m	26.4	14	1.15 m	27.3	14	1.36 m	24.2	14	1.18 m	29.7
15	1.01 m	26.1	15	1.28 m	27.6	15	1.94 t (7.3)	31.3	15	1.10 m	25.7
16	2.95 m	39.3	16	3.17m	40.4	16		173.4	16	3.40 m	62.5
18		172.0	18		170.0	COOCH3	3.62	51.7	5-OH	5.44 s	
19	3.45 s	43.1	19		126.7				10-OH	5.18 d (5.0)	
20		126.8	20, 24	7.67 d (8.5)	130.4						
21, 25	7.06 d (8.5)	130.8	21, 23	6.81 d (8.5)	114.4						
22, 24	6.84 d (8.5)	116.5	22		161.9						
23		155.6									
6-OMe	3.09 s	55.9	6-OMe	3.10 s	56.4	6-OMe	3.08 s	55.9	6-OMe	3.07 s	55.9
8-OMe	3.71 s	55.5	8-OMe	3.71 s	55.8	8-OMe	3.71 s	55.5	8-OMe	3.69 s	55.5
4′-OMe	3.78 s	55.5	4′-OMe	3.76 s	55.8	4′-OMe	3.82 s	55.4	4′-OMe	3.81 s	55.5
NH-12	5.50 t (5.5)		NH-12	5.25 t (5.5)		NH-12	6.58 m		NH-12	6.74 t (6.0)	
NH-17	6.68 t (5.5)		NH-17	6.47 t (5.5)							
1′		130.3	1′		131.7	1′		130.1	1′		130.3
2′6′	7.68 d (8.5)	129.4	2′6′	7.69 d (8.5)	130.2	2′6′	7.71 d (8.3)	129.3	2′6′	7.74 d (8.6)	129.4
3′5′	6.86 d (8.5)	113.9	3′5′	6.90 d (8.5)	116.1	3′5′	6.92 d (8.3)	114.0	3′5′	6.92 d (8.6)	114.0
4′		159.8	4′		161.1	4′		159.9	4′		159.8
1″		136.9	1″		138.3	1″		136.8	1″		137.0
2″6″	6.97 m	128.7	2″6″	6.97 m	129.8	2″6″	6.97 m	128.7	2″6″	6.98 m	128.7
3″5″	7.16 m	128.0	3″5″	7.17 m	128.8	3″5″	7.17 m	127.9	3″5″	7.14 m	127.9
4″	7.16 m	127.3	4″	7.17 m	128.2	4″	7.17 m	127.3	4″	7.14 m	127.2

^a,b 1^H NMR spectra measured at 500 MHz; ^13^C NMR spectra measured at 125 MHz; ^a^ was obtained in CDCl_3;_^b^ was obtained in methanol-*d*_4_. The assignments are based on the 2D-NMR spectra.

**Table 4:** Cytotoxic Evaluation of Compounds 1–5, 7–11 and 13–14^a^.

**Compound**	**HepG2**^**b**^	**HL-60**^**c**^	**MCF-7**^**e**^	**HT-29**^**d**^
**1**	0.014	0.009	0.009	0.008
**2**	>50.00	>50.00	>50.00	>50.00
**3**	>50.00	>50.00	>50.00	>50.00
**4**	>50.00	>50.00	>50.00	>50.00
**5**	10.9	2.2	8.5	1.4
**7**	>50.00	>50.00	>50.00	>50.00
**8**	>50.00	>50.00	>50.00	>50.00
**9**	27.7	29.4	36.0	23.1
**10**	0.016	0.013	0.008	0.008
**11**	0.006	0.007	0.008	0.008
**13**	23.0	19.5	31.1	16.3
**14**	>50.00	>50.00	>50.00	>50.00
Taxol^*f*^				0.002
*cis*-platinum^*g*^	8.2	2.5	6.4	

^a^Results are expressed as IC_50_ values (*μ*M).

^b,c,d^Compounds **2**–**4**, **7**–**8**, **14**, were inactive against HepG2 cells (IC_50_ > 50 *μ*M).

^f^Used as a positive control for the cytotoxicity assay against HT-29 cells.

^g^Used as a positive control for the cytotoxicity assay against HepG2, HL-60, and MCF-7 cells.
