# Supplementary material for: Cytotoxic Rocaglate Derivatives from Leaves of Aglaia perviridis
Source: Sci Rep. 2016 Jan 28;6:20045. doi: 10.1038/srep20045 (PMC4730247; doi:10.1038/srep20045)
Supplement: Supplementary Information [file srep20045-s1.doc]

S[upplementary Information](http://www.nature.com/srep/authors/submit.html#supplementary-info)

Cytotoxic Rocaglate Derivatives from the Leaves of *Aglaia perviridis*

Fa-Liang An, Xiao-Bing Wang, Hui Wang, Zhong-Rui Li, Ming-Hua Yang, Jun Luo*, Ling-Yi Kong*

State Key Laboratory of Natural Medicines, Department of Natural Medicinal Chemistry, China Pharmaceutical University, 24 Tong Jia Xiang, Nanjing 210009, People’s Republic of China

*Tel/Fax: +86-25-83271405. E-mail: [cpu_lykong@126.com](mailto:cpu_lykong@126.com) (L.-Y. Kong)

*Tel/Fax: +86-25-83271704. E-mail:luojun1981ly@163.com (J. Luo)

**Supporting information list.**

**Figure S1-1.** ^1^H NMR spectrum of compound **1** (CDCl_3_, 500 MHz).

**Figure S1-2.** ^13^C NMR spectrum of compound **1** (CDCl_3_, 125 MHz).

**Figure S1-3.** HSQC spectrum of compound **1** (CDCl_3_).

**Figure S1-4.** HMBC spectrum of compound **1** (CDCl_3_).

**Figure S1-5.** ROESY spectrum of compound **1** (CDCl_3_).

**Figure S1-6.** ^1^H-^1^H COSY spectrum of compound **1** (CDCl_3_).

**Figure S1-7.** HRESIMS of compound **1**.

**Figure S2-1.** ^1^H NMR spectrum of compound **2** (CDCl_3_, 500 MHz).

**Figure S2-2.** ^13^C NMR spectrum of compound **2** (CDCl_3_, 125 MHz).

**Figure S2-3.** HSQC spectrum of compound **2** (CDCl_3_).

**Figure S2-4.** HMBC spectrum of compound **2** (CDCl_3_).

**Figure S2-5.** ROESY spectrum of compound **2** (CDCl_3_).

**Figure S2-6.** HRESIMS of compound **2**.

**Figure S3-1.** ^1^H NMR spectrum of compound **3** (CDCl_3_, 500 MHz).

**Figure S3-2.** ^13^C NMR spectrum of compound **3** (CDCl_3_, 125 MHz).

**Figure S3-3.** HSQC spectrum of compound **3** (CDCl_3_).

**Figure S3-4.** HMBC spectrum of compound **3** (CDCl_3_,).

**Figure S3-5.** ROESY spectrum of compound **3** (CDCl_3_).

**Figure S3-6.** HRESIMS of compound **3**.

**Figure S4-1.** ^1^H NMR spectrum of compound **4** (CDCl_3_, 500 MHz).

**Figure S4-2.** ^13^C NMR spectrum of compound **4** (CDCl_3_, 125 MHz).

**Figure S4-3.** HSQC spectrum of compound **4** (CD_3_OD).

**Figure S4-4.** HMBC spectrum of compound **4** (CD_3_OD).

**Figure S4-5.** ROESY spectrum of compound **4** (CD_3_OD).

**Figure S4-6.** HRESIMS of compound **4**.

**Figure S5-1.** ^1^H NMR spectrum of compound **5** (CDCl_3_, 500 MHz).

**Figure S5-2.** ^13^C NMR spectrum of compound **5** (CDCl_3_, 125 MHz).

**Figure S5-3.** HSQC spectrum of compound **5** (CDCl_3_).

**Figure S5-4.** HMBC spectrum of compound **5** (CDCl_3_).

**Figure S5-5.** ROESY spectrum of compound **5** (CDCl_3_).

**Figure S5-6.** HRESIMS of compound **5**.

**Figure S6-1.** ^1^H NMR spectrum of compound **6** (CDCl_3_, 500 MHz).

**Figure S6-2.** ^13^C NMR spectrum of compound **6** (CDCl_3_, 125 MHz).

**Figure S6-3.** HSQC spectrum of compound **6** (CDCl_3_).

**Figure S6-4.** HMBC spectrum of compound **6** (CDCl_3_).

**Figure S6-5.** ROESY spectrum of compound **6** (CDCl_3_).

**Figure S6-6.** HRESIMS of compound **6**.

**Figure S7-1.** ^1^H NMR spectrum of compound **7** (CDCl_3_, 500 MHz).

**Figure S7-2.** ^13^C NMR spectrum of compound **7** (CDCl_3_, 125 MHz).

**Figure S7-3.** HSQC spectrum of compound **7** (CDCl_3_).

**Figure S7-4.** HMBC spectrum of compound **7** (CDCl_3_).

**Figure S7-5.** ROESY spectrum of compound **7** (CDCl_3_).

**Figure S7-6.** HRESIMS of compound **7**.

**Figure S8-1.** ^1^H NMR spectrum of compound **8** (CDCl_3_, 500 MHz).

**Figure S8-2.** ^13^C NMR spectrum of compound **8** (CDCl_3_, 125 MHz).

**Figure S8-3.** HSQC spectrum of compound **8** (CDCl_3_).

**Figure S8-4.** HMBC spectrum of compound **8** (CDCl_3_).

**Figure S8-5.** ROESY spectrum of compound **8** (CDCl_3_).

**Figure S8-6.** HRESIMS of compound **8**.

**Figure S9-1.** ^1^H NMR spectrum of compound **9** (CDCl_3_, 500 MHz).

**Figure S9-2.** ^13^C NMR spectrum of compound **9** (CDCl_3_, 125 MHz).

**Figure S9-3.** HSQC spectrum of compound **9** (CDCl_3_).

**Figure S9-4.** HMBC spectrum of compound **9** (CDCl_3_).

**Figure S9-5.** ROESY spectrum of compound **9** (CDCl_3_).

**Figure S9-6.** HRESIMS of compound **9**.

**Figure S10-1.** ^1^H NMR spectrum of compound **1a** (CDCl_3_, 500 MHz).

**Figure S10-2.** HRESIMS of compound **1a**.

**Figure S11-1.** ^1^H NMR spectrum of compound **10** (CDCl_3_, 500 MHz).

**Figure S11-2.** HRESIMS of compound **10**.

**Figure S11-3.** ^1^H NMR spectrum of compound **10a** (CDCl_3_, 500 MHz).

**Figure S11-4.** HRESIMS of compound **10a**.

**Figure S12-1.** ^1^H NMR spectrum of compound **11** (CDCl_3_, 500 MHz).

**Figure S12-2.** HRESIMS of compound **11**.

**Figure S12-3.** ^1^H NMR spectrum of compound **11a** (CDCl_3_, 500 MHz).

**Figure S12-4.** HRESIMS of compound **11a**.

**Figure S13-1.** ^1^H NMR spectrum of compound **12** (CDCl_3_, 500 MHz).

**Figure S13-2.** HRESIMS of compound **12**.

**Figure S14-1.** ^1^H NMR spectrum of compound **13** (CDCl_3_, 500 MHz).

**Figure S14-2.** HRESIMS of compound **13**.

**Figure S15-1.** ^1^H NMR spectrum of compound **14** (CDCl_3_, 500 MHz).

**Figure S15-2.** HRESIMS of compound **14**.

**Figure S16.** Structures of compound **1**, **1a**, **10**, **10a**, **11**, **11a**.

**Table 1.** Conformers of compound **8** and its calculated ECD data.

**Table 2.** Optimized Z-Matrixes of compound **8** in the Gas Phase (Å) at B3LYP/6-311G (d, p) level.

**Figure S1-1.** ^1^H NMR spectrum of compound **1** (CDCl_3_, 500 MHz).


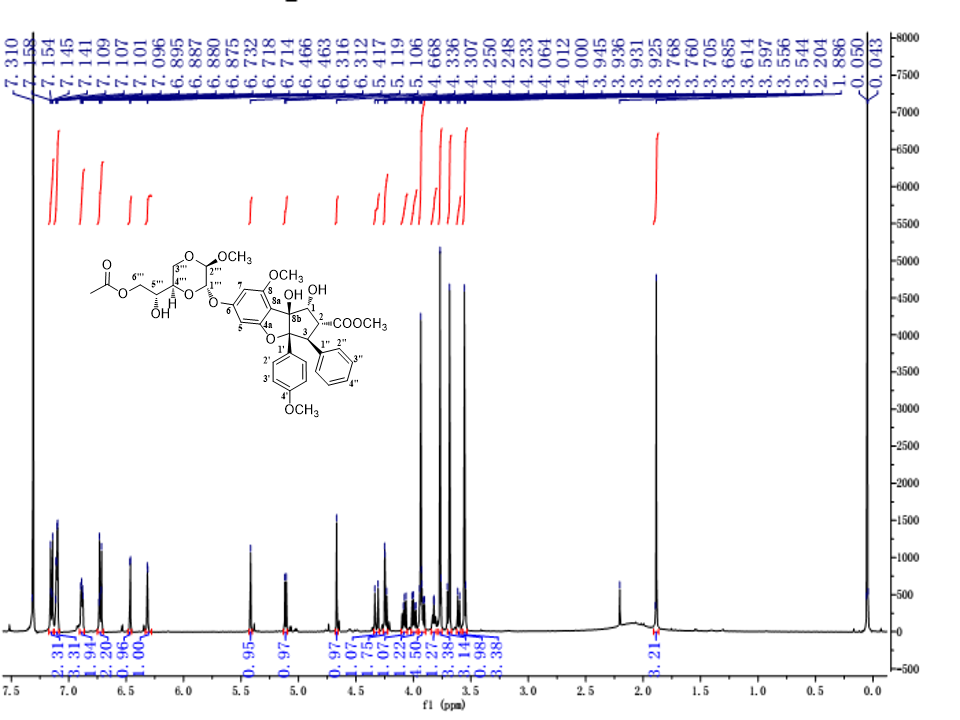


**Figure S1-2.** ^13^C NMR spectrum of compound **1** (CDCl_3_, 125 MHz).


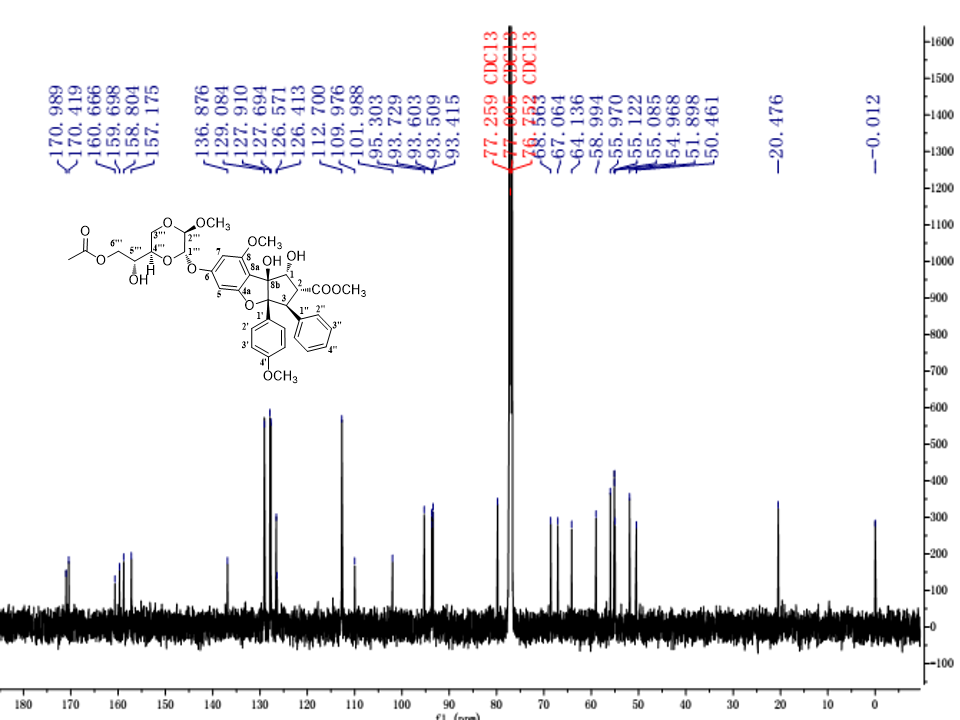


**Figure S1-3.** HSQC spectrum of compound **1** (CDCl_3_).

**
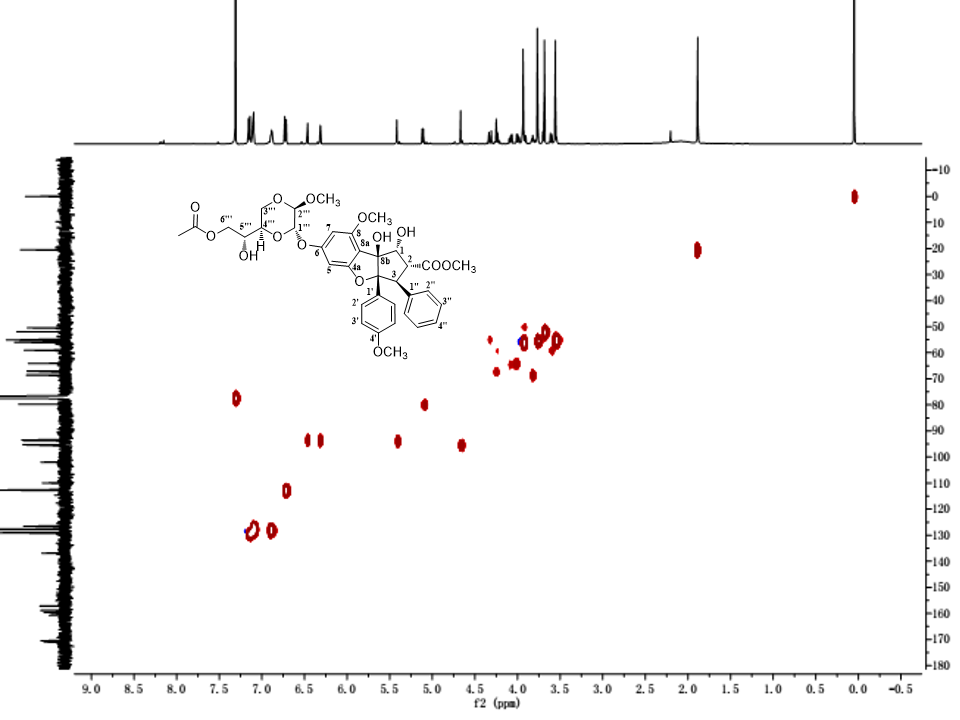
**

**Figure S1-4.** HMBC spectrum of compound **1** (CDCl_3_).


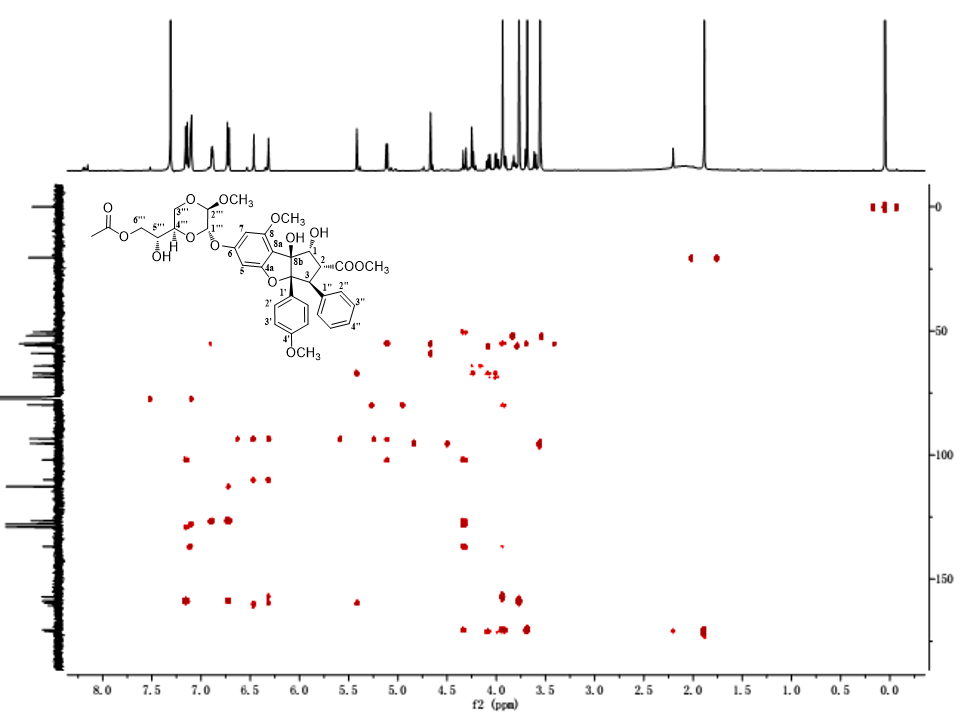


**Figure S1-5.** ROESY spectrum of compound **1** (CDCl_3_).


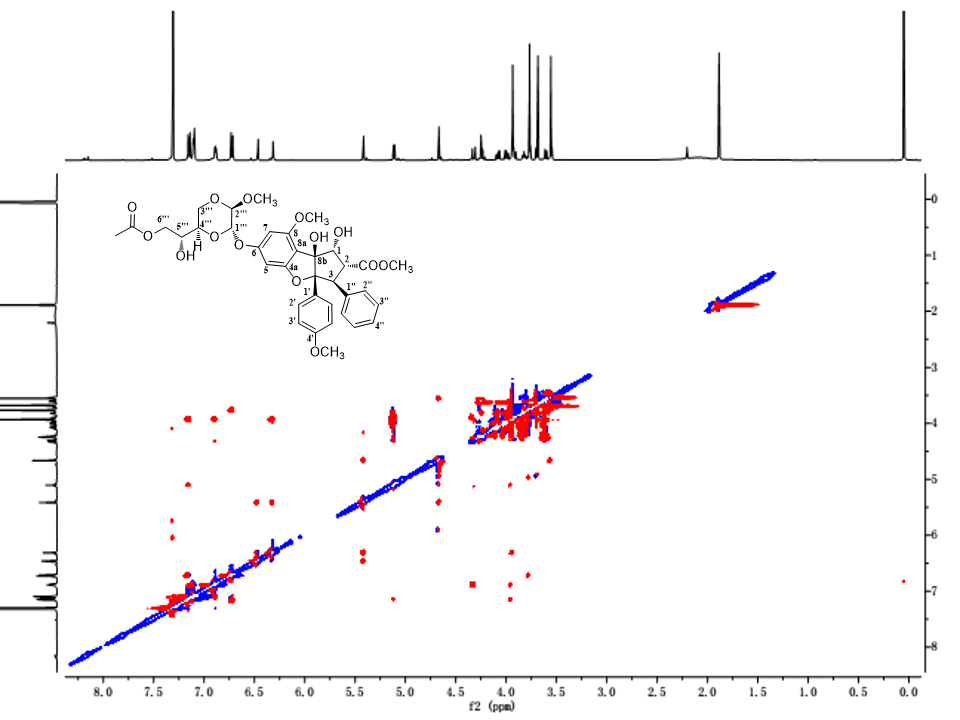


**Figure S1-6.** HRESIMS of compound **1**.


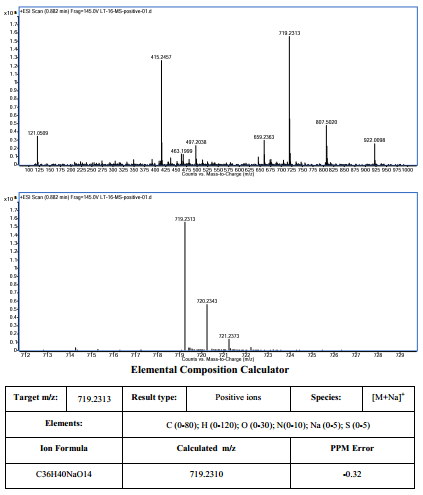


**Figure S2-1.** ^1^H NMR spectrum of compound **2** (CDCl_3_, 500 MHz).

**
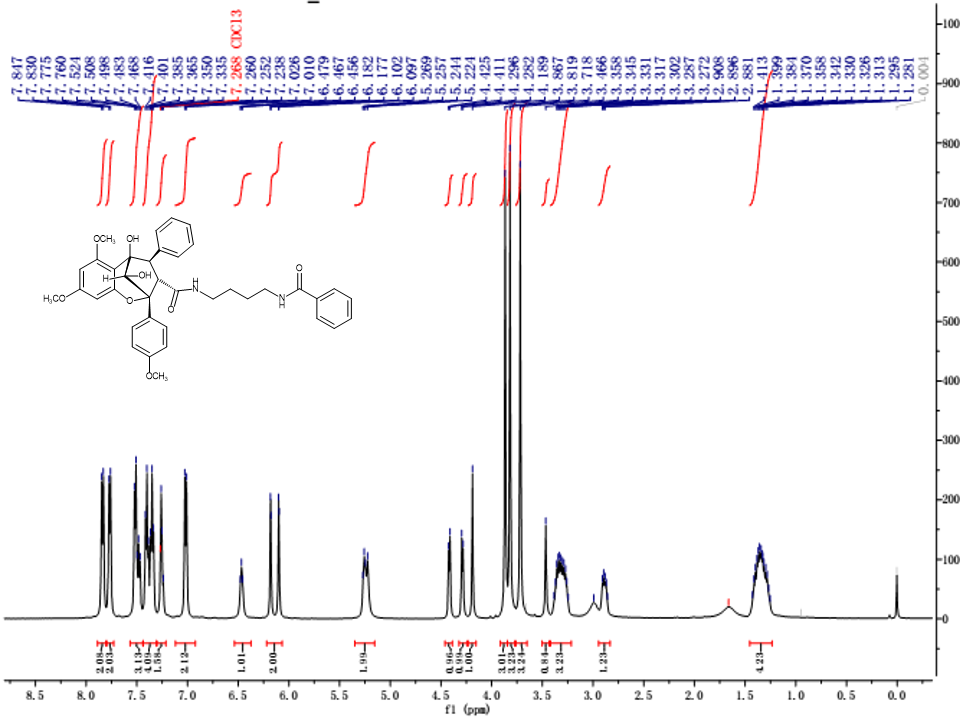
**

**Figure S2-2.** ^13^C NMR spectrum of compound **2** (CDCl_3_, 125 MHz).


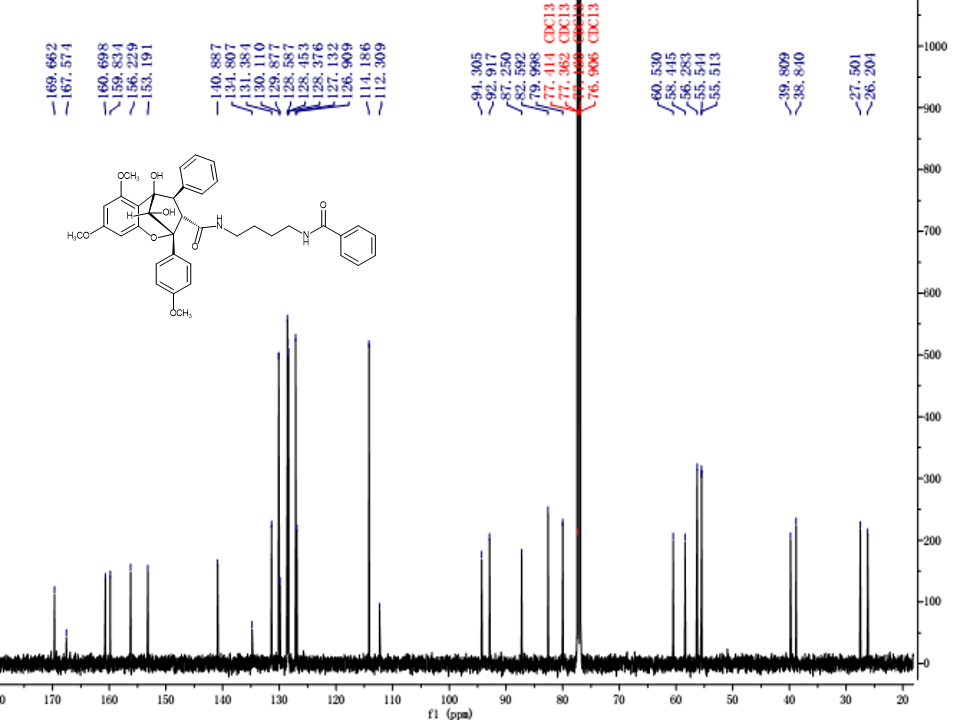


**Figure S2-3.** HSQC spectrum of compound **2** (CDCl_3_).

**
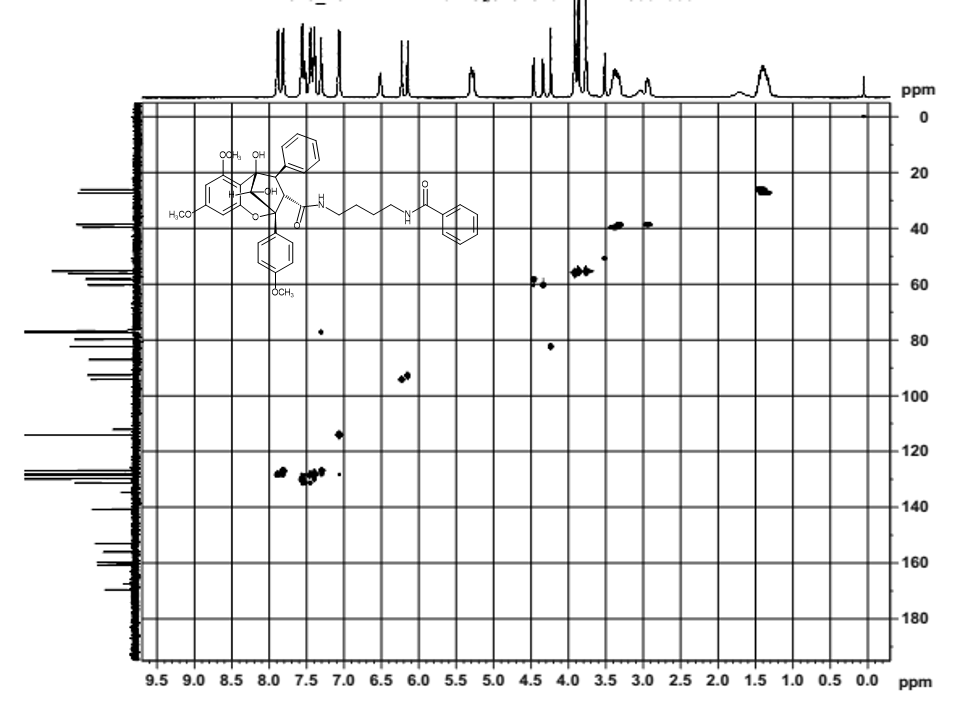
**

**Figure S2-4.** HMBC spectrum of compound **2** (CDCl_3_).


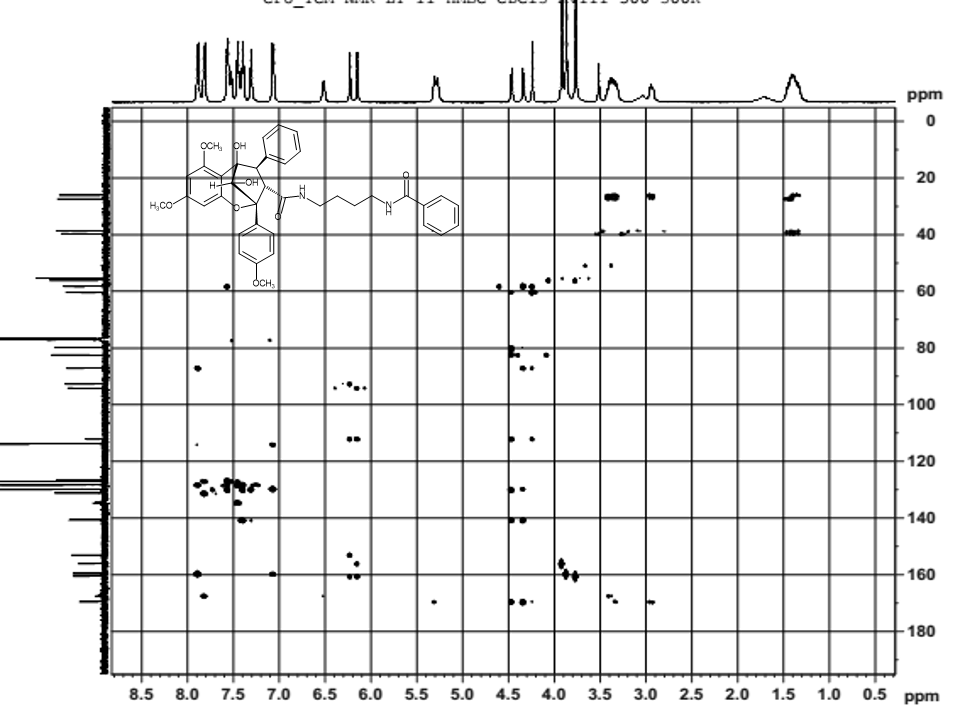


**Figure S2-5.** ROESY spectrum of compound **2** (CDCl_3_).


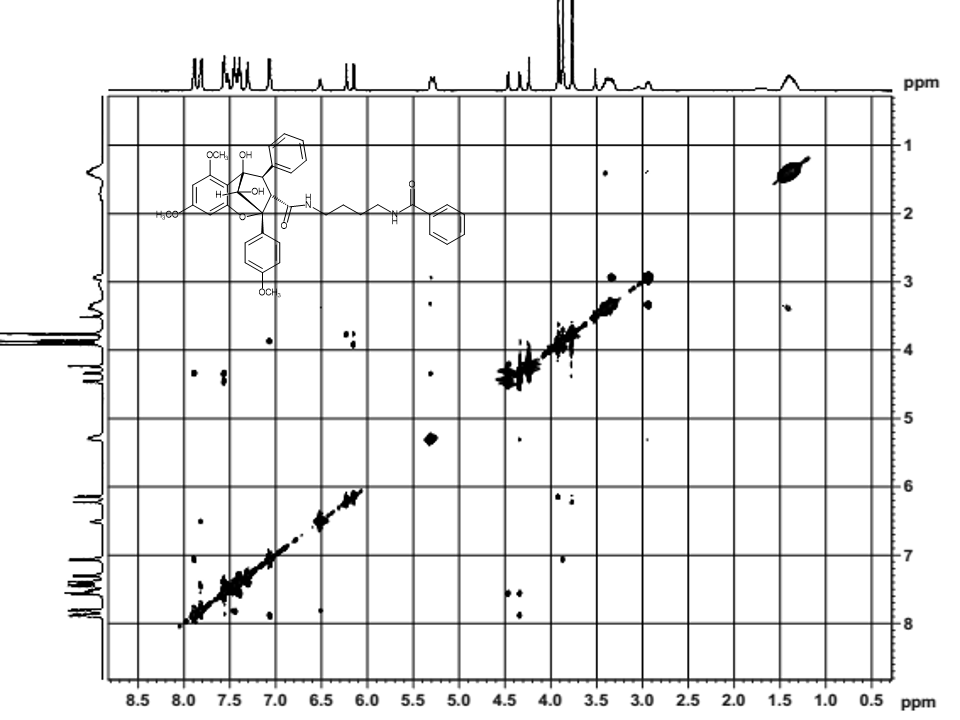


**Figure S2-7.** HRESIMS of compound **2**.


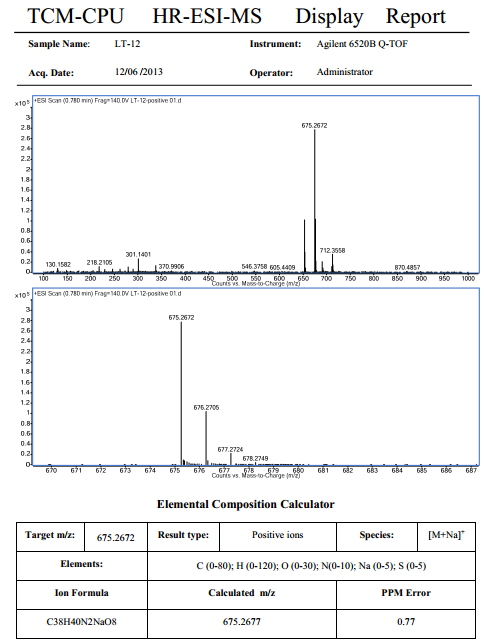

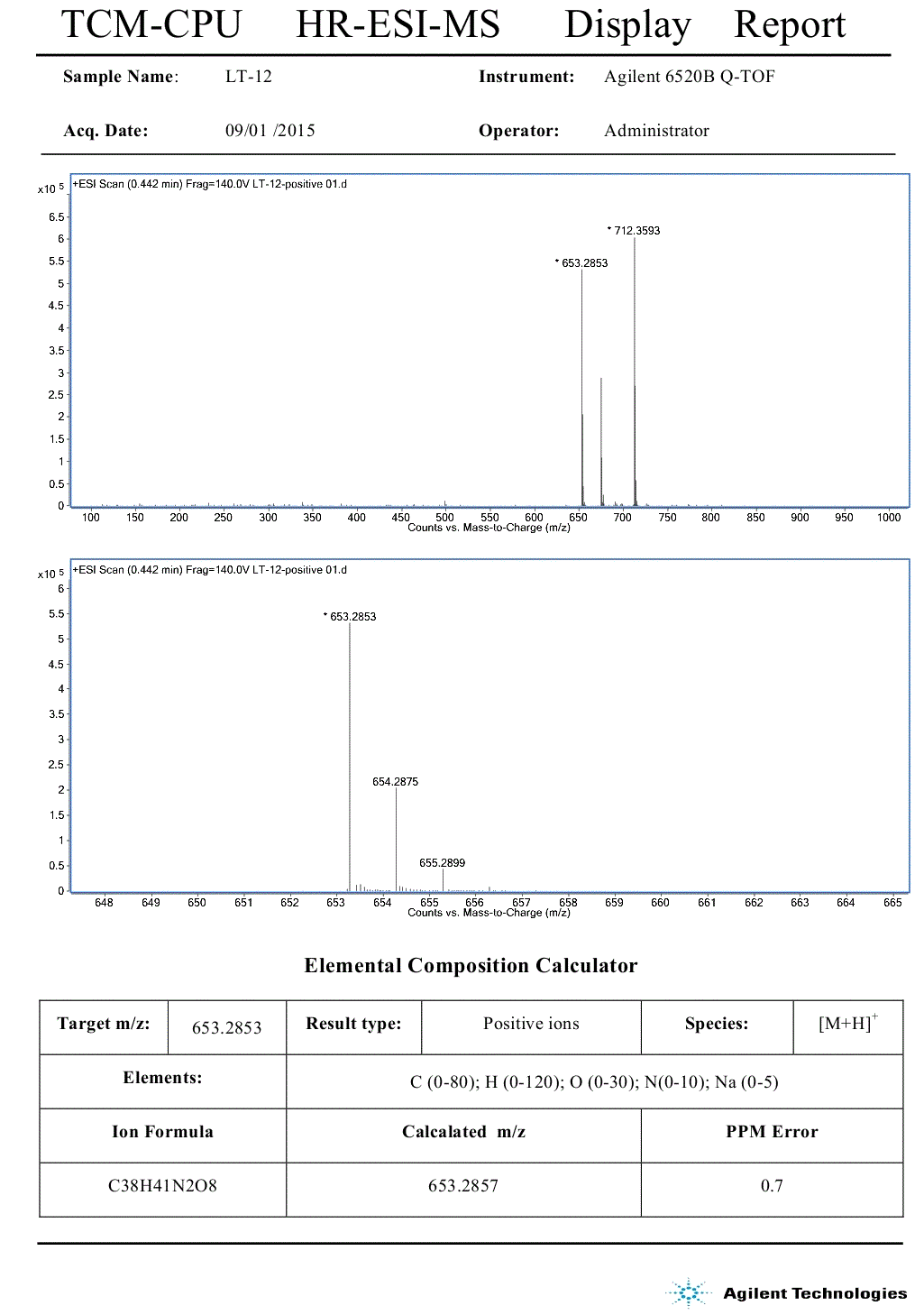


**Figure S3-1.** ^1^H NMR spectrum of compound **3** (CDCl_3_, 500 MHz).


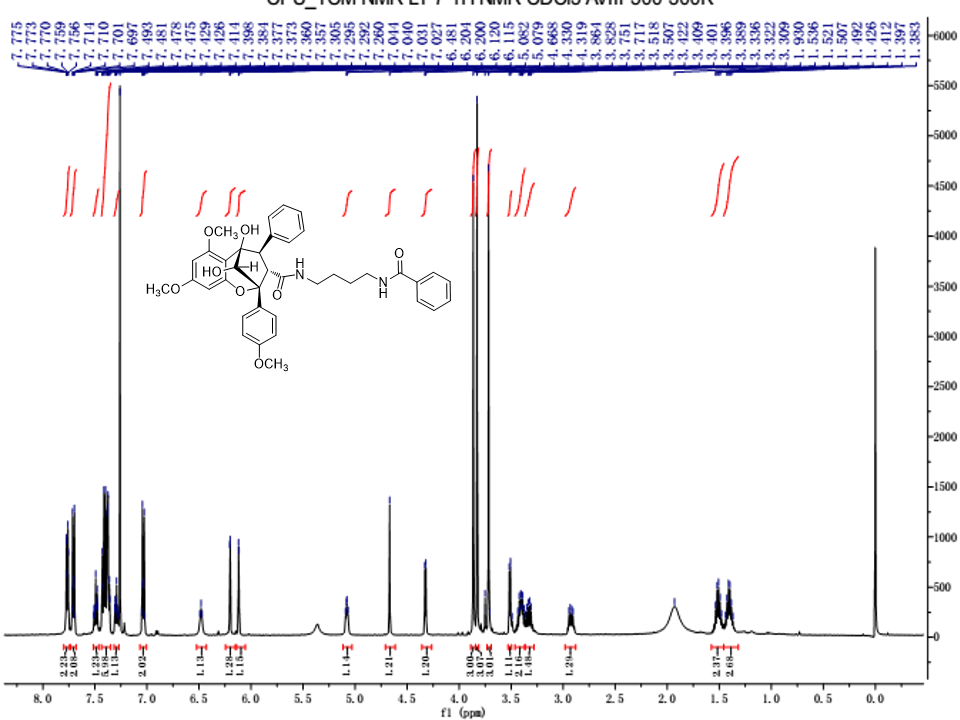


**Figure S3-2.** ^13^C NMR spectrum of compound **3** (CDCl_3_, 125 MHz).


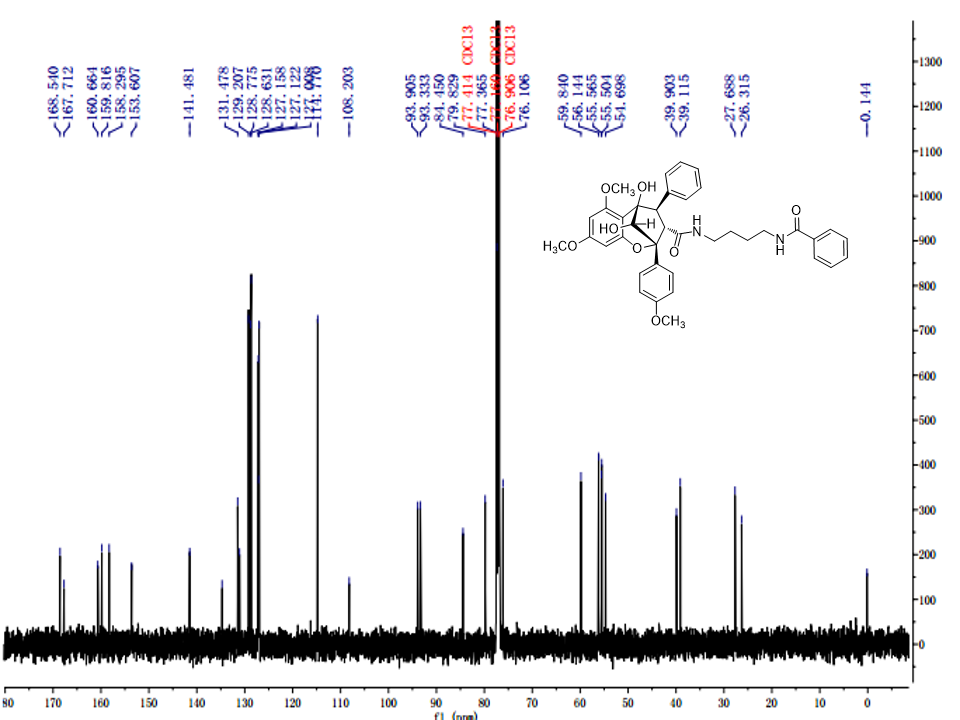


**Figure S3-3.** HSQC spectrum of compound **3** (CDCl_3_).

**
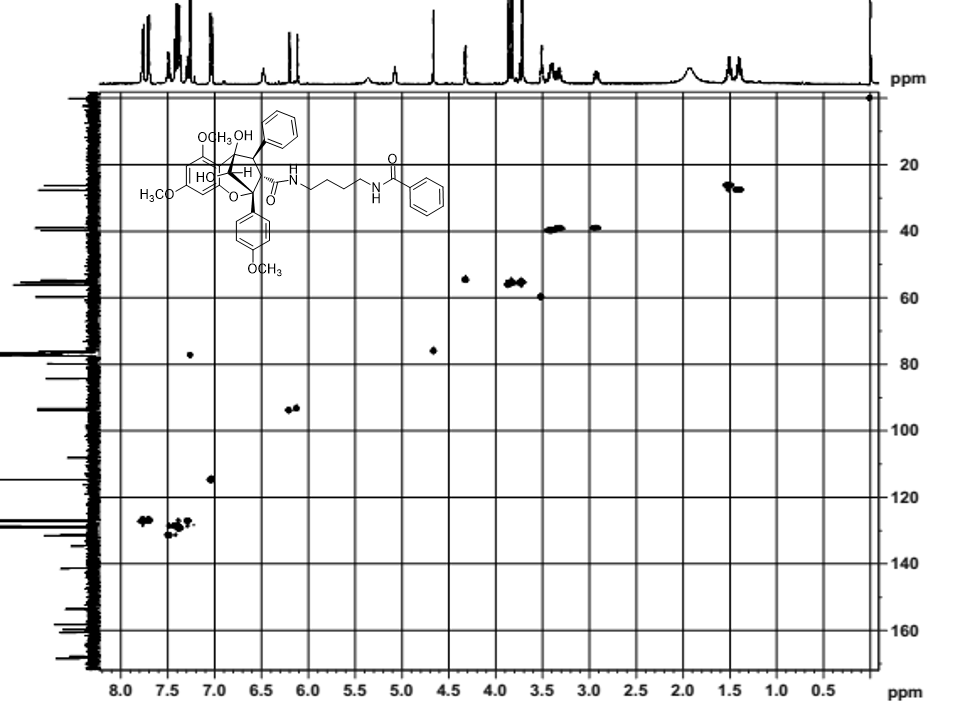
**

**Figure S3-4.** HMBC spectrum of compound **3** (CDCl_3_).


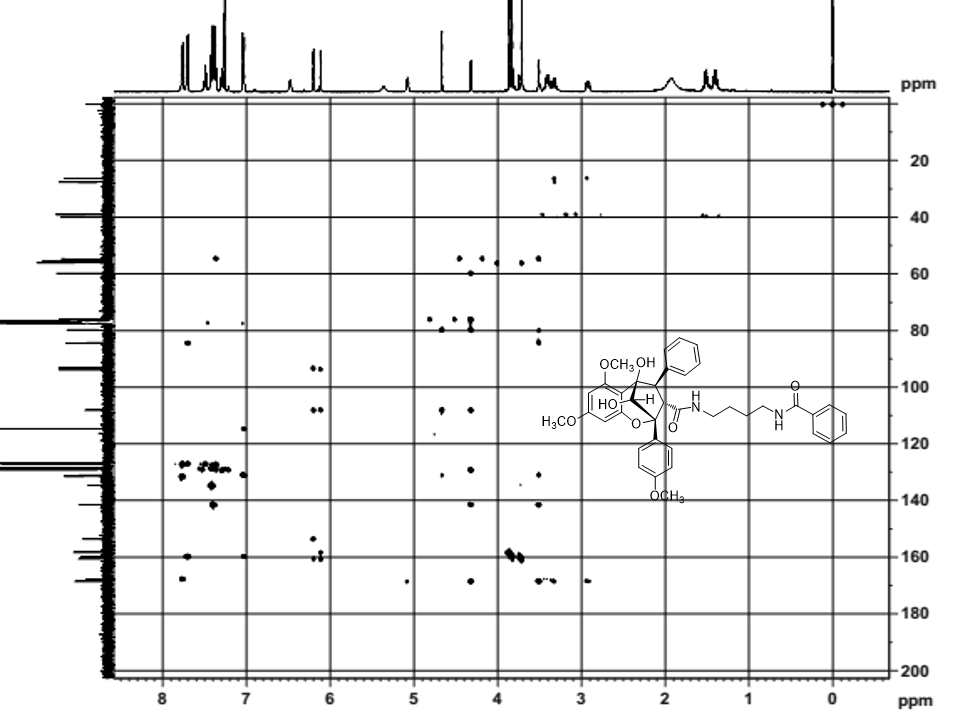


**Figure S3-5.** ROESY spectrum of compound **3** (CDCl_3_).


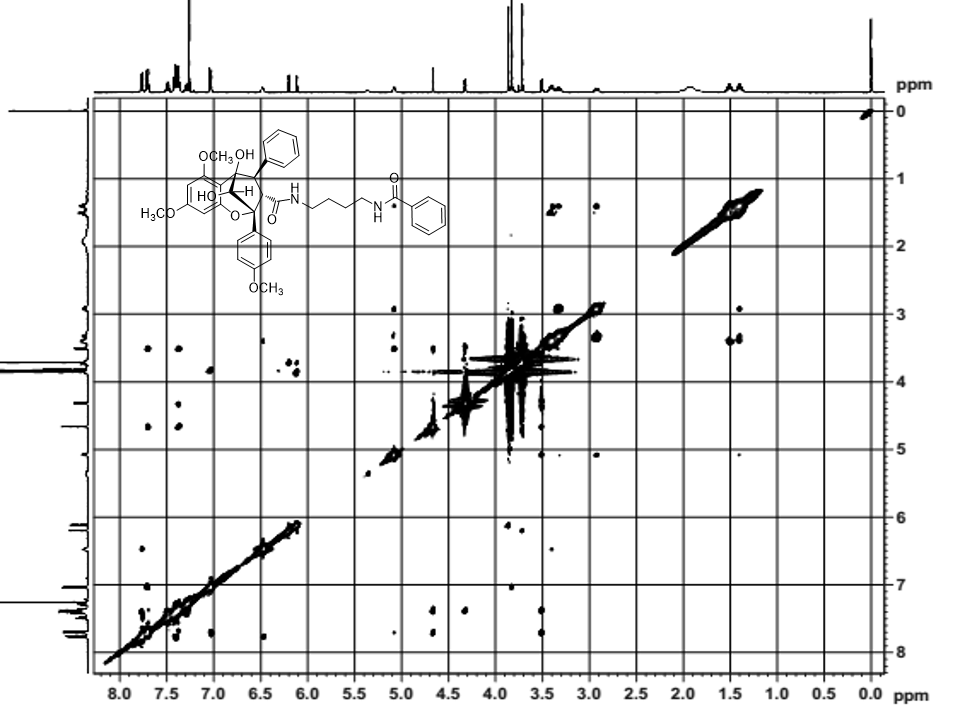


**Figure S3-6.** HRESIMS of compound **3**.


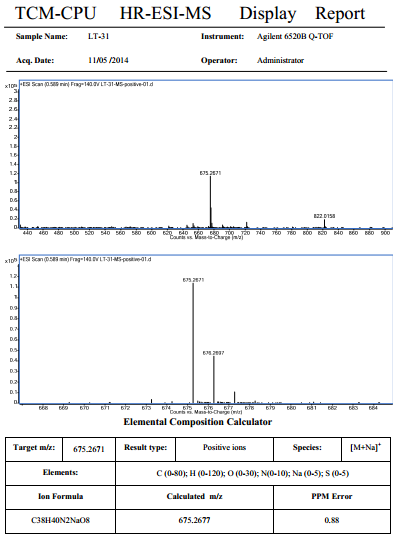

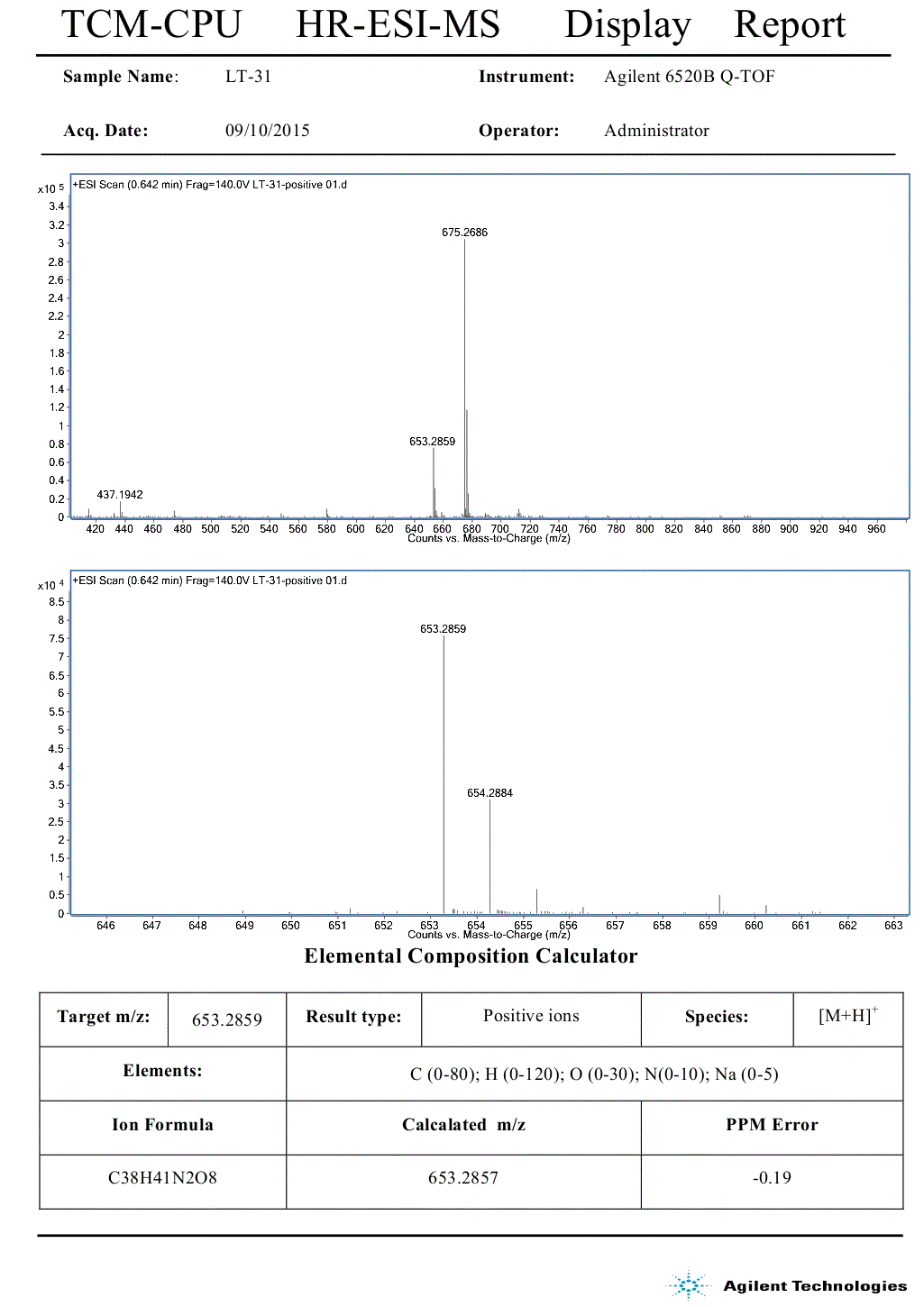


**Figure S4-1.** ^1^H NMR spectrum of compound **4** (CDCl_3_).


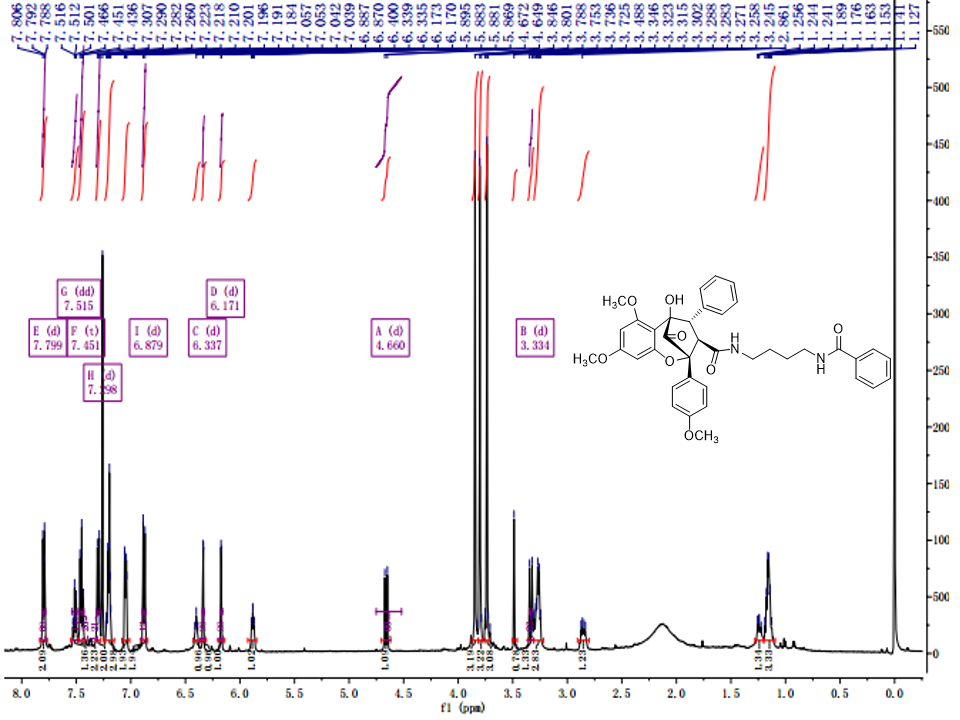


**Figure S4-2.** ^13^C NMR spectrum of compound **4** (CDCl_3_, 125 MHz).


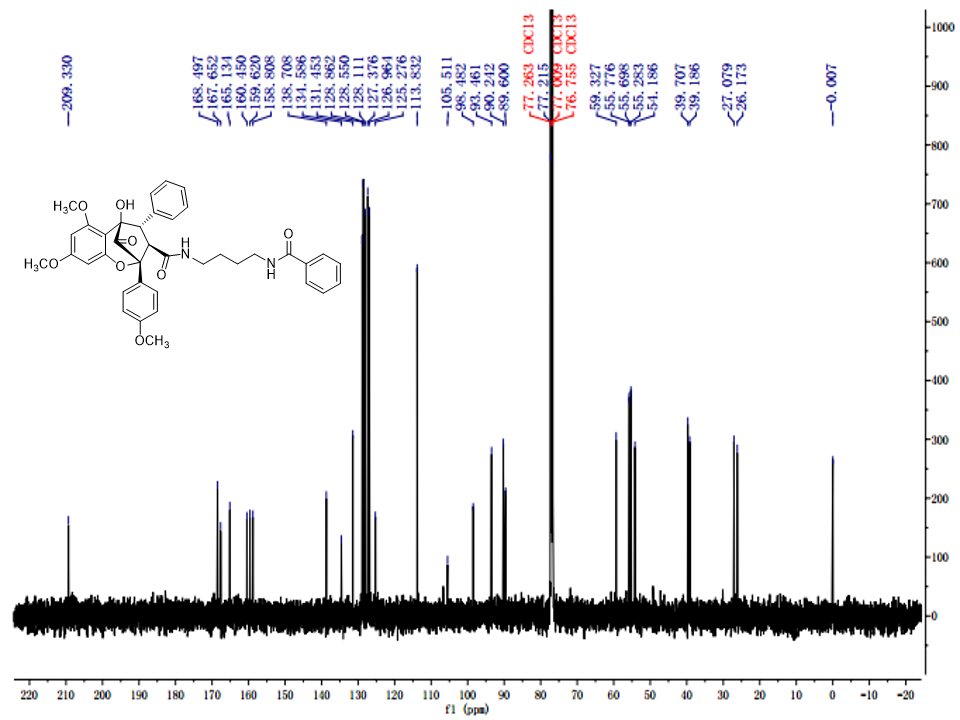


**Figure S4-3.** HSQC spectrum of compound **4** (CDCl_3_).

**
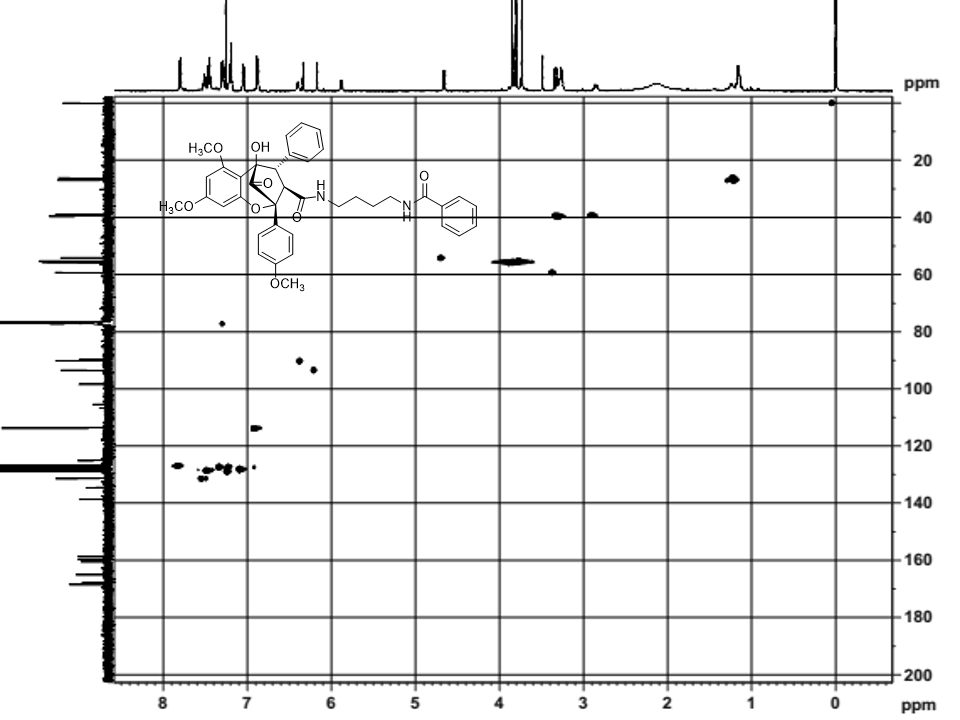
**

**Figure S4-4.** HMBC spectrum of compound **4** (CDCl_3_).


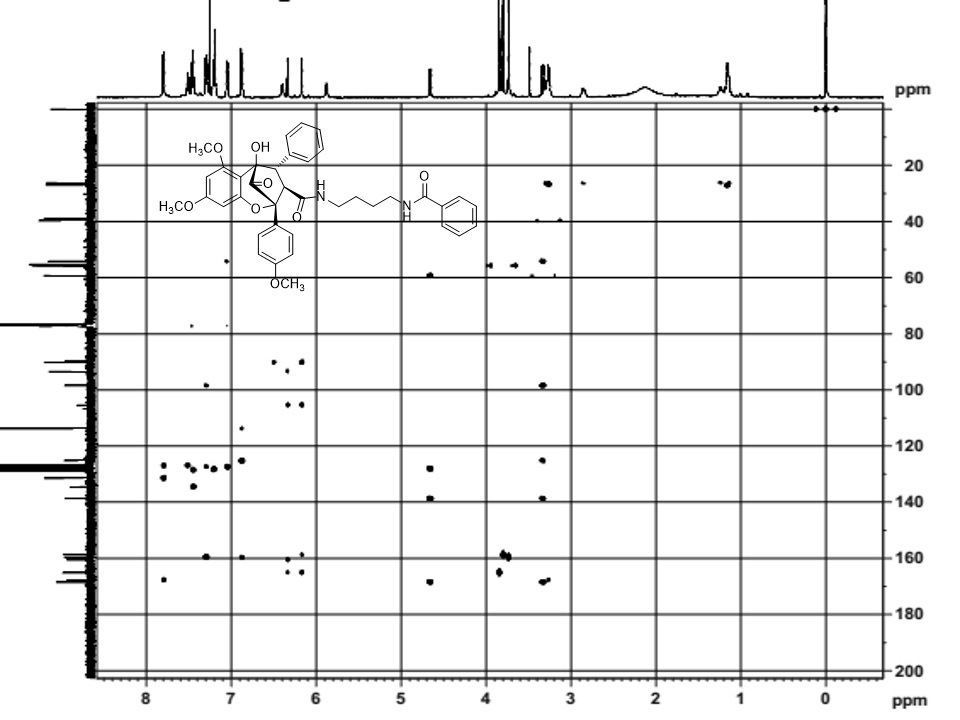


**Figure S4-5.** ROESY spectrum of compound **4** (CDCl_3_).


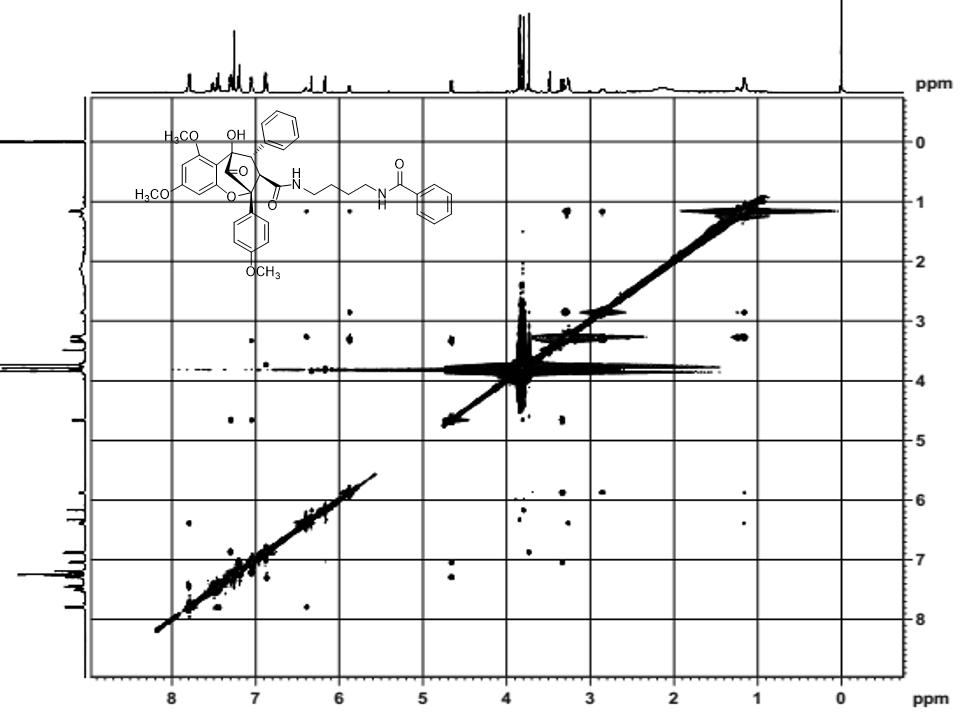


**Figure S4-6.** HRESIMS of compound **4**.


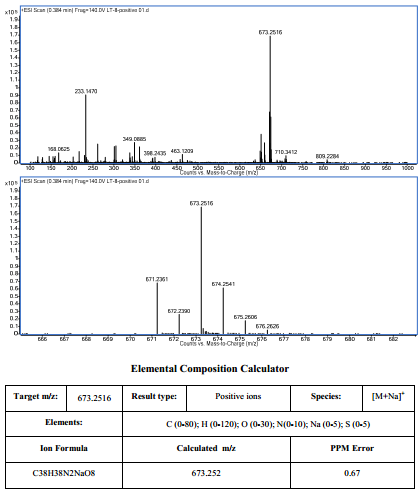

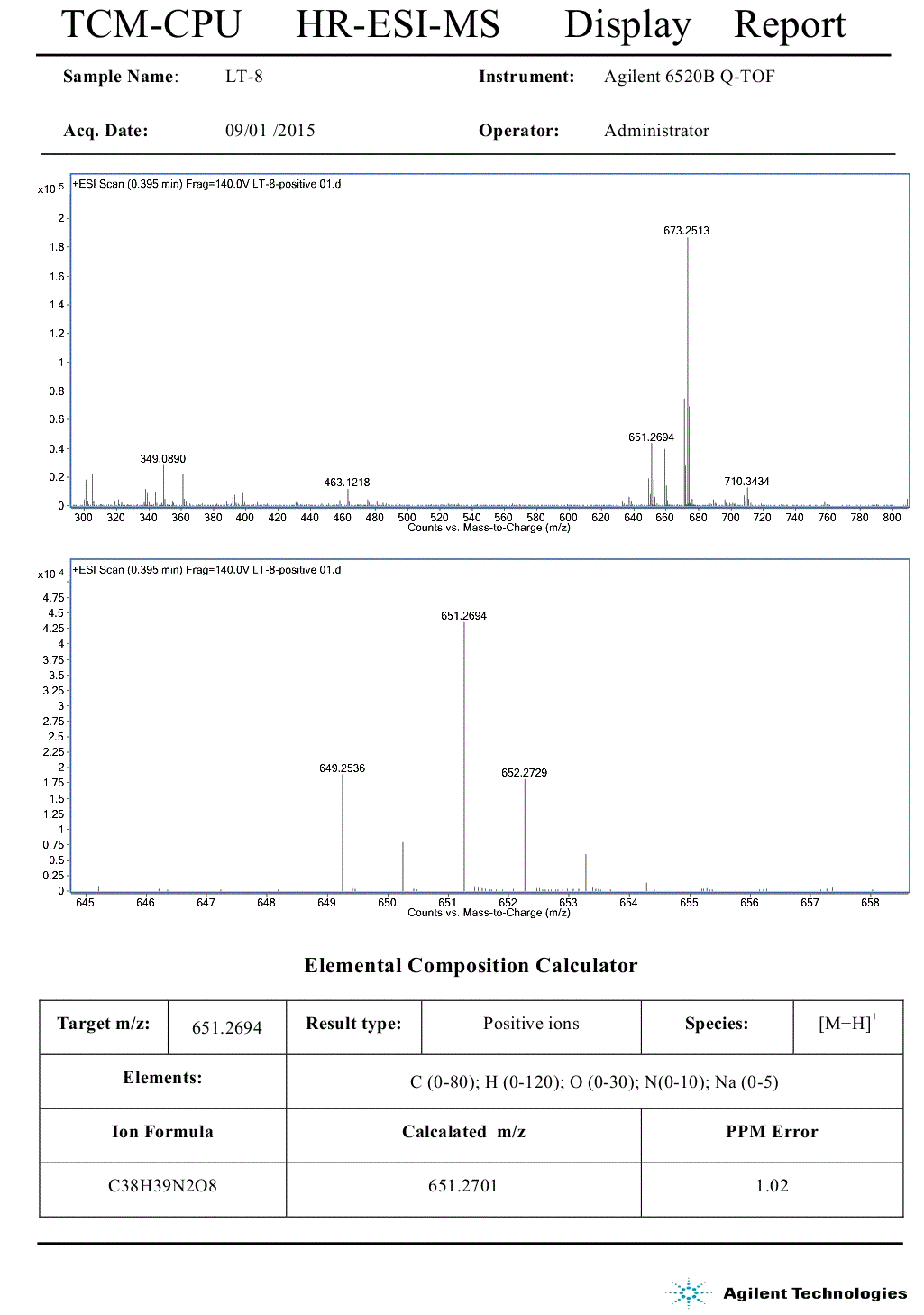


**Figure S5-1.** ^1^H NMR spectrum of compound **5** (CDCl_3_, 500 MHz).


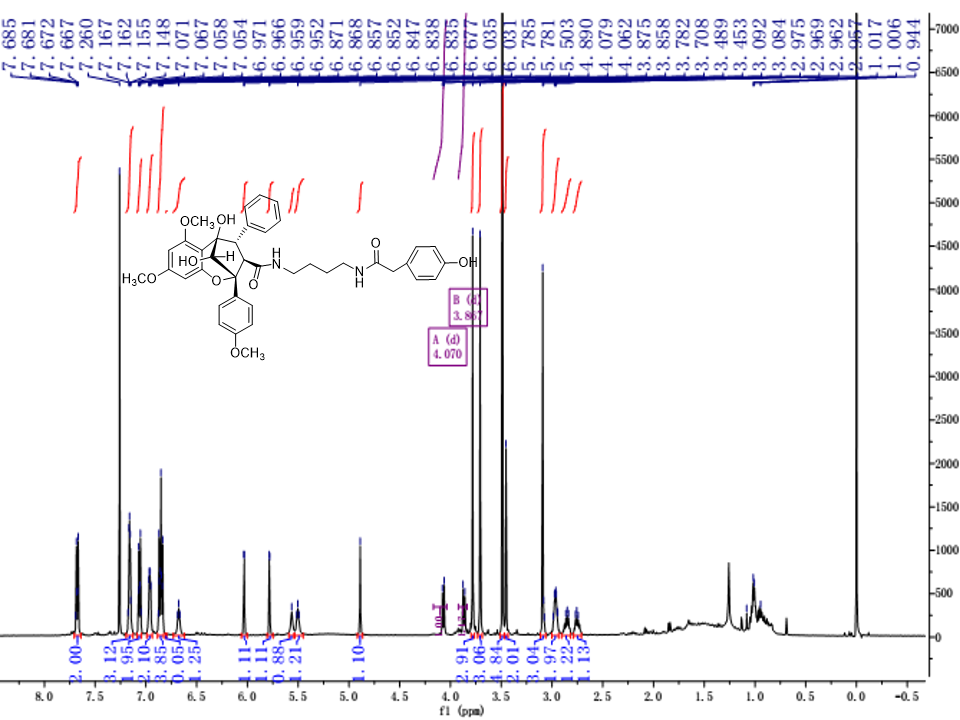


**Figure S5-2.** ^13^C NMR spectrum of compound **5** (CDCl_3_, 125 MHz).


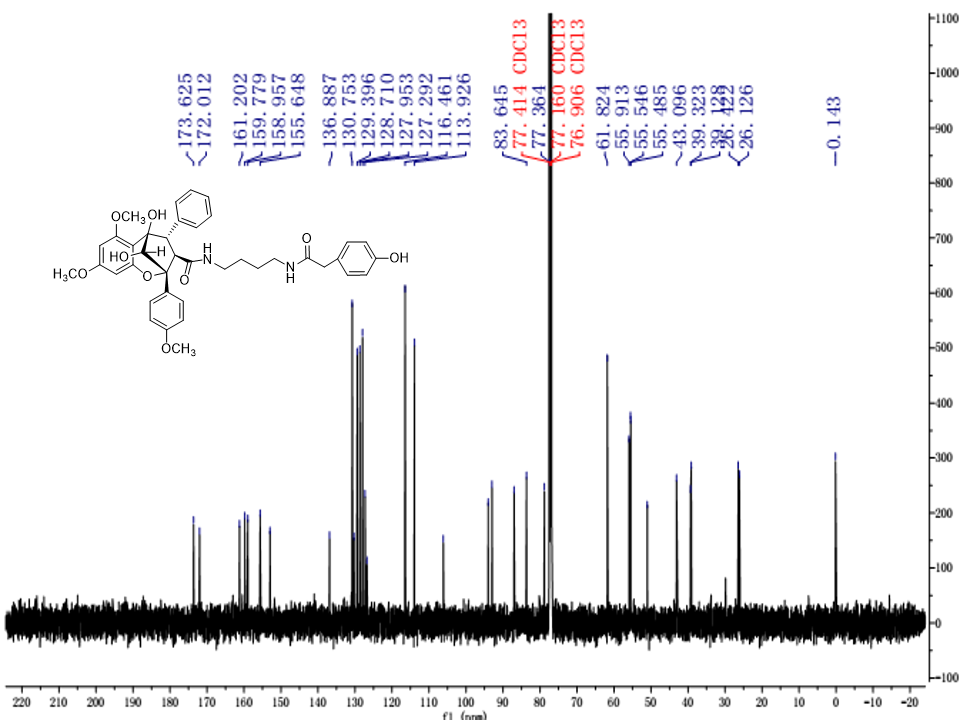


**Figure S5-3.** HSQC spectrum of compound **5** (CDCl_3_).

**
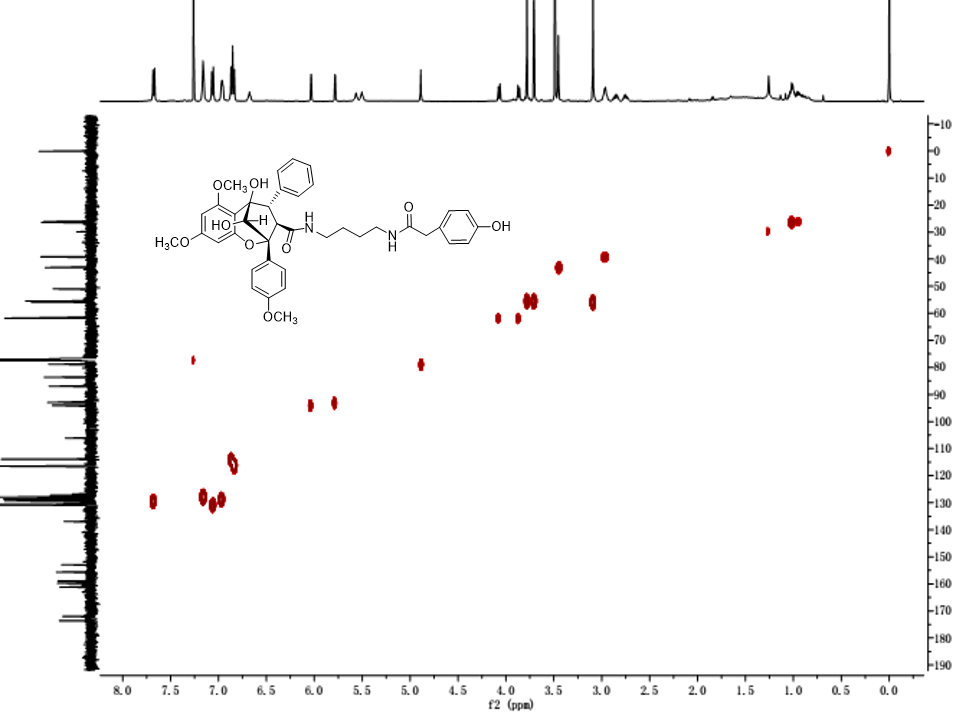
**

**Figure S5-4.** HMBC spectrum of compound **5** (CDCl_3_).


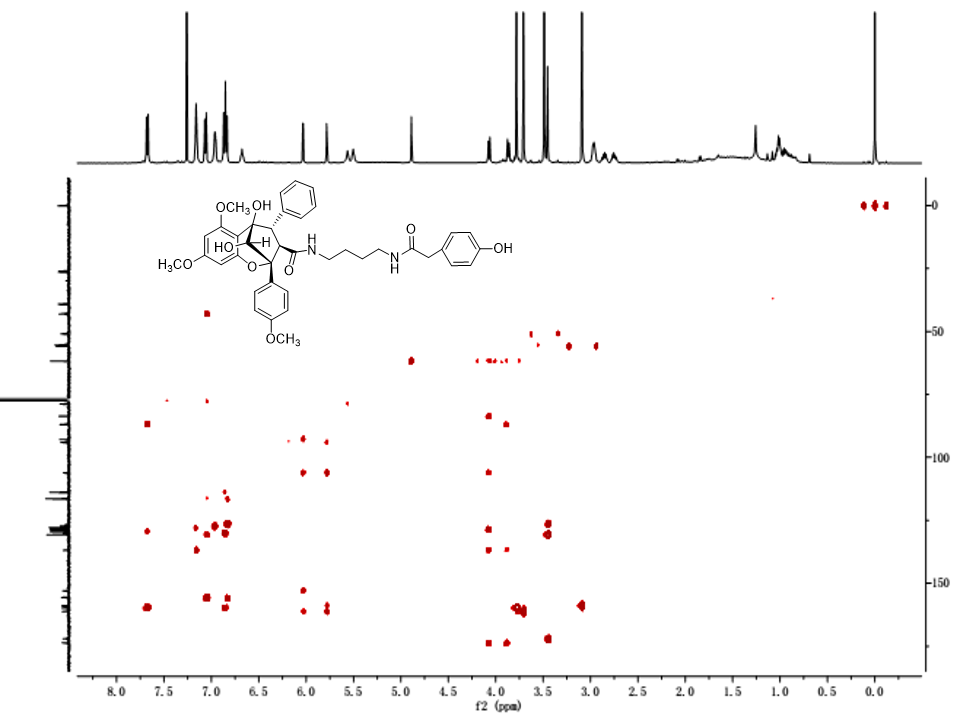


**Figure S5-5.** ROESY spectrum of compound **5** (CDCl_3_).


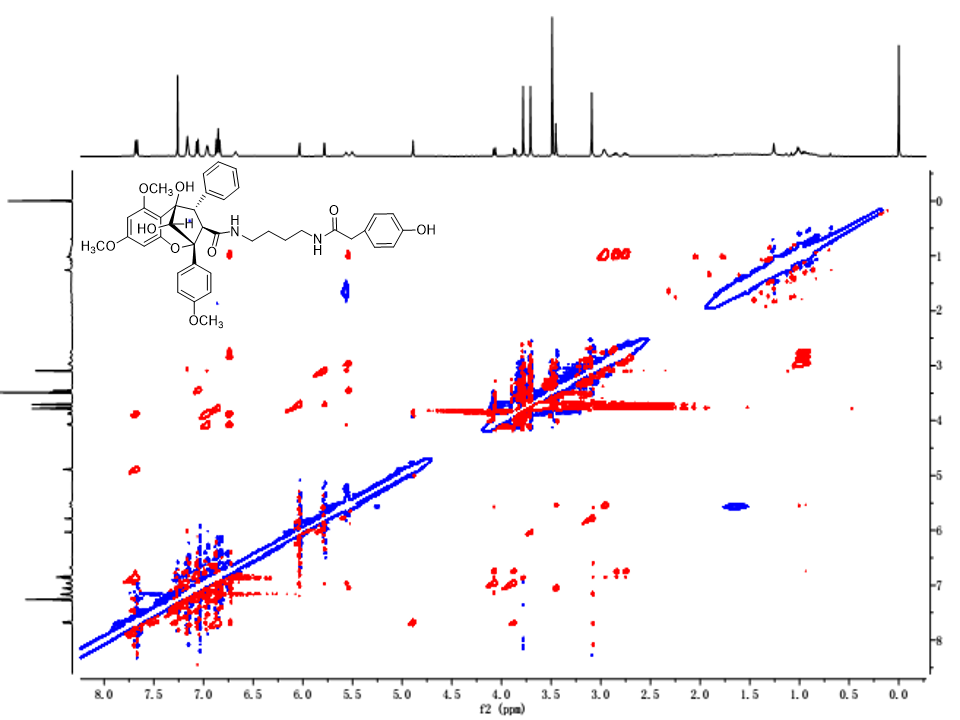


**Figure S5-6.** HRESIMS of compound **5**.


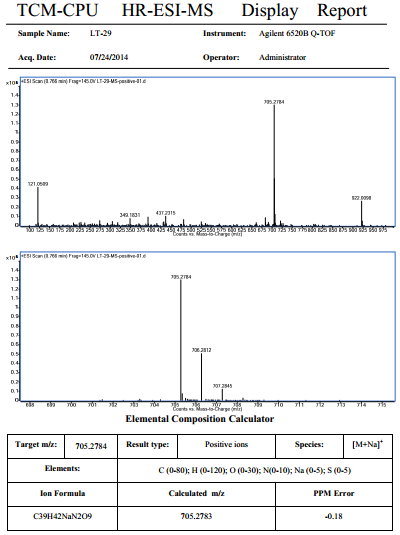


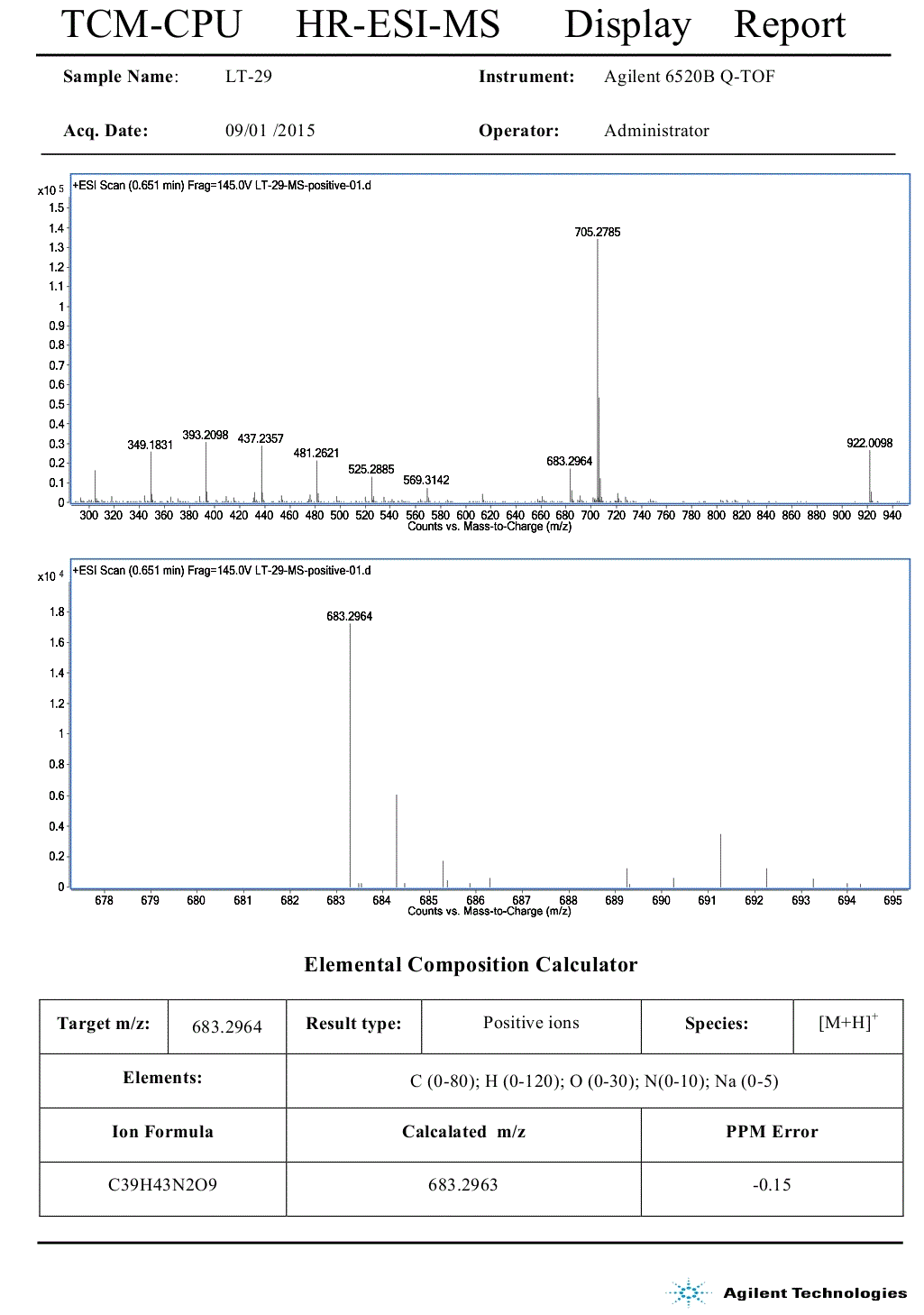


**Figure S6-1.** ^1^H NMR spectrum of compound **6** (CD_3_OD, 500 MHz).


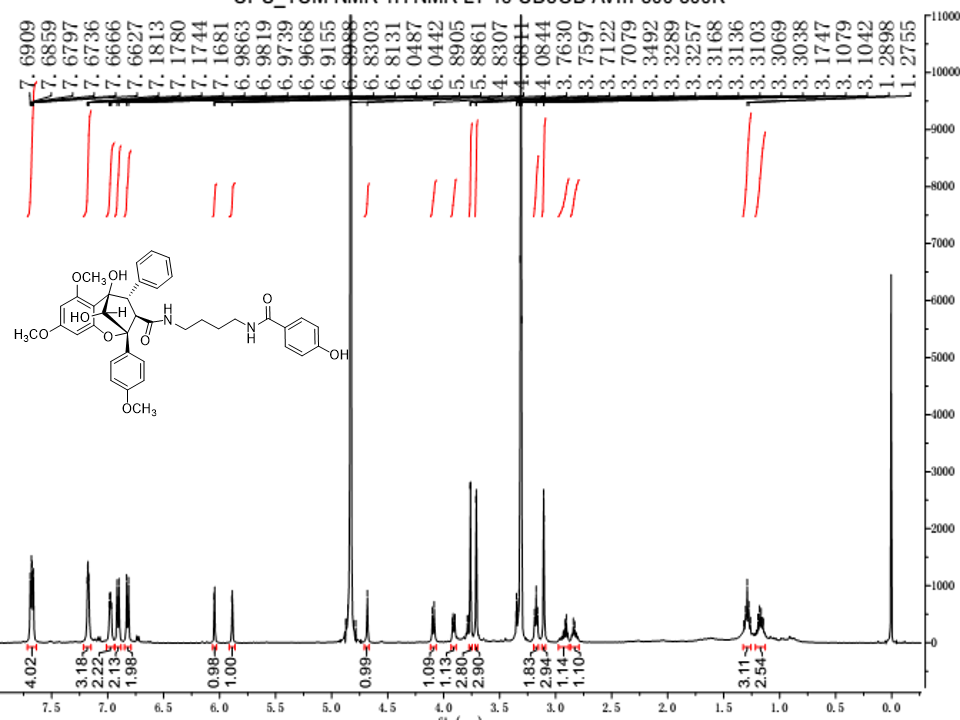


**Figure S6-2.** ^13^C NMR spectrum of compound **6** (CD_3_OD, 125 MHz).


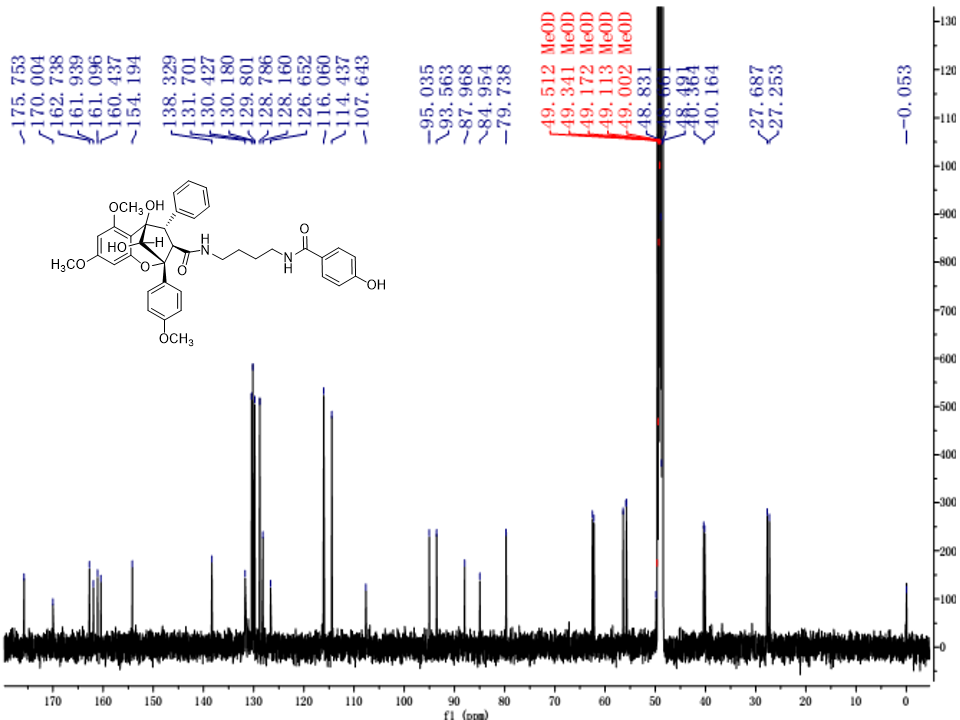


**Figure S6-3.** HSQC spectrum of compound **6** (CDCl_3_).

**
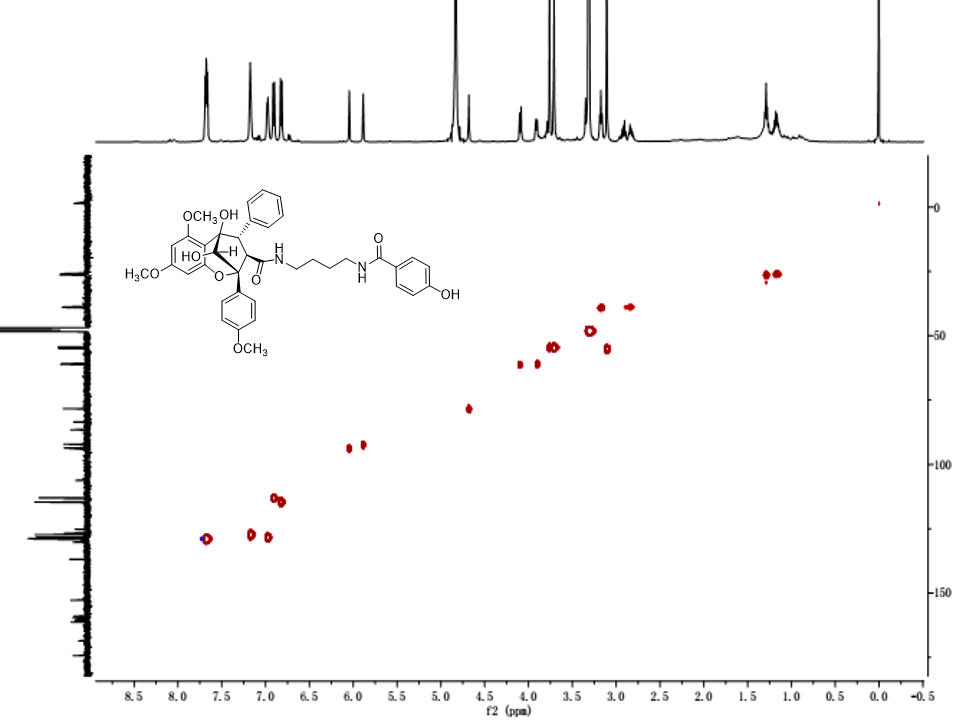
**

**Figure S6-4.** HMBC spectrum of compound **6** (CDCl_3_).


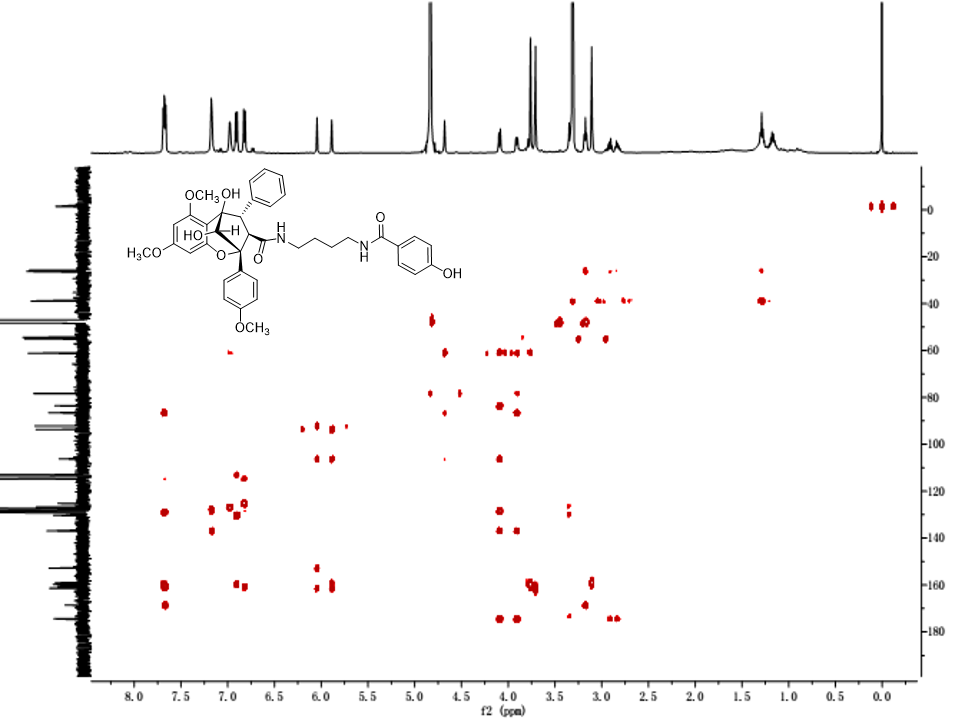


**Figure S6-5.** ROESY spectrum of compound **6** (CDCl_3_).


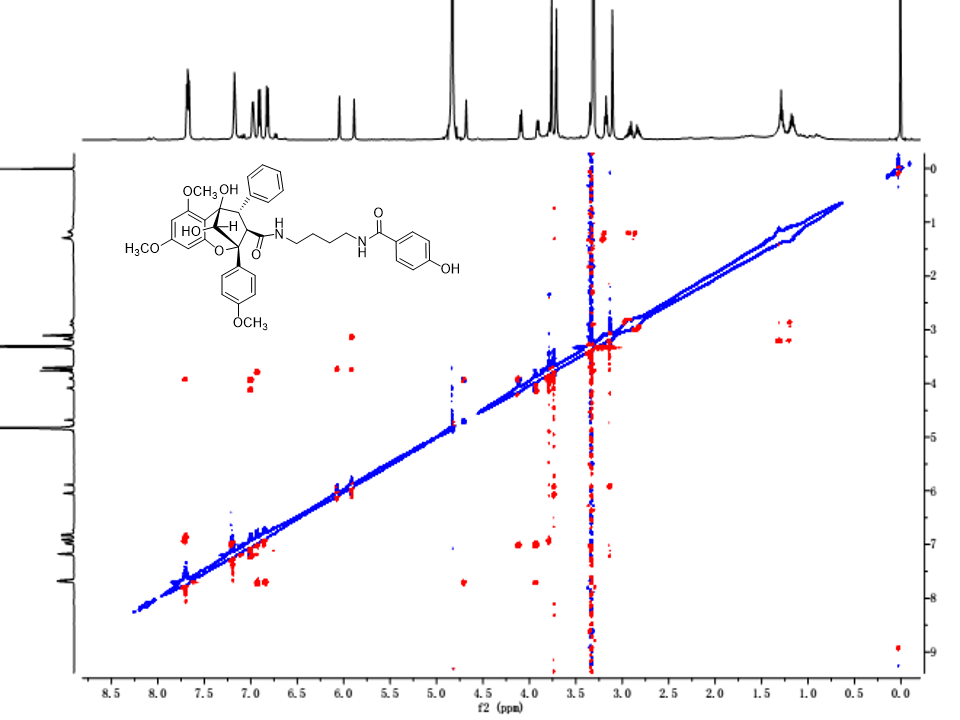


**Figure S6-6.** HRESIMS of compound **6**.


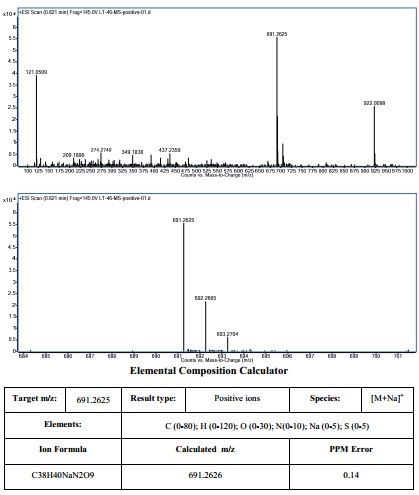


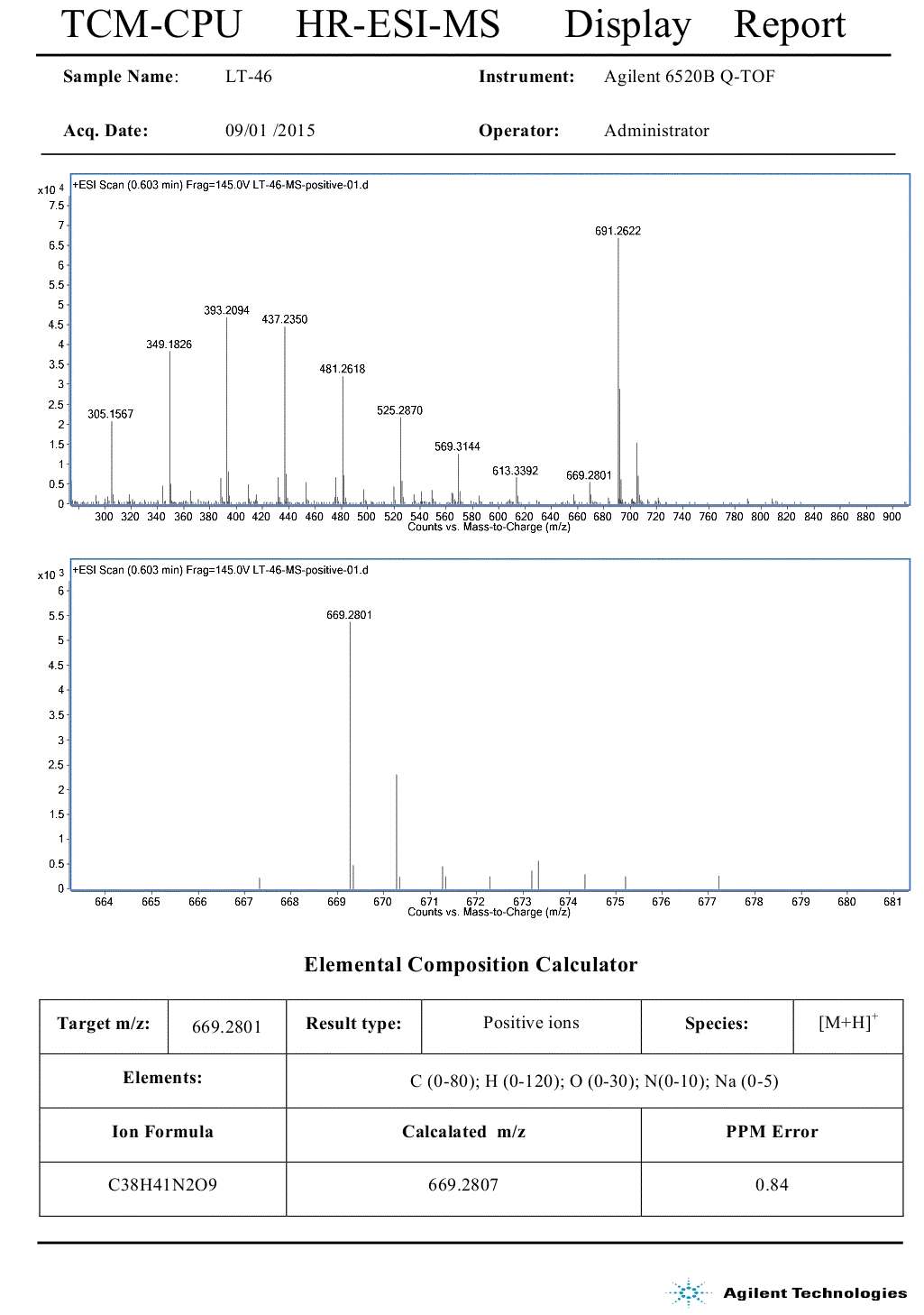


**Figure S7-1.** ^1^H NMR spectrum of compound **7** (CDCl_3_).


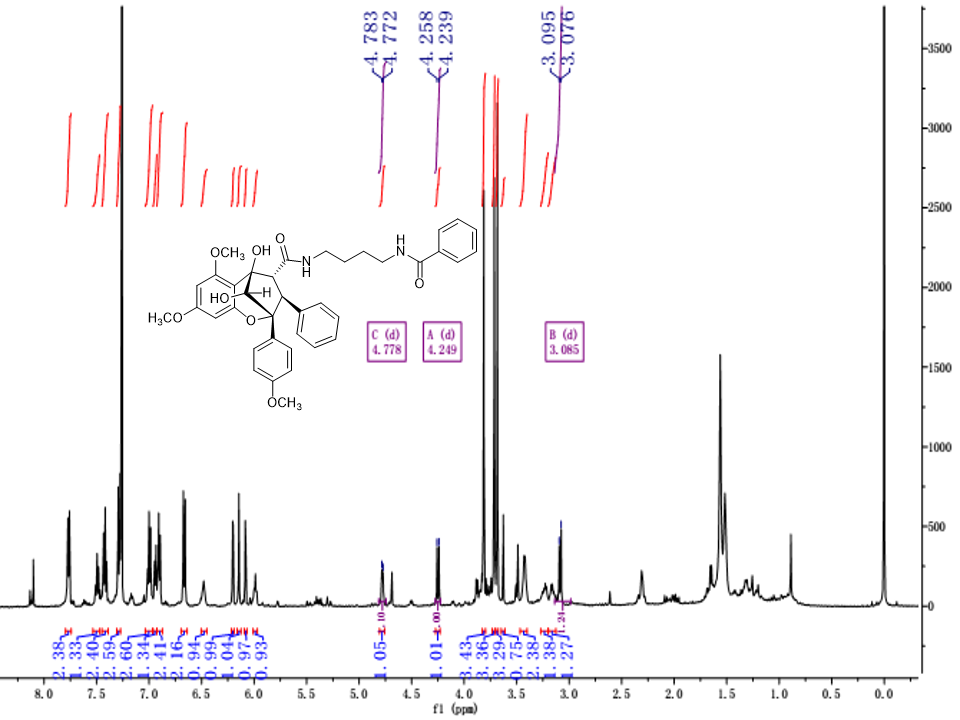


**Figure S7-2.** ^13^C NMR spectrum of compound **7** (CDCl_3_, 125 MHz).


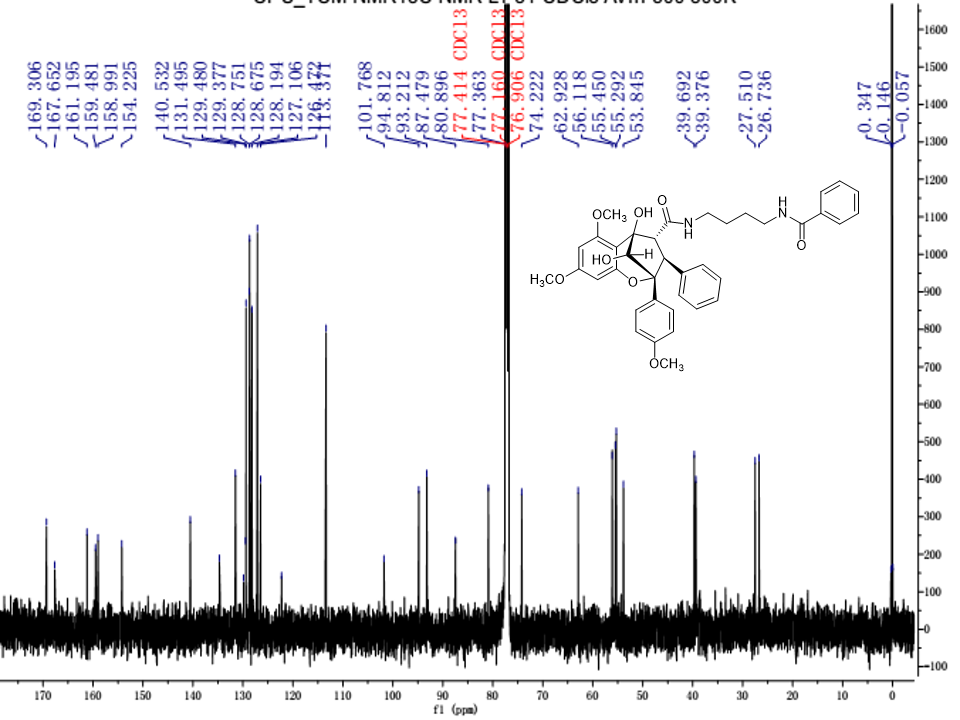


**Figure S7-3.** HSQC spectrum of compound **7** (CDCl_3_).

**
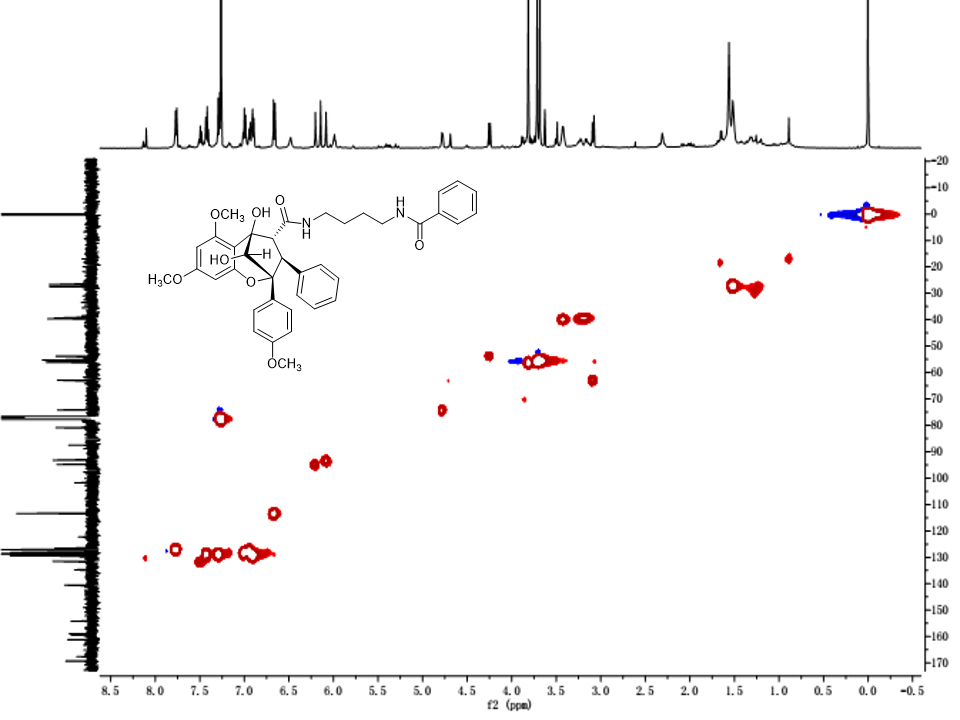
**

**Figure S7-4.** HMBC spectrum of compound **7** (CDCl_3_).


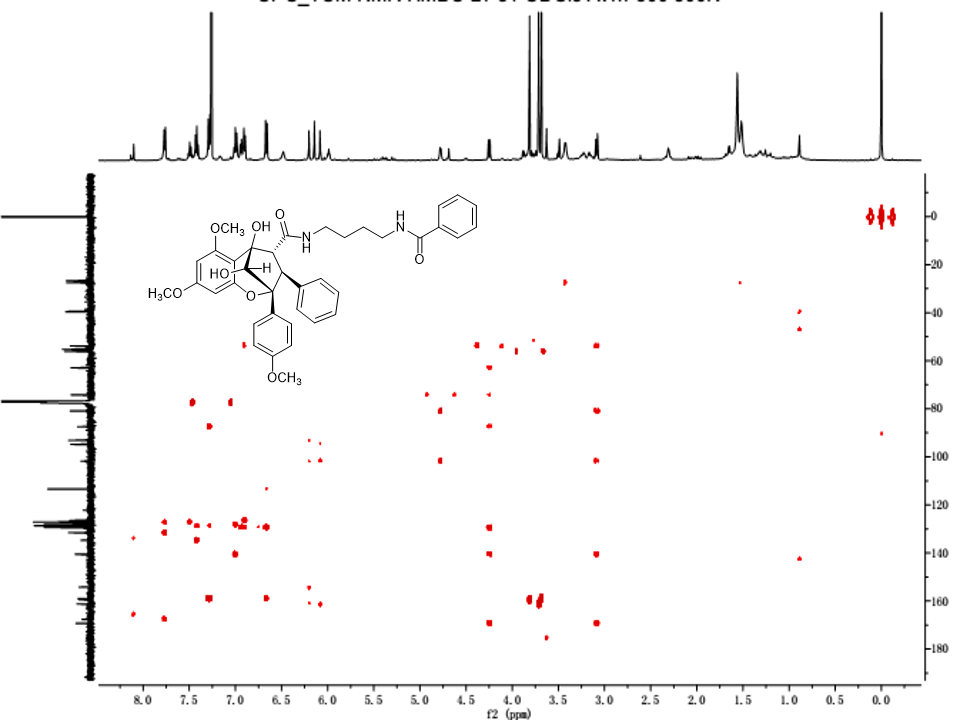


**Figure S7-5.** ROESY spectrum of compound **7** (CDCl_3_).


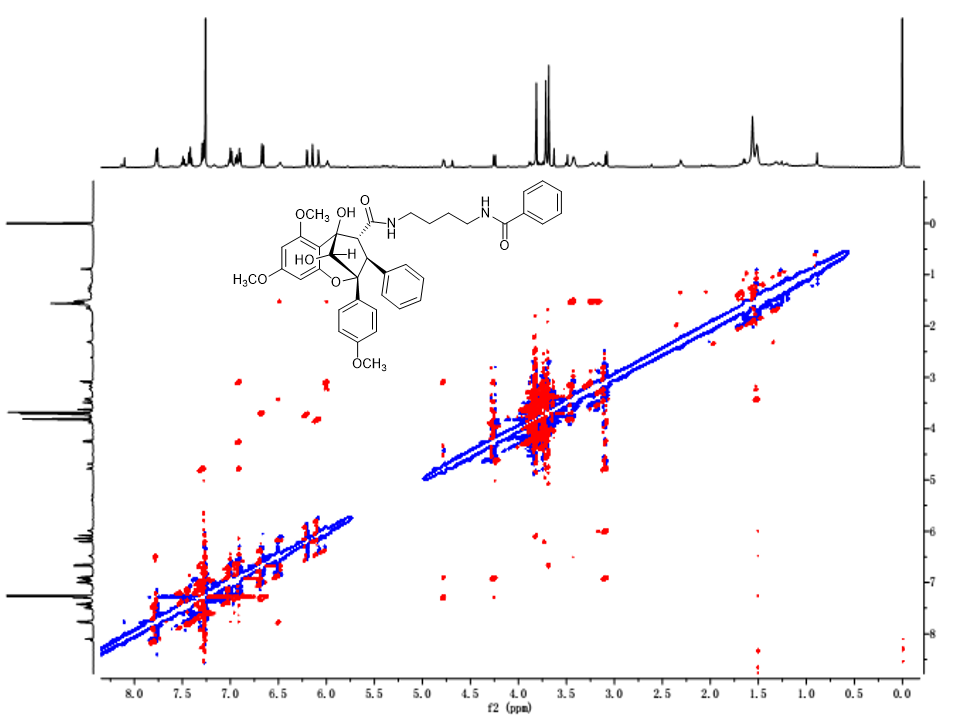


**Figure S7-6.** HRESIMS of compound **7**.


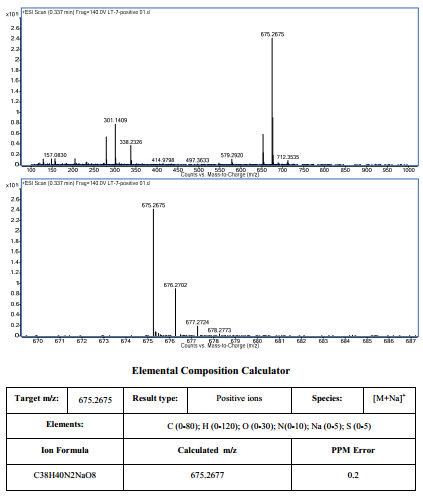


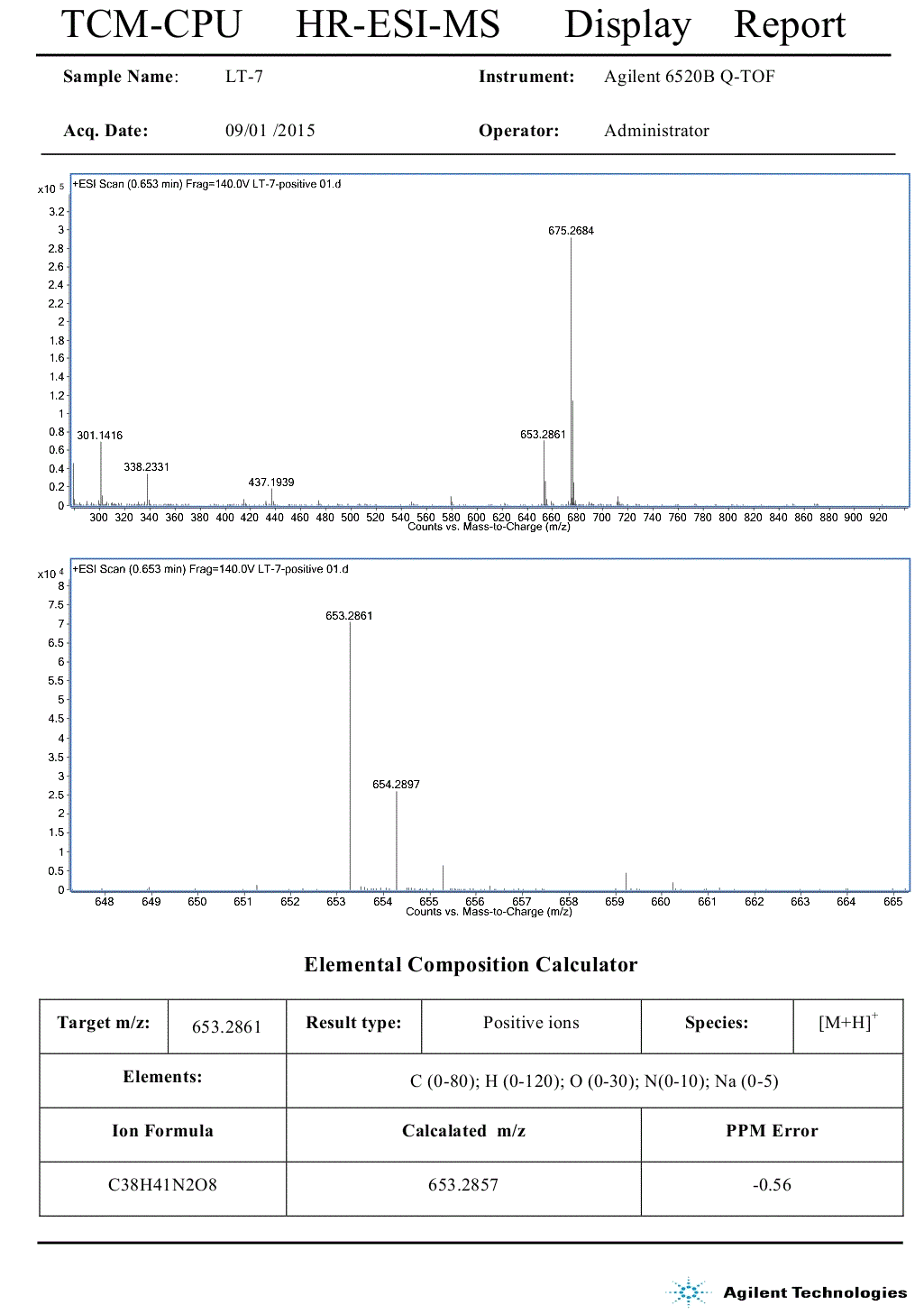


**Figure S8-1.** ^1^H NMR spectrum of compound **8** (CDCl_3_).


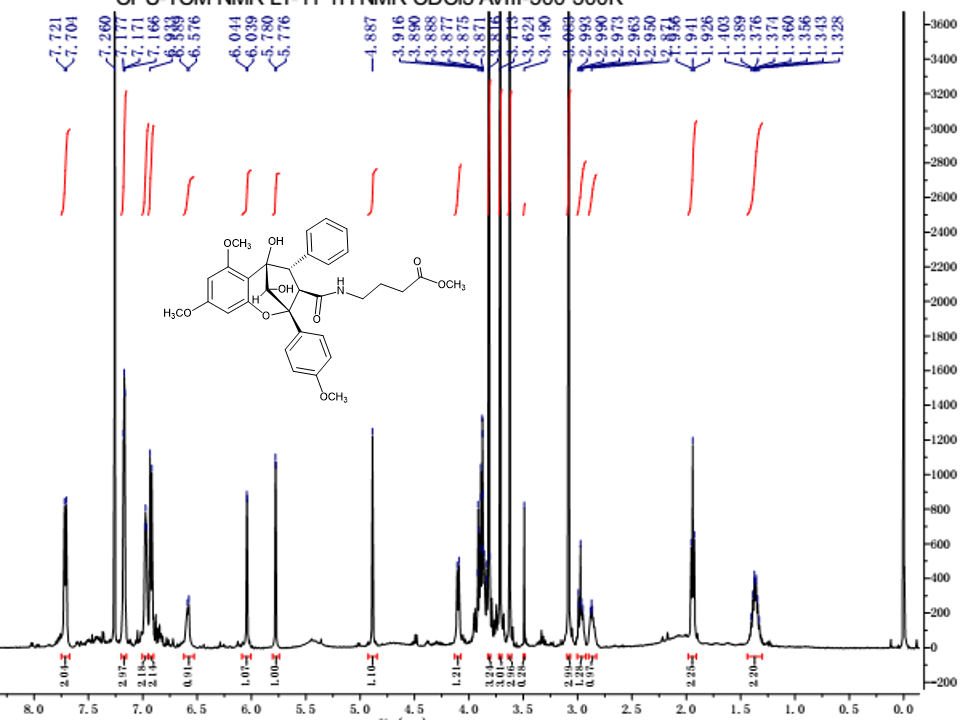


**Figure S8-2.** ^13^C NMR spectrum of compound **8** (CDCl_3_, 125 MHz).


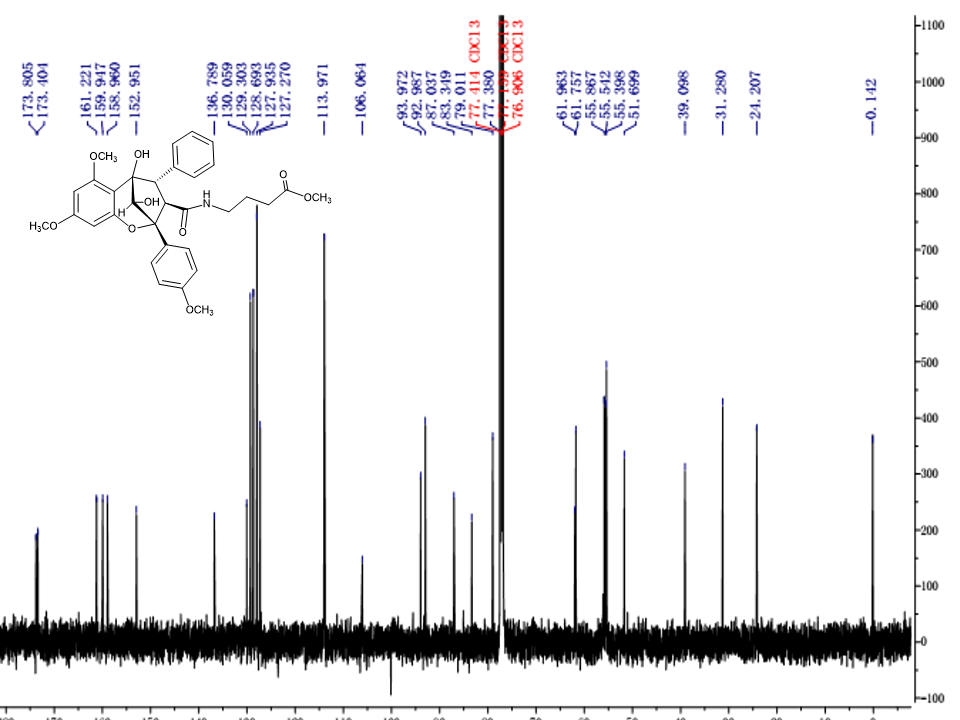


**Figure S8-3.** HSQC spectrum of compound **8** (CDCl_3_).

**
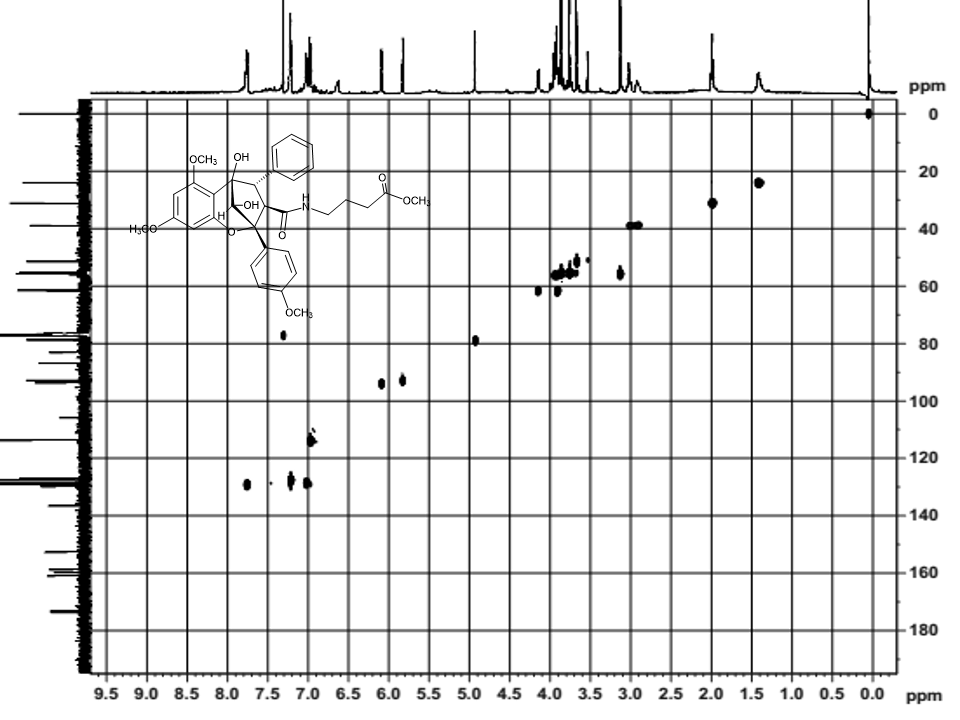
**

**Figure S8-4.** HMBC spectrum of compound **8** (CDCl_3_).


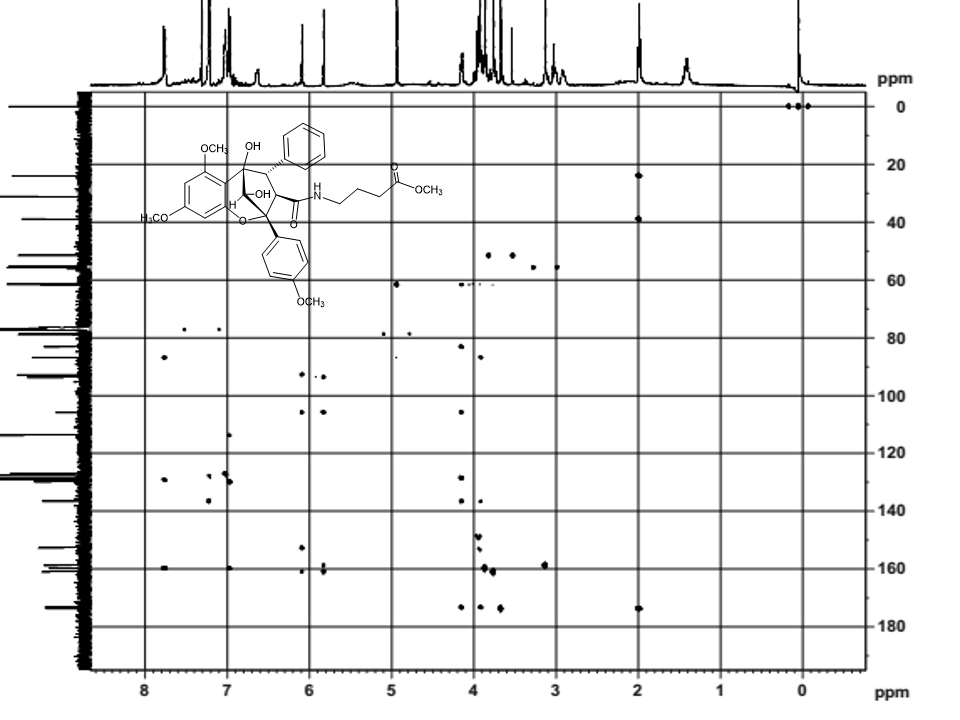


**Figure S8-5.** ROESY spectrum of compound **8** (CDCl_3_).


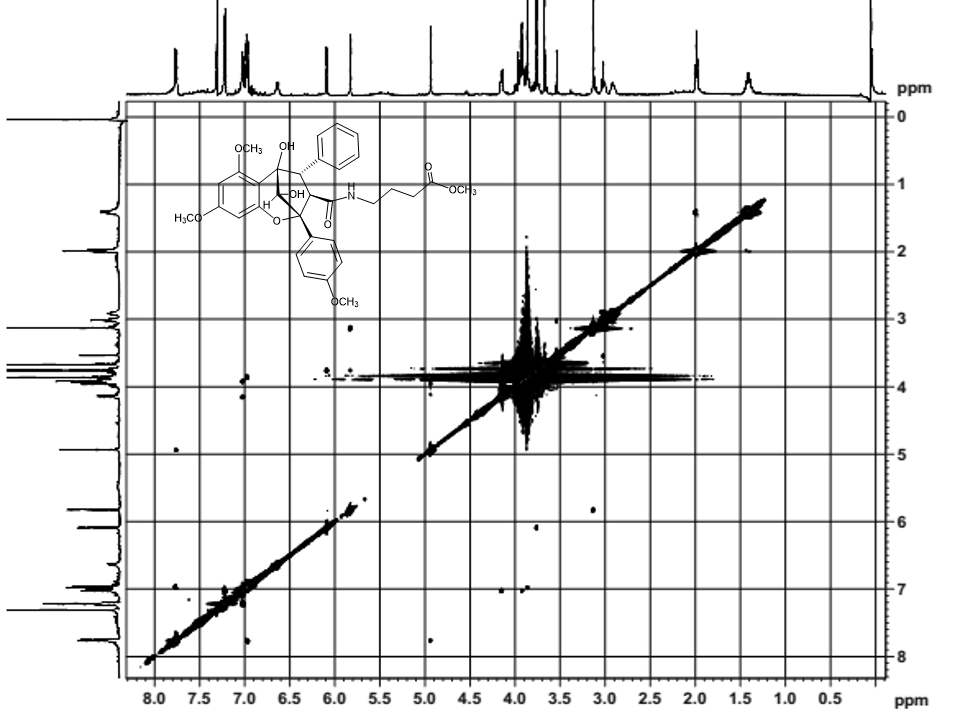


**Figure S8-6.** HRESIMS of compound **8**.


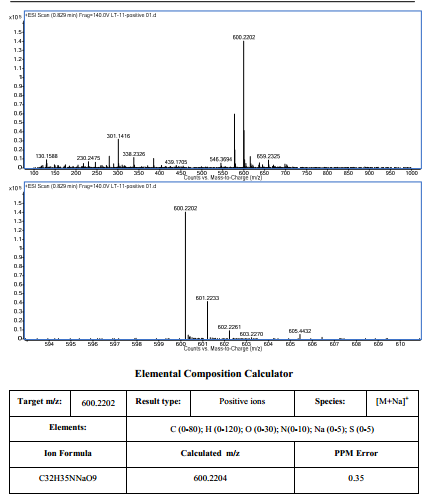


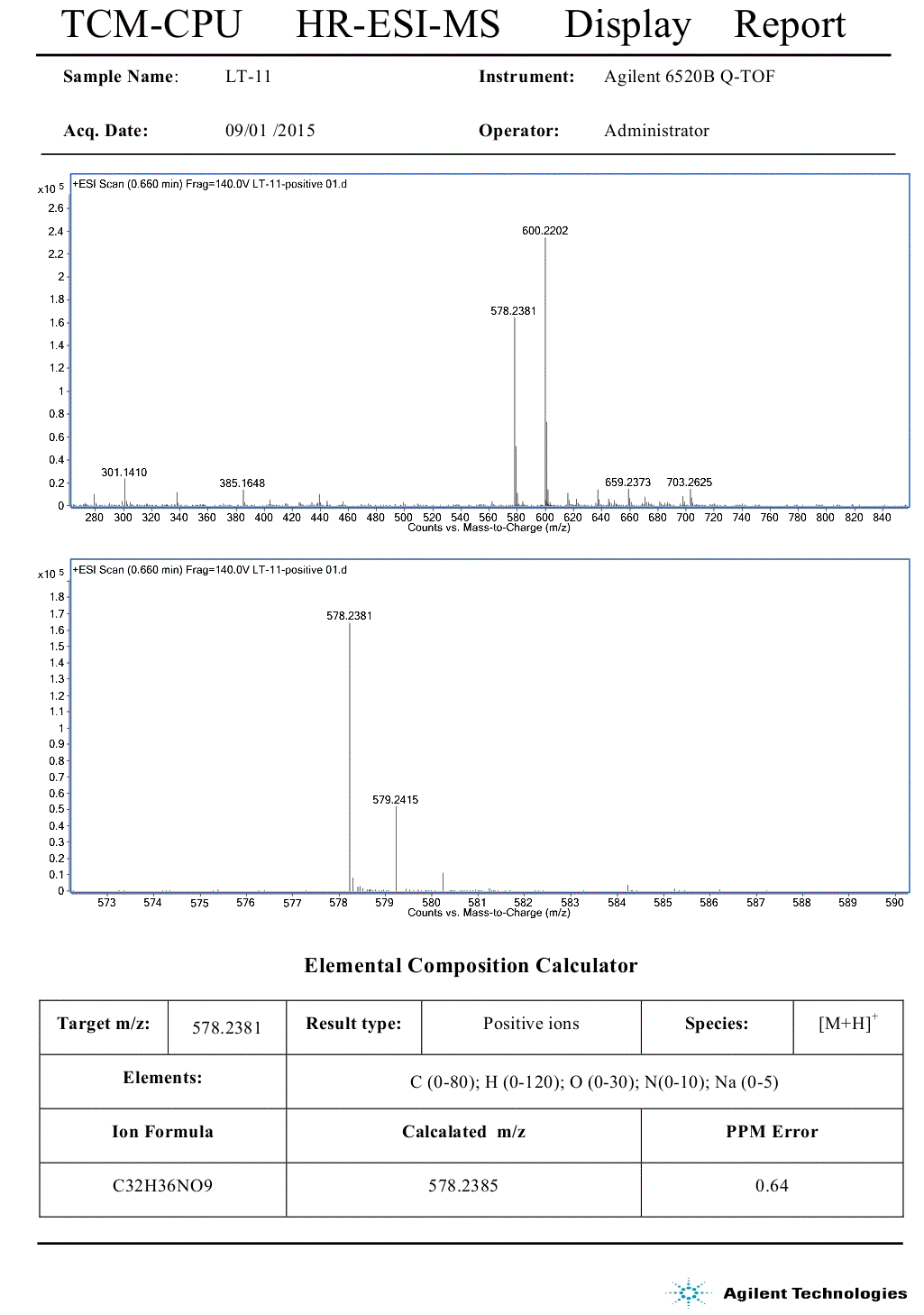


**Figure S9-1.** ^1^H NMR spectrum of compound **9** (CDCl_3_).


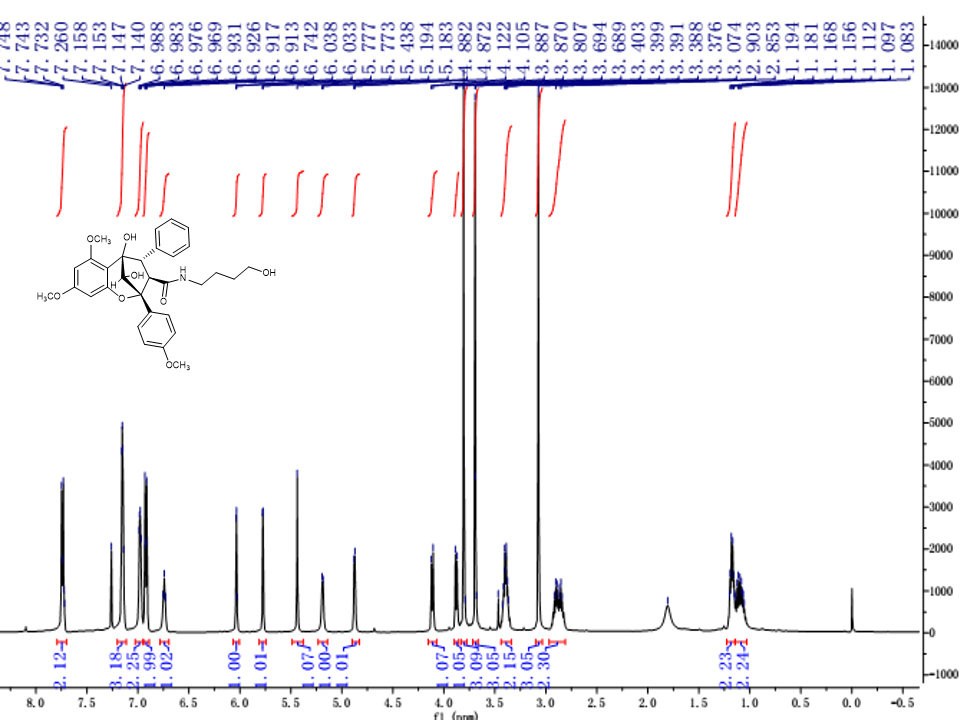


**Figure S9-2.** ^13^C NMR spectrum of compound **9** (CDCl_3_).


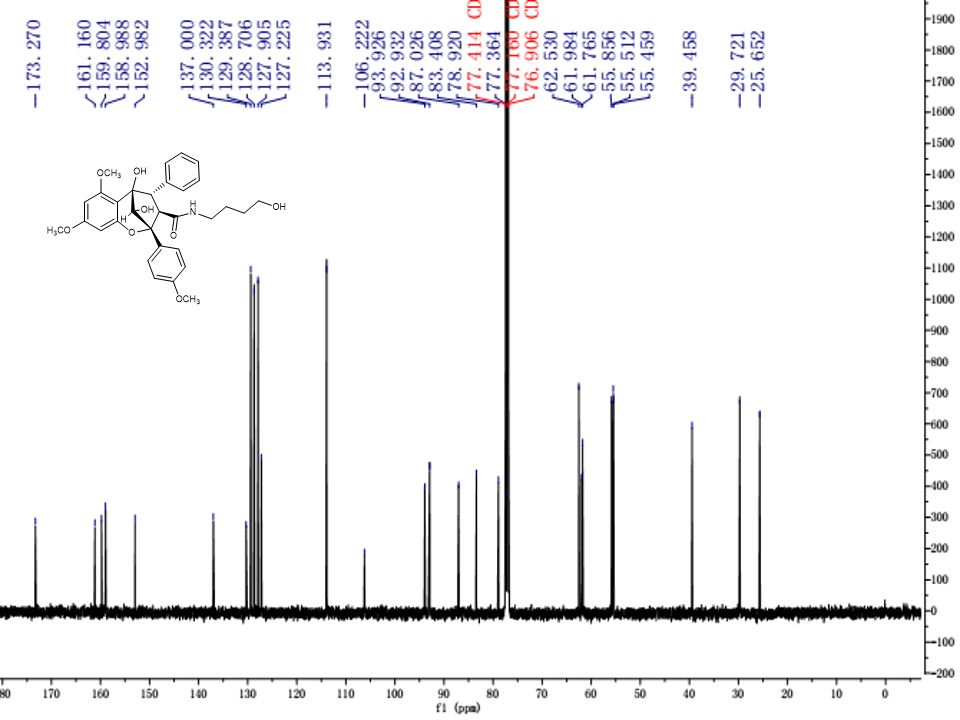


**Figure S9-3.** HSQC spectrum of compound **9** (CDCl_3_).

**
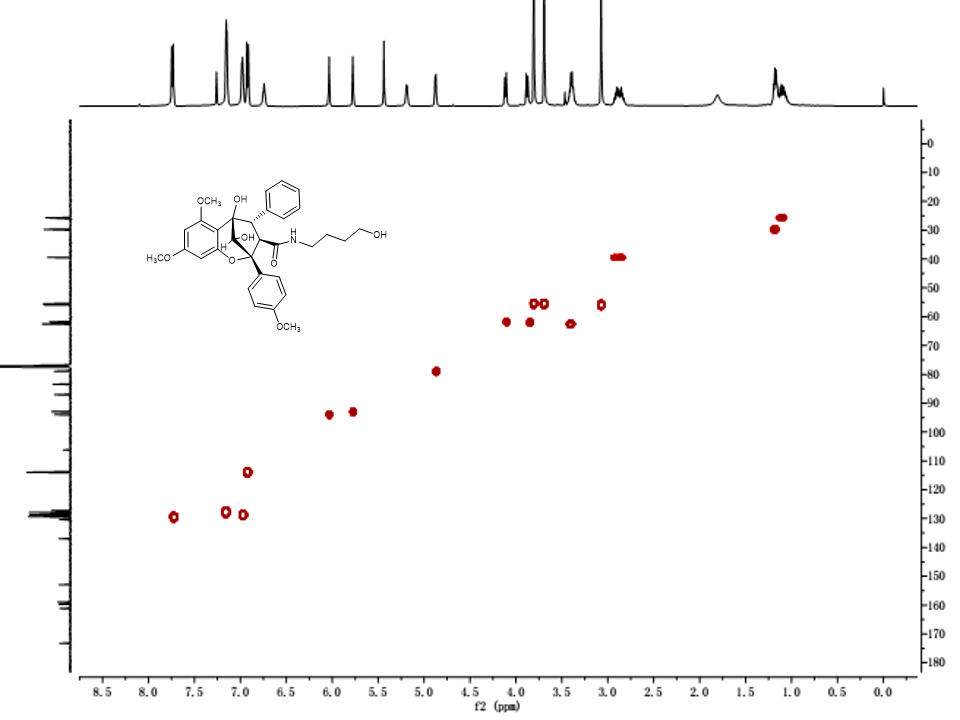
**

**Figure S9-4.** HMBC spectrum of compound **9** (CDCl_3_).


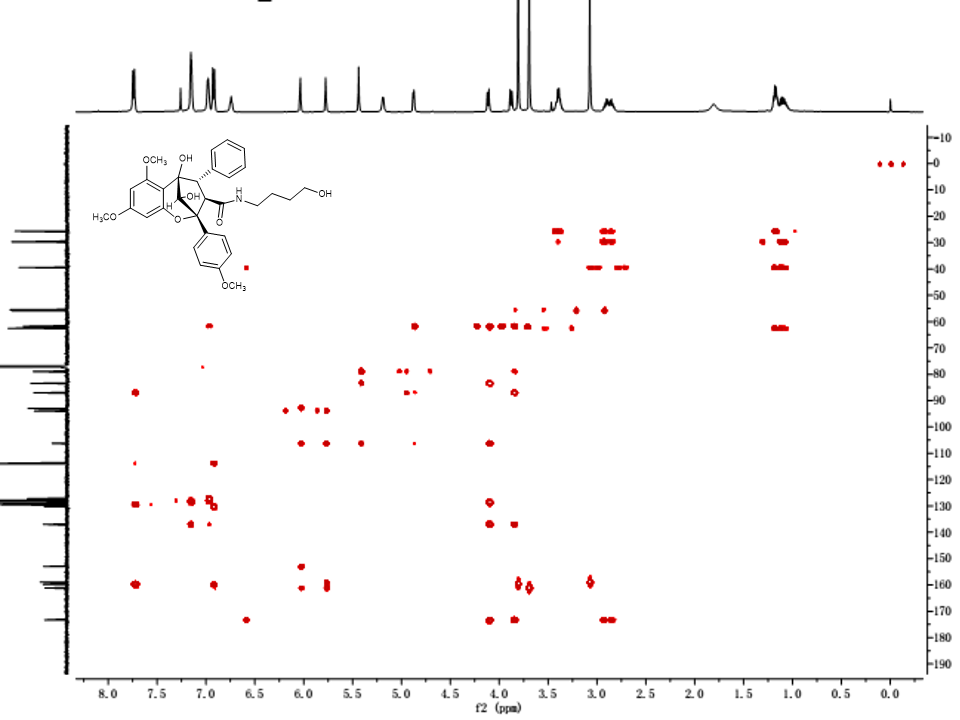


**Figure S8-5.** ROESY spectrum of compound **8** (CDCl_3_).


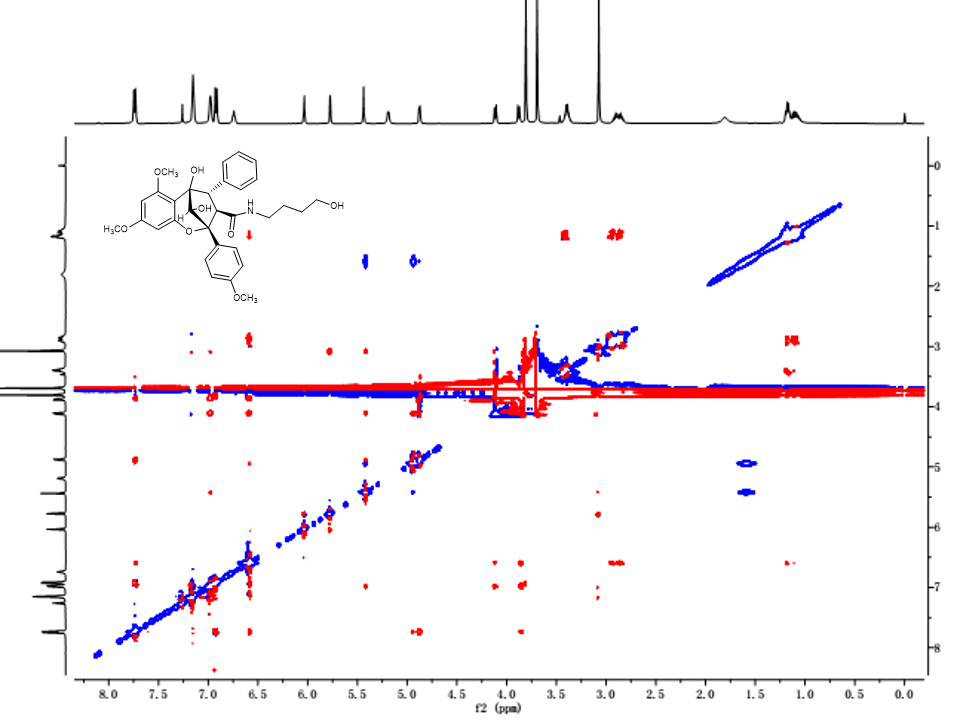


**Figure S9-6.** HRESIMS of compound **9**.


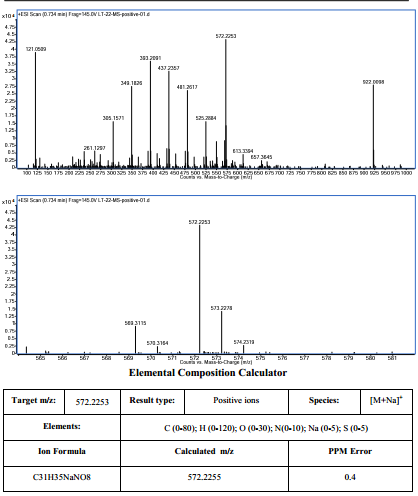


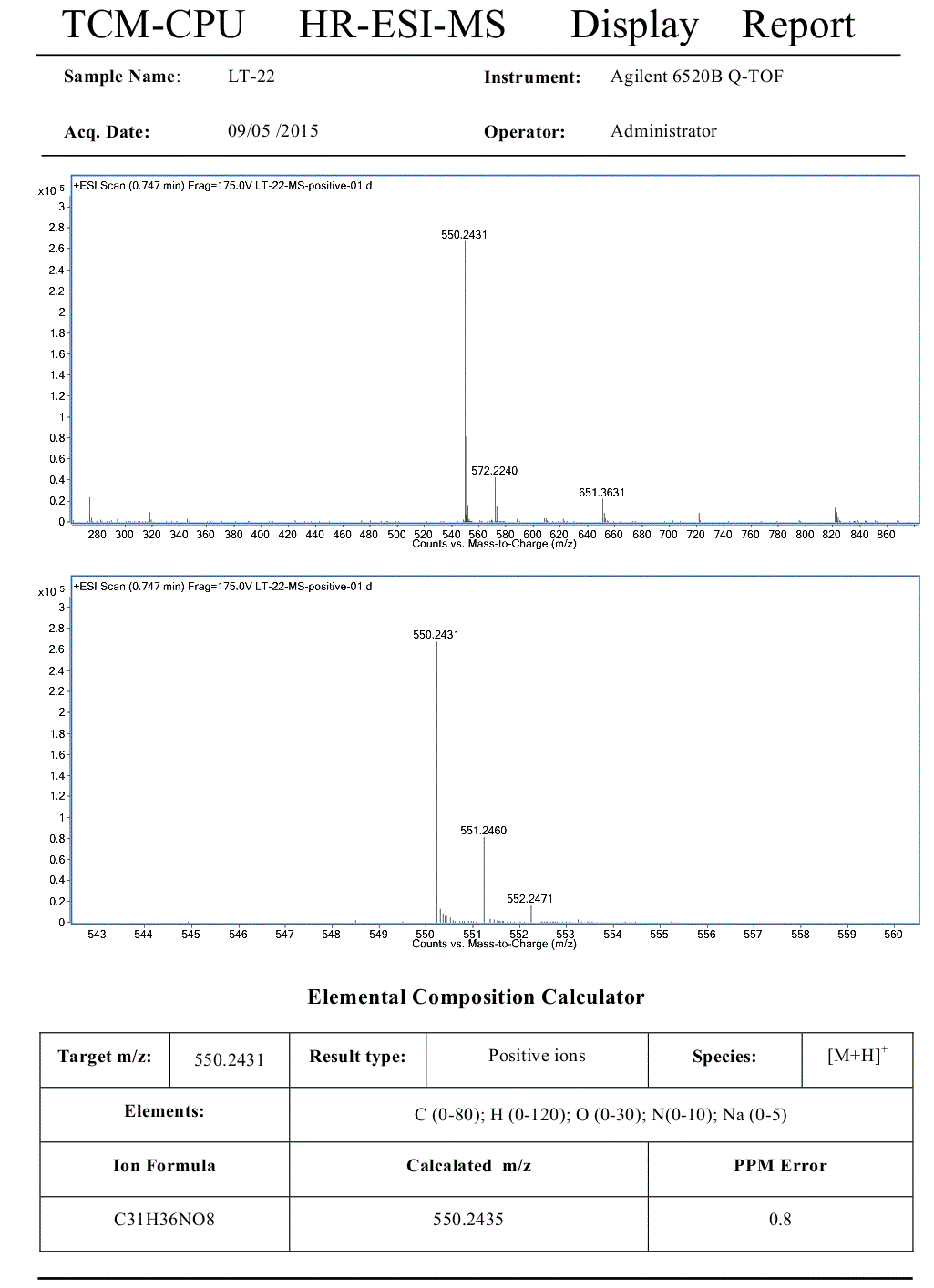


**Figure S10-1.** ^1^H NMR spectrum of compound **1a** (CDCl_3_).


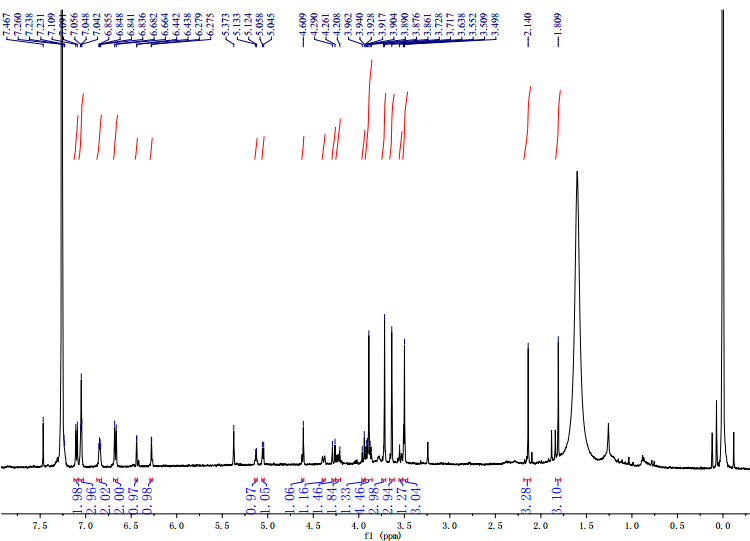


**Figure S10-2.** HRESIMS of compound **1a**.


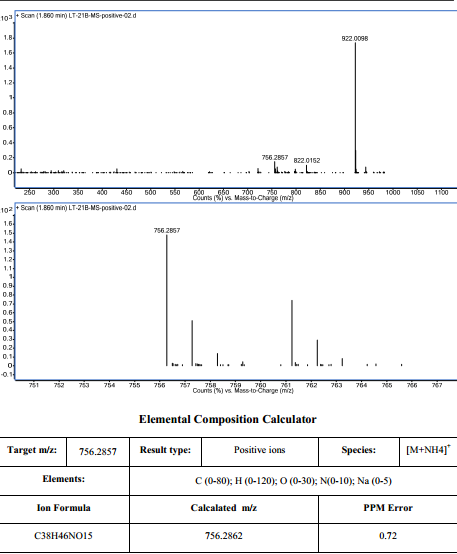


**Figure S11-1.** ^1^H NMR spectrum of compound **10** (CDCl_3_).


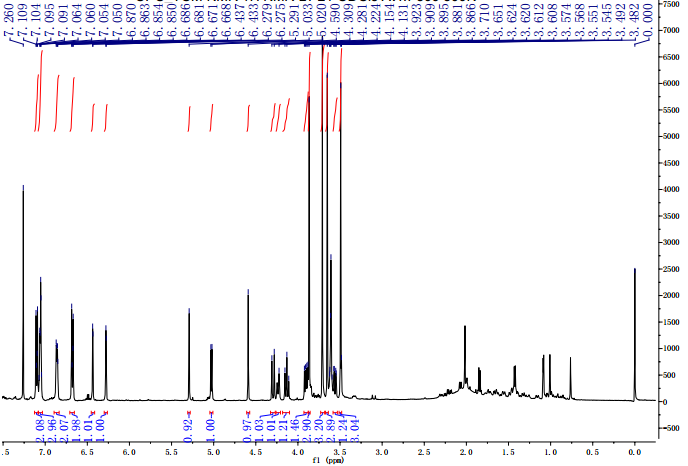


**Figure S11-2.** HRESIMS of compound **10**.


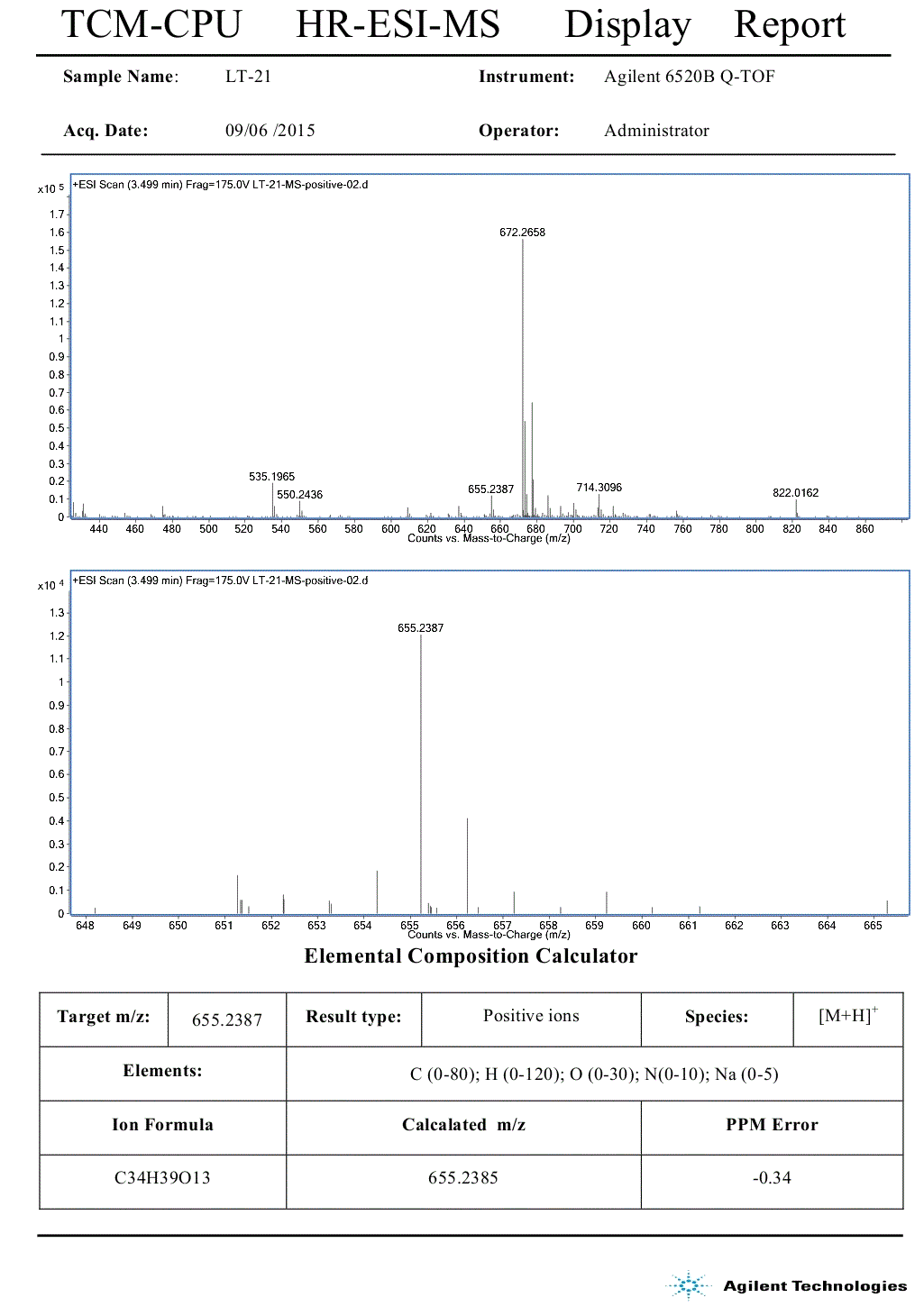


**Figure S11-3.** ^1^H NMR spectrum of compound **10a** (CDCl_3_).


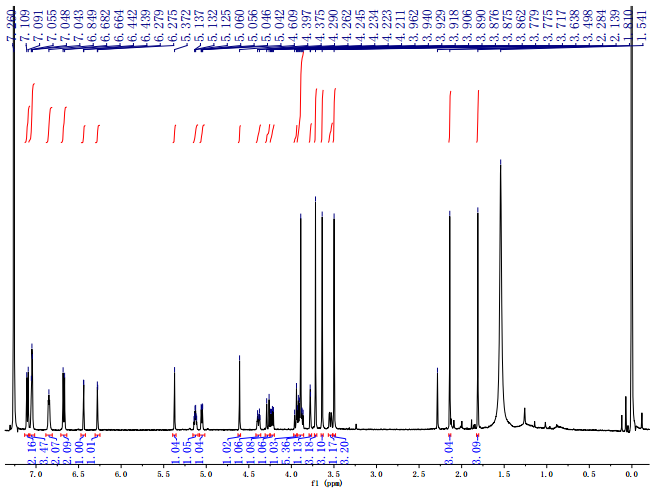


**Figure S11-4.** HRESIMS of compound **10a**.


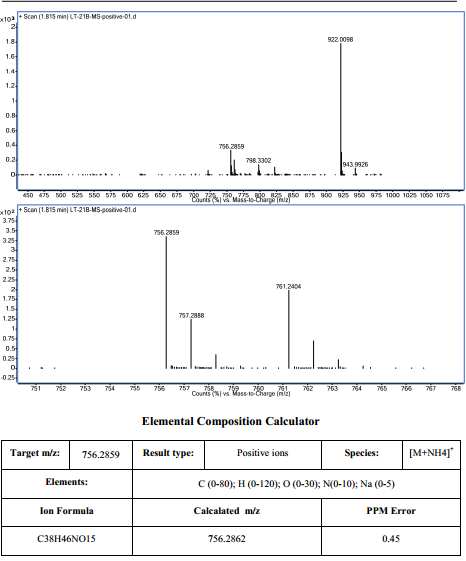


**Figure S12-1.** ^1^H NMR spectrum of compound **11** (CDCl_3_).


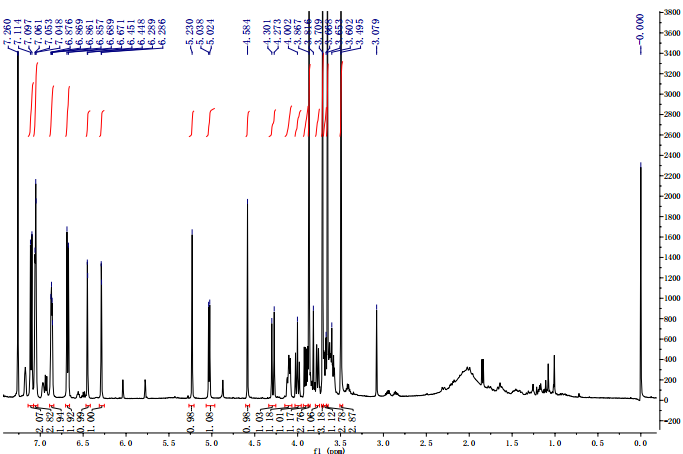


**Figure S12-2.** HRESIMS of compound **11**


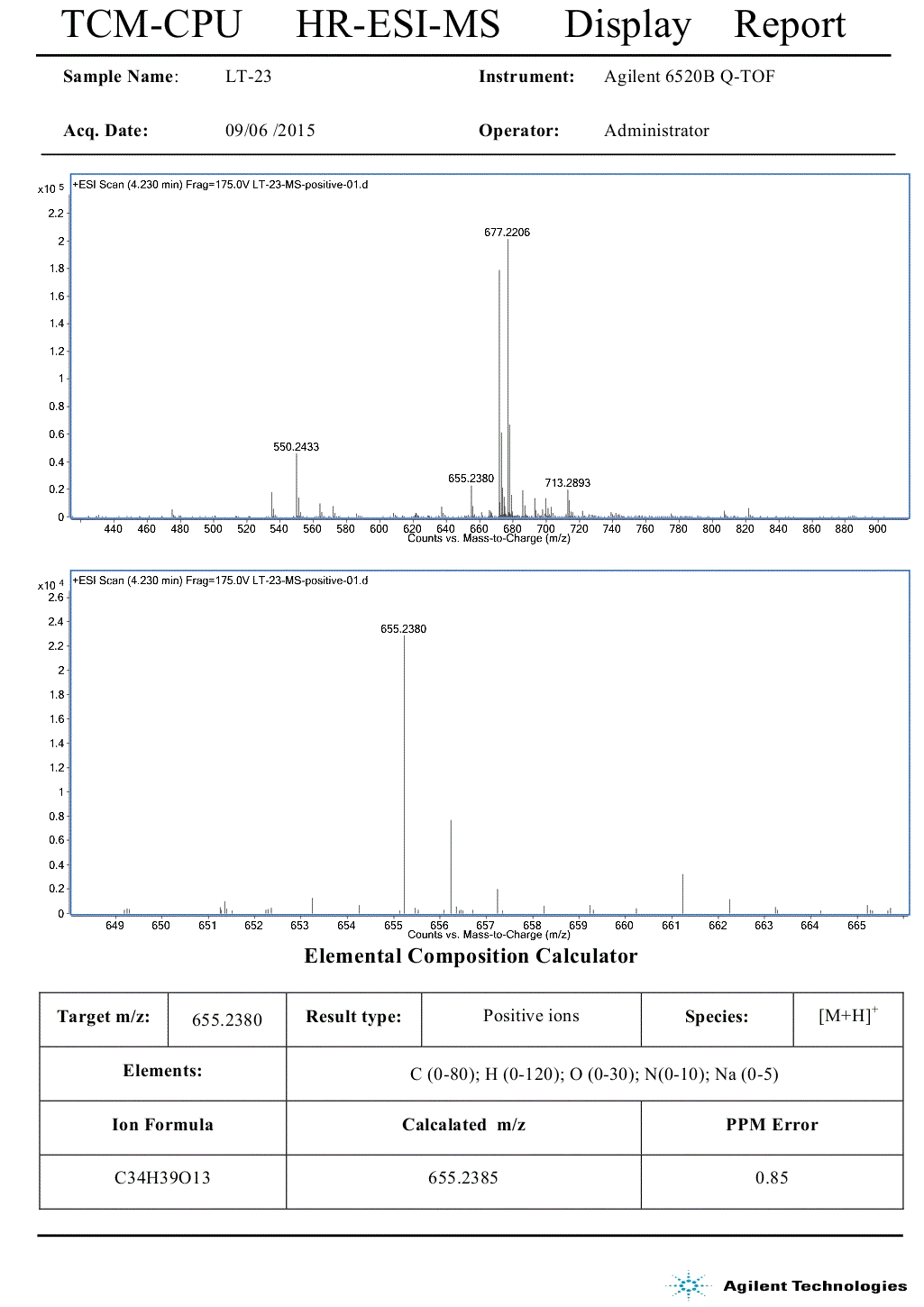
.

**Figure S12-3.** ^1^H NMR spectrum of compound **11a** (CDCl_3_).


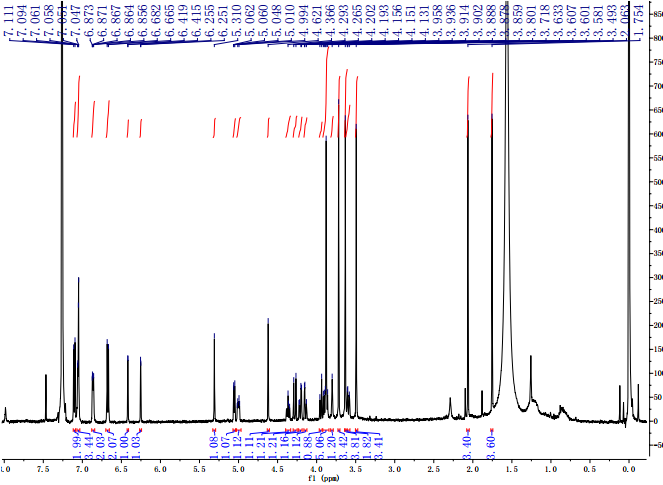


**Figure S12-4.** HRESIMS of compound **11a**.


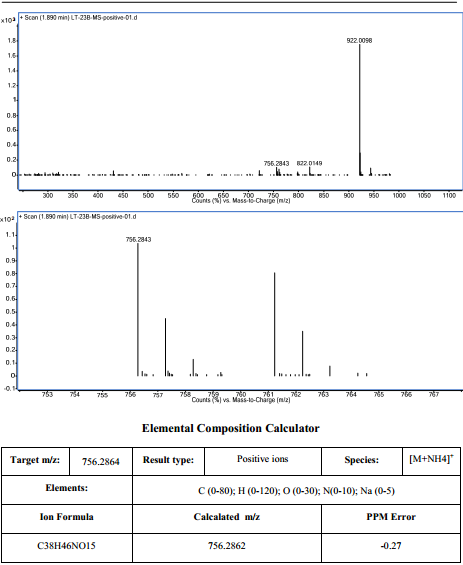


**Figure S13-1.** ^1^H NMR spectrum of compound **12** (CDCl_3_).


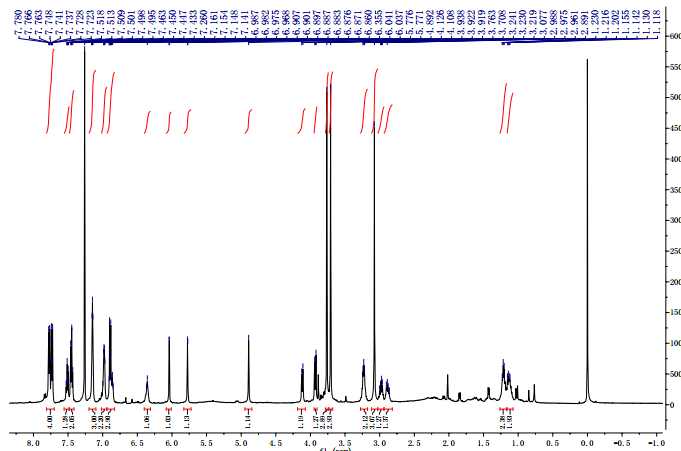


**Figure S13-2.** HRESIMS of compound **12**.


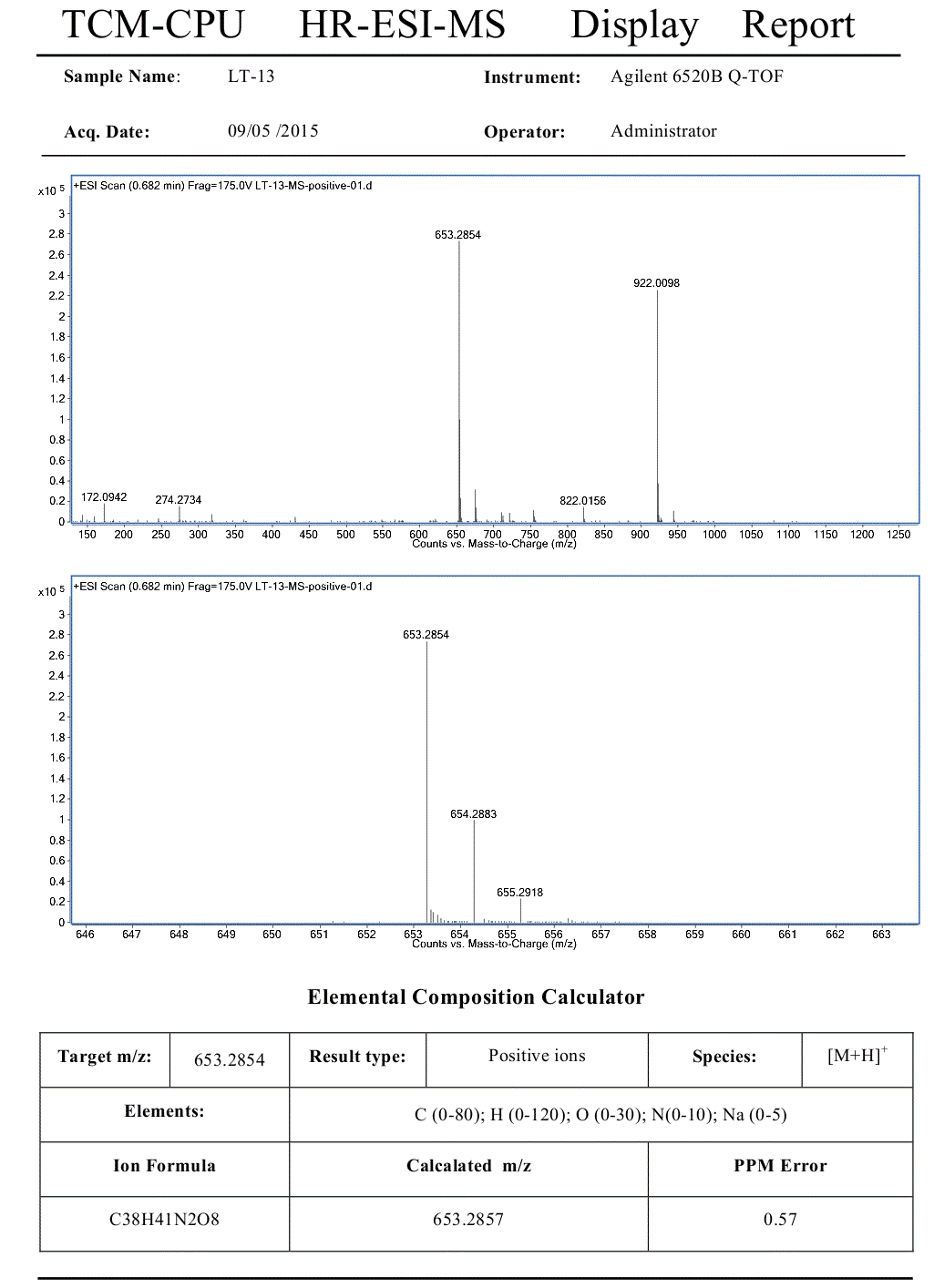


**Figure S14-1.** ^1^H NMR spectrum of compound **13** (CDCl_3_).


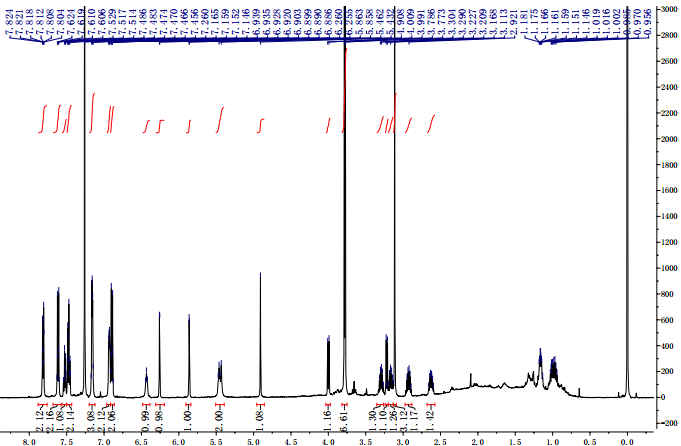


**Figure S14-2.** HRESIMS of compound **13**.


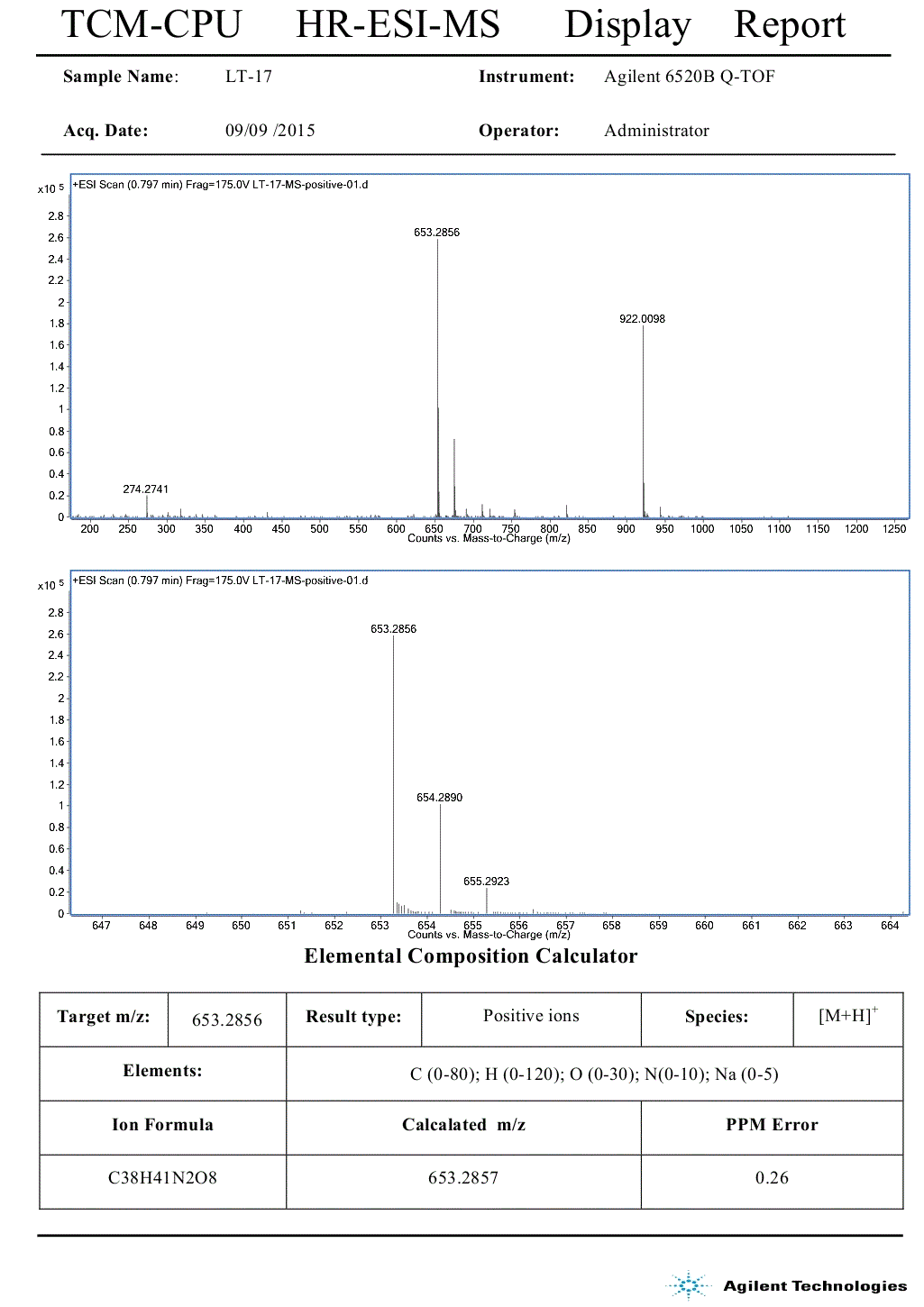


**Figure S15-1.** ^1^H NMR spectrum of compound **14** (CDCl_3_).


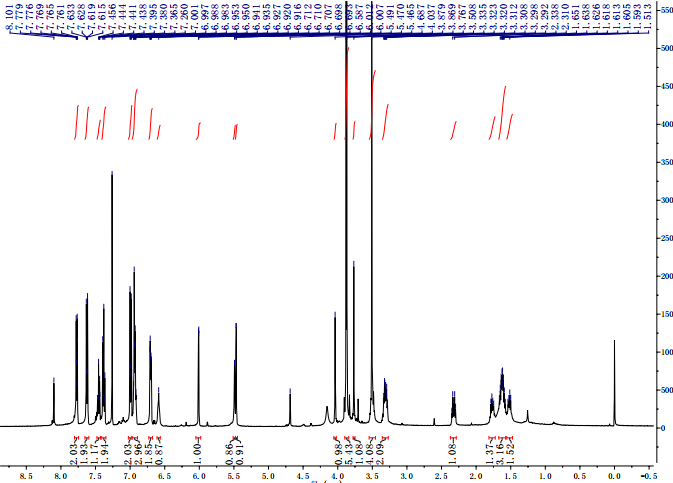


**Figure S15-2.** HRESIMS of compound **14**.


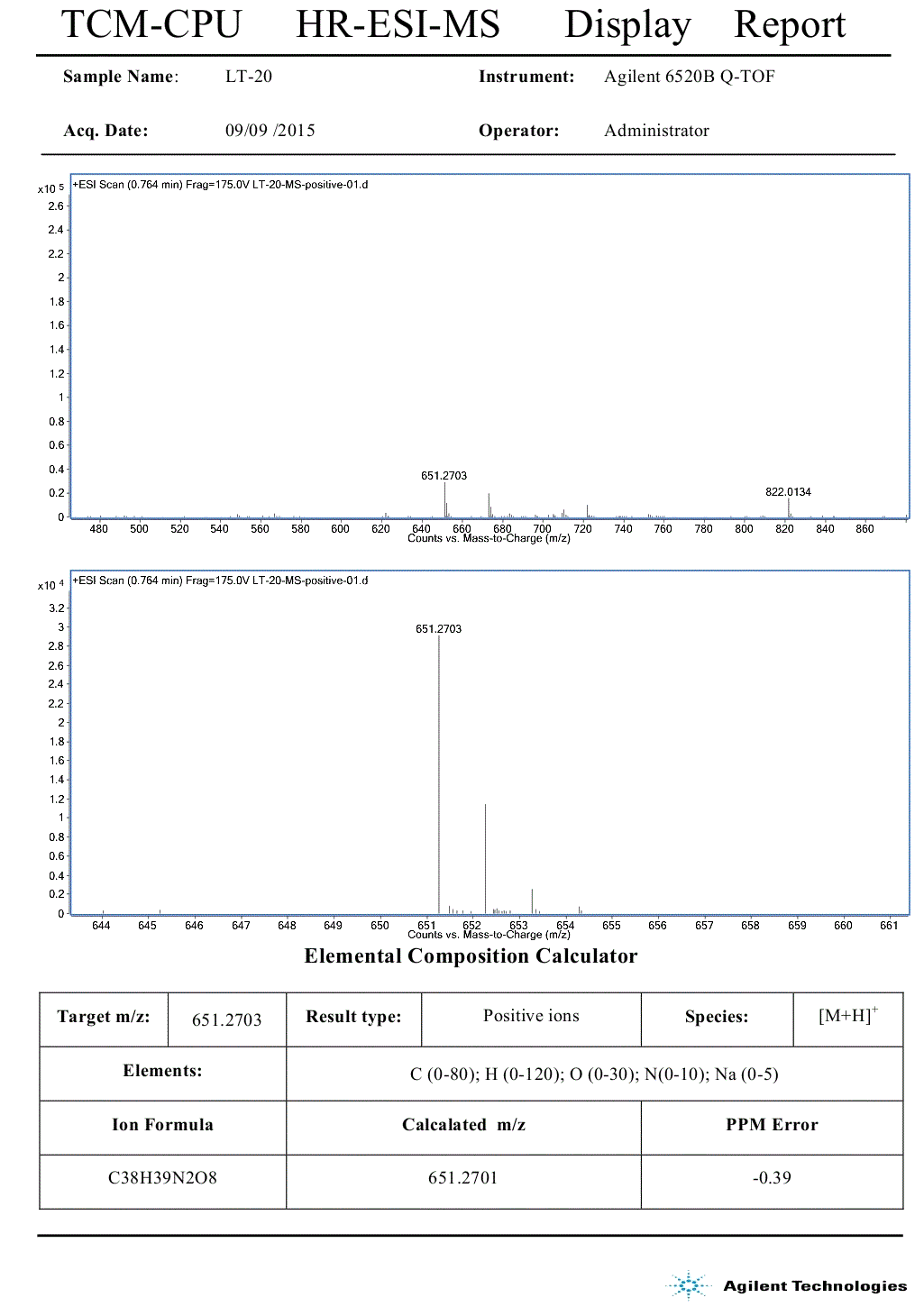


**Figure S16.** Structures of compound **1**, **1a**, **10**, **10a**, **11**, **11a**.


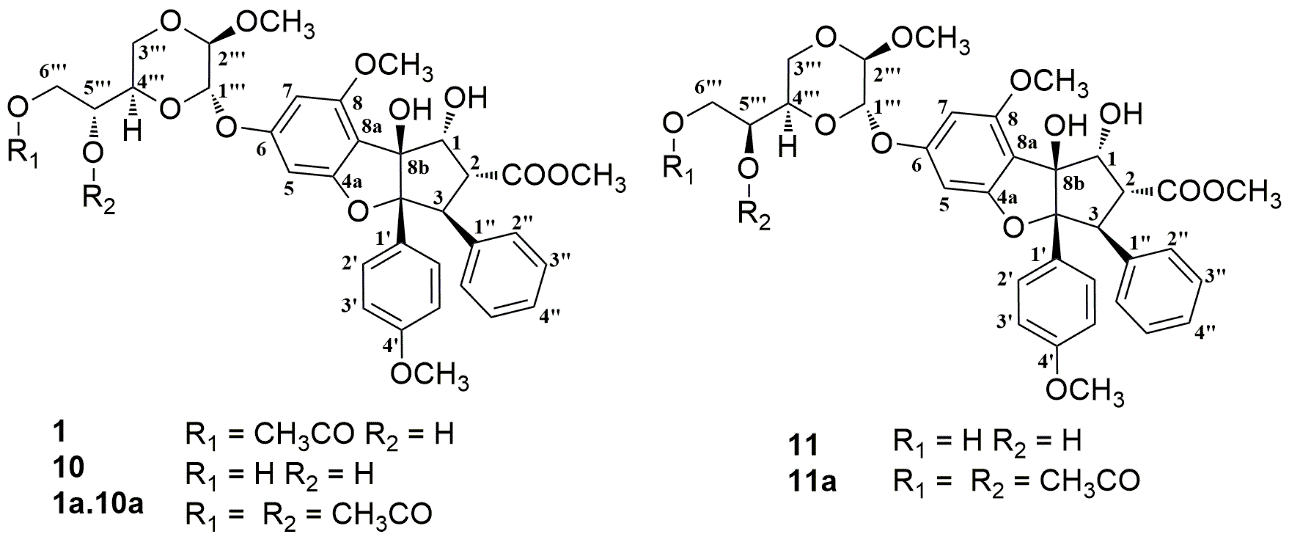


**Table 1.** Conformers of compound **8** and its calculated ECD data.

| No | conformer | calculated ECD data |
| --- | --- | --- |
| 1 | 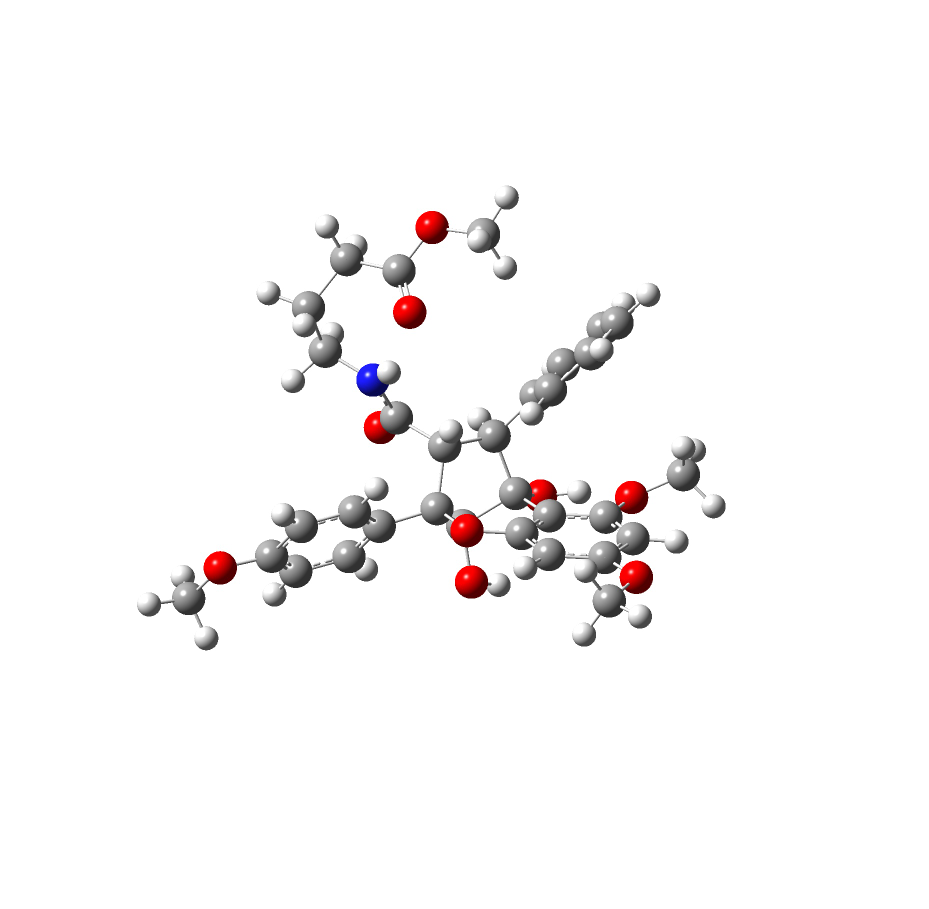 | 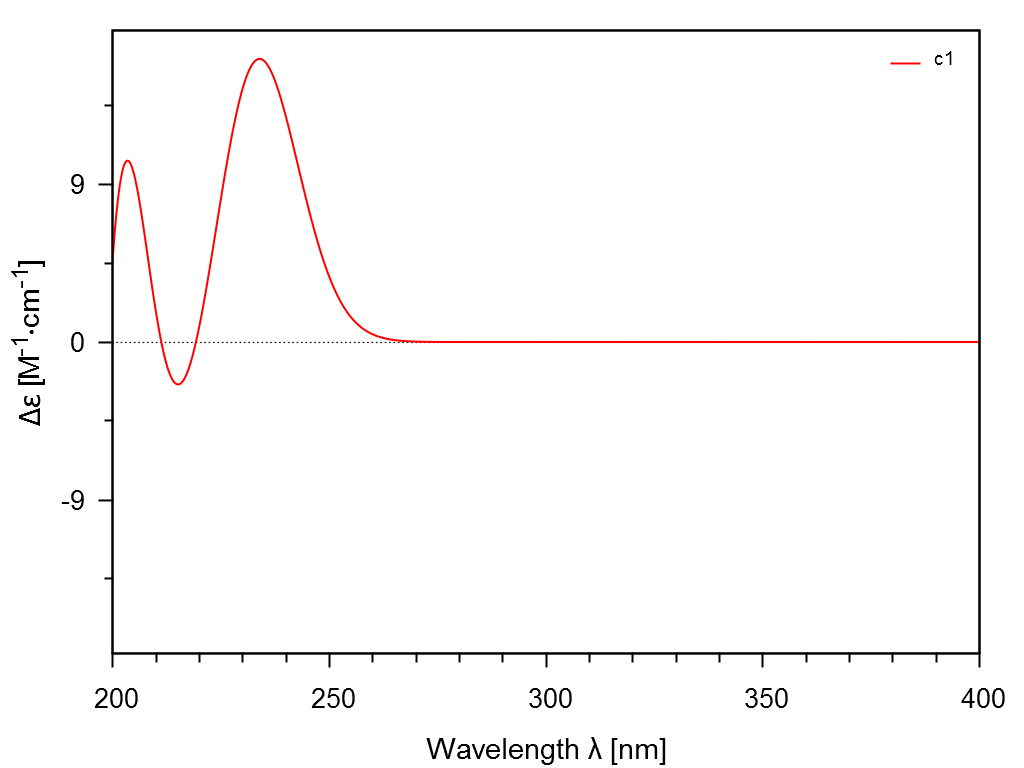 |
| 2 | 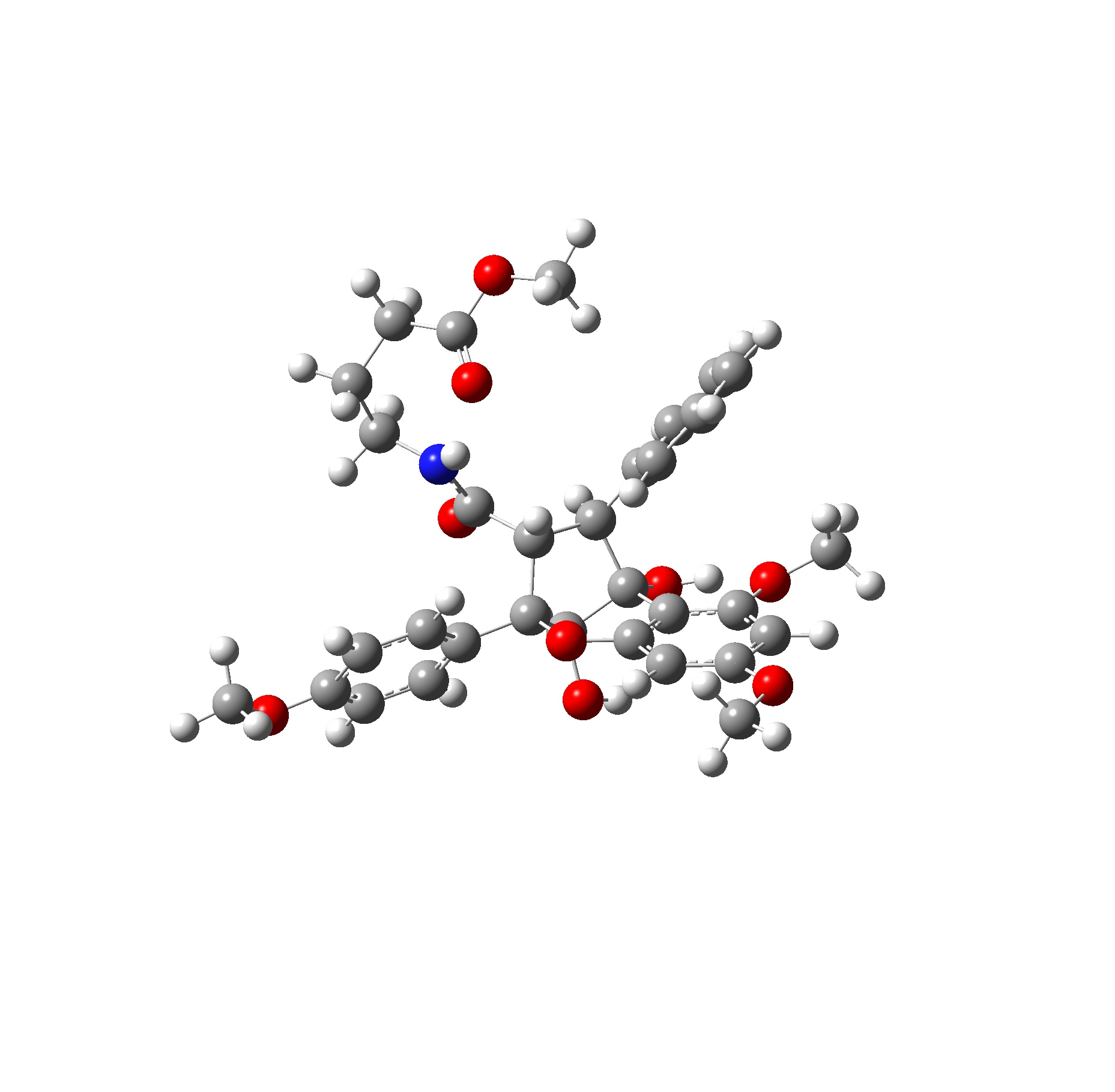 | 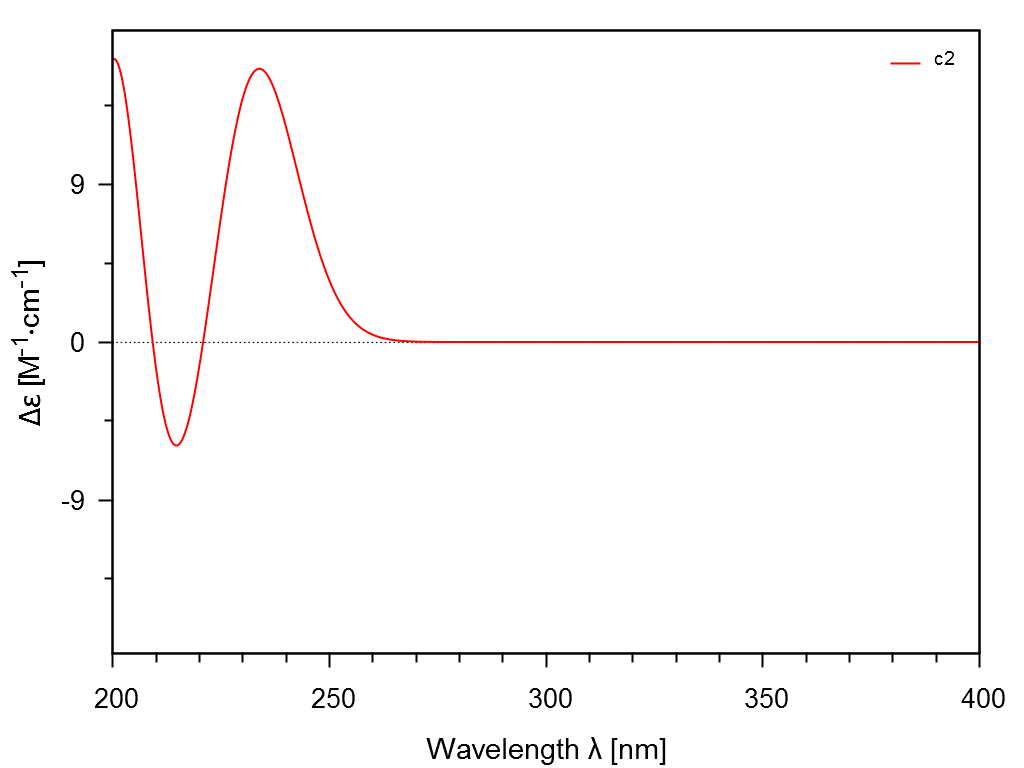 |
| 3 | 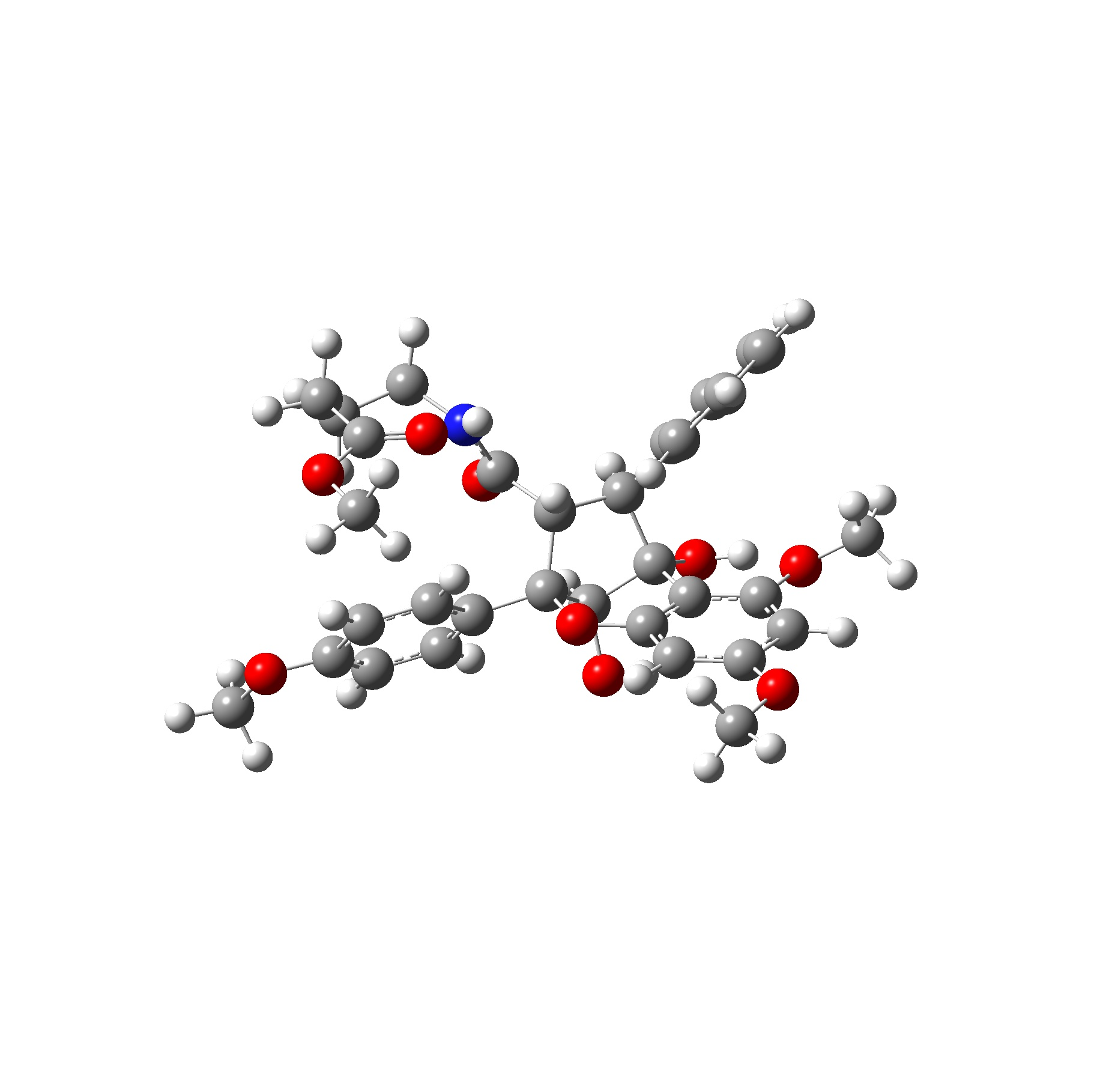 | 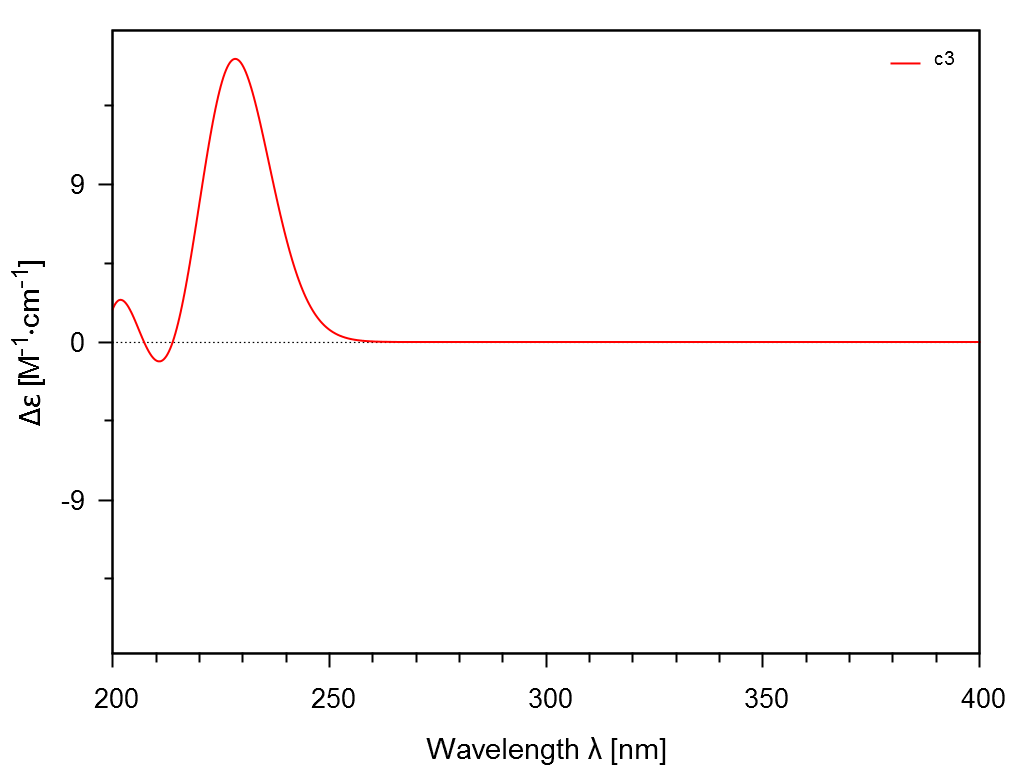 |
| 4 | 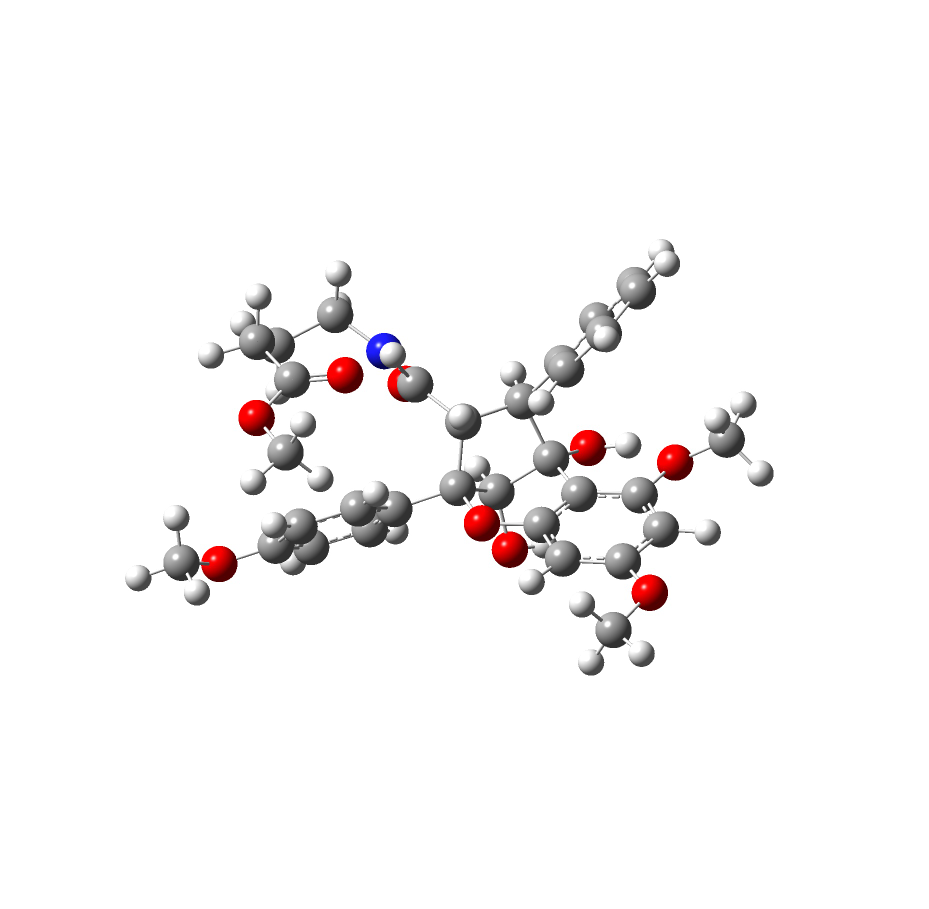 | 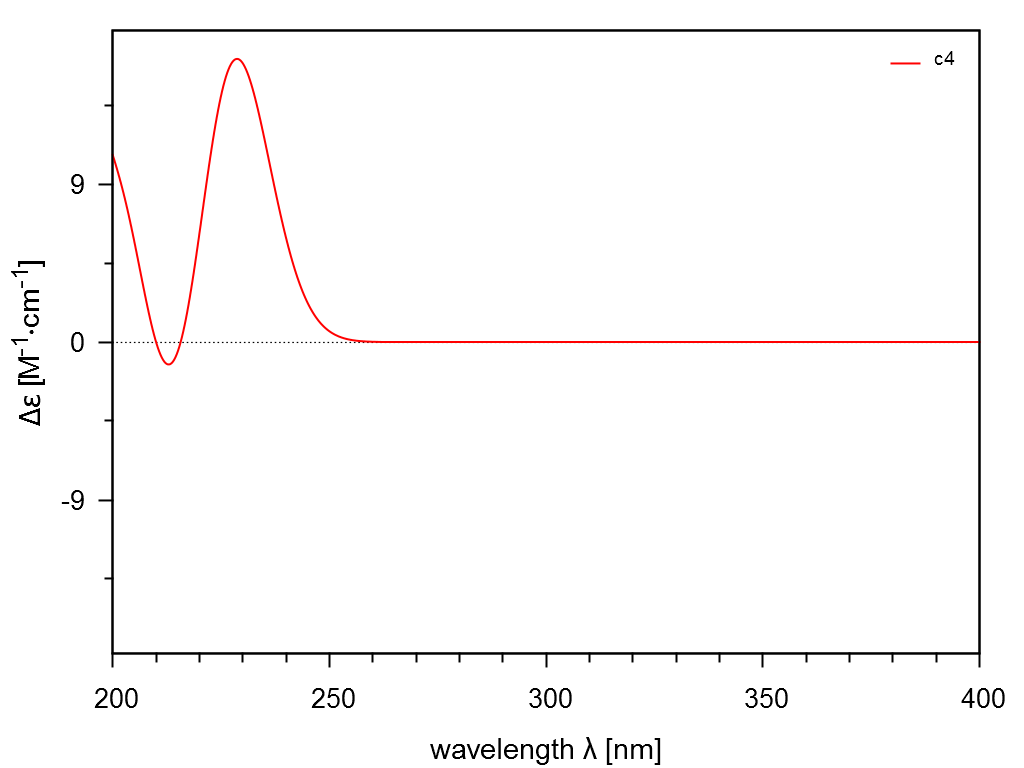 |
| 5 | 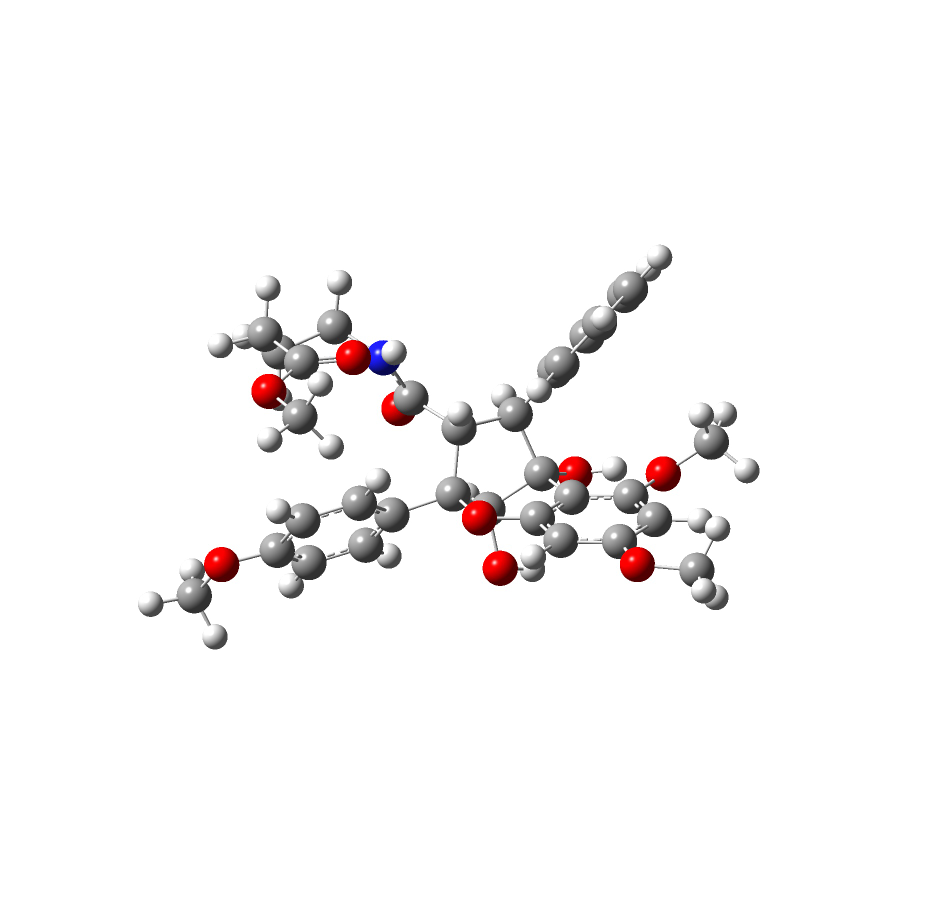 | 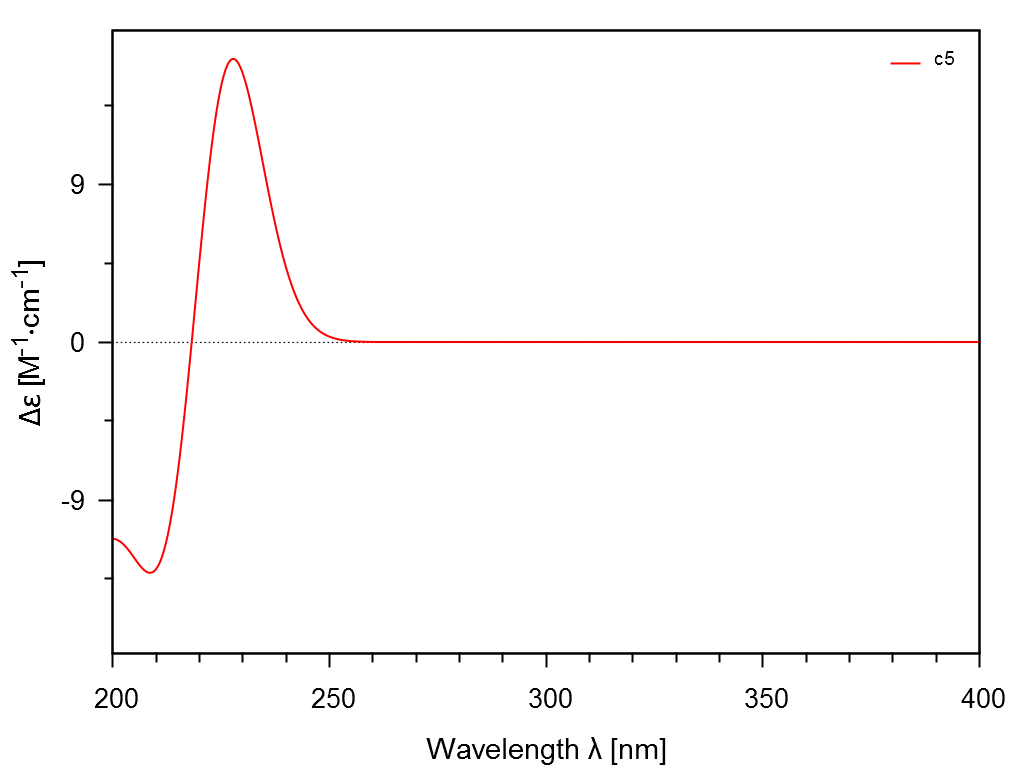 |

**Table 2.** Optimized Z-Matrixes of compound **8** in the Gas Phase (Å) at B3LYP/6-311G (d, p) level.

| C1： | | | | | |
| --- | --- | --- | --- | --- | --- |
| Center  Number | Atomic  Number | Atomic  type | Coordinates (Angstroms) | | |
|  |  |  | x | y | z |
| 1 | 6 | 0 | 4.197253 | -2.093807 | 0.615472 |
| 2 | 6 | 0 | 3.495509 | -2.563468 | 1.729180 |
| 3 | 6 | 0 | 2.106987 | -2.487314 | 1.740746 |
| 4 | 6 | 0 | 1.431345 | -1.966654 | 0.636499 |
| 5 | 6 | 0 | 2.118790 | -1.525337 | -0.500519 |
| 6 | 6 | 0 | 3.522123 | -1.558555 | -0.487742 |
| 7 | 8 | 0 | 0.069175 | -1.895114 | 0.750050 |
| 8 | 6 | 0 | -0.678296 | -1.348476 | -0.376546 |
| 9 | 6 | 0 | -0.487580 | 0.207599 | -0.388782 |
| 10 | 6 | 0 | 0.742831 | 0.456879 | -1.318743 |
| 11 | 6 | 0 | 1.276999 | -0.969430 | -1.637614 |
| 12 | 6 | 0 | -1.686986 | 0.912197 | -1.005677 |
| 13 | 6 | 0 | -1.623594 | 4.569835 | 1.147762 |
| 14 | 6 | 0 | -2.924883 | 4.848275 | 0.430354 |
| 15 | 6 | 0 | -3.874685 | 3.648951 | 0.398098 |
| 16 | 6 | 0 | -3.457959 | 2.568538 | -0.599079 |
| 17 | 7 | 0 | -2.270959 | 1.853207 | -0.192163 |
| 18 | 6 | 0 | -2.115366 | -1.786100 | -0.101514 |
| 19 | 6 | 0 | -2.732412 | -1.427615 | 1.111940 |
| 20 | 6 | 0 | -4.052844 | -1.793192 | 1.386977 |
| 21 | 6 | 0 | -4.791872 | -2.531486 | 0.464568 |
| 22 | 6 | 0 | -4.192499 | -2.905652 | -0.735852 |
| 23 | 6 | 0 | -2.867862 | -2.539028 | -1.013503 |
| 24 | 6 | 0 | 2.146198 | 1.609034 | 0.510297 |
| 25 | 6 | 0 | 1.787118 | 1.455984 | -0.836941 |
| 26 | 6 | 0 | 2.464370 | 2.232858 | -1.793212 |
| 27 | 6 | 0 | 3.458387 | 3.137512 | -1.415036 |
| 28 | 6 | 0 | 3.800817 | 3.274711 | -0.073570 |
| 29 | 6 | 0 | 3.147635 | 2.508284 | 0.887430 |
| 30 | 8 | 0 | 1.899987 | -1.031207 | -2.920081 |
| 31 | 8 | 0 | -2.085779 | 0.670209 | -2.141466 |
| 32 | 6 | 0 | -0.040367 | -1.770366 | -1.717338 |
| 33 | 8 | 0 | 0.180382 | -3.173952 | -1.890093 |
| 34 | 8 | 0 | -1.355576 | 3.539194 | 1.753319 |
| 35 | 8 | 0 | -0.798499 | 5.645992 | 1.021723 |
| 36 | 6 | 0 | 0.480783 | 5.475028 | 1.632075 |
| 37 | 8 | 0 | 4.164061 | -1.068948 | -1.597462 |
| 38 | 6 | 0 | 5.451425 | -0.502074 | -1.361877 |
| 39 | 8 | 0 | 4.282070 | -3.055574 | 2.734948 |
| 40 | 6 | 0 | 3.610251 | -3.641763 | 3.843856 |
| 41 | 8 | 0 | -6.070560 | -2.821317 | 0.853419 |
| 42 | 6 | 0 | -6.856865 | -3.582975 | -0.054055 |
| 43 | 1 | 0 | 5.280150 | -2.169467 | 0.629036 |
| 44 | 1 | 0 | 1.518377 | -2.823148 | 2.587517 |
| 45 | 1 | 0 | -0.331426 | 0.573328 | 0.630378 |
| 46 | 1 | 0 | 0.375992 | 0.875170 | -2.268775 |
| 47 | 1 | 0 | -3.416377 | 5.670211 | 0.963707 |
| 48 | 1 | 0 | -2.703812 | 5.177651 | -0.590956 |
| 49 | 1 | 0 | -3.977894 | 3.215930 | 1.400585 |
| 50 | 1 | 0 | -4.866614 | 4.012705 | 0.103055 |
| 51 | 1 | 0 | -4.261167 | 1.828826 | -0.695924 |
| 52 | 1 | 0 | -3.286971 | 2.997174 | -1.593250 |
| 53 | 1 | 0 | -1.876480 | 2.039525 | 0.741007 |
| 54 | 1 | 0 | -2.182855 | -0.861436 | 1.861296 |
| 55 | 1 | 0 | -4.506130 | -1.502616 | 2.331106 |
| 56 | 1 | 0 | -4.722039 | -3.485119 | -1.484521 |
| 57 | 1 | 0 | -2.442014 | -2.859006 | -1.961793 |
| 58 | 1 | 0 | 1.678982 | 1.016195 | 1.292462 |
| 59 | 1 | 0 | 2.224113 | 2.131545 | -2.850901 |
| 60 | 1 | 0 | 3.967700 | 3.729390 | -2.171074 |
| 61 | 1 | 0 | 4.578741 | 3.973213 | 0.222080 |
| 62 | 1 | 0 | 3.419471 | 2.603569 | 1.935777 |
| 63 | 1 | 0 | 2.860563 | -0.936839 | -2.765216 |
| 64 | 1 | 0 | -0.633120 | -1.445969 | -2.581686 |
| 65 | 1 | 0 | 0.853494 | -3.244799 | -2.593577 |
| 66 | 1 | 0 | 1.056753 | 6.391286 | 1.476154 |
| 67 | 1 | 0 | 0.374596 | 5.310504 | 2.708820 |
| 68 | 1 | 0 | 1.015734 | 4.645522 | 1.162930 |
| 69 | 1 | 0 | 6.205139 | -1.288534 | -1.257292 |
| 70 | 1 | 0 | 5.714931 | 0.097487 | -2.238508 |
| 71 | 1 | 0 | 5.450616 | 0.162669 | -0.491070 |
| 72 | 1 | 0 | 4.371067 | -4.030938 | 4.527371 |
| 73 | 1 | 0 | 3.024941 | -2.893725 | 4.388609 |
| 74 | 1 | 0 | 2.983733 | -4.482992 | 3.528855 |
| 75 | 1 | 0 | -7.841536 | -3.730830 | 0.399855 |
| 76 | 1 | 0 | -7.002964 | -3.046031 | -0.997190 |
| 77 | 1 | 0 | -6.417576 | -4.571535 | -0.224493 |
| C2： | | | | | |
| Center  Number | Atomic  Number | Atomic  type | Coordinates (Angstroms) | | |
|  |  |  | x | y | z |
| 1 | 6 | 0 | 4.313408 | -1.813486 | 0.710939 |
| 2 | 6 | 0 | 3.595376 | -2.367448 | 1.774333 |
| 3 | 6 | 0 | 2.206258 | -2.395026 | 1.716826 |
| 4 | 6 | 0 | 1.548053 | -1.893288 | 0.593464 |
| 5 | 6 | 0 | 2.255297 | -1.368876 | -0.495189 |
| 6 | 6 | 0 | 3.654771 | -1.297655 | -0.411314 |
| 7 | 8 | 0 | 0.180255 | -1.925683 | 0.639177 |
| 8 | 6 | 0 | -0.550186 | -1.404173 | -0.510812 |
| 9 | 6 | 0 | -0.477490 | 0.161135 | -0.473269 |
| 10 | 6 | 0 | 0.775002 | 0.528202 | -1.332177 |
| 11 | 6 | 0 | 1.430806 | -0.844280 | -1.659591 |
| 12 | 6 | 0 | -1.694595 | 0.793538 | -1.131795 |
| 13 | 6 | 0 | -2.038873 | 4.346707 | 1.164446 |
| 14 | 6 | 0 | -3.307047 | 4.561514 | 0.369916 |
| 15 | 6 | 0 | -4.163576 | 3.300630 | 0.233385 |
| 16 | 6 | 0 | -3.608654 | 2.293496 | -0.773143 |
| 17 | 7 | 0 | -2.396488 | 1.652028 | -0.319807 |
| 18 | 6 | 0 | -1.961854 | -1.955201 | -0.320867 |
| 19 | 6 | 0 | -2.614424 | -2.722325 | -1.294047 |
| 20 | 6 | 0 | -3.916227 | -3.191258 | -1.084082 |
| 21 | 6 | 0 | -4.596873 | -2.914429 | 0.101317 |
| 22 | 6 | 0 | -3.960303 | -2.162303 | 1.084440 |
| 23 | 6 | 0 | -2.658294 | -1.691478 | 0.875390 |
| 24 | 6 | 0 | 1.996093 | 1.726303 | 0.595638 |
| 25 | 6 | 0 | 1.714374 | 1.588536 | -0.771595 |
| 26 | 6 | 0 | 2.373323 | 2.444821 | -1.671064 |
| 27 | 6 | 0 | 3.273889 | 3.411297 | -1.219018 |
| 28 | 6 | 0 | 3.540142 | 3.532496 | 0.141146 |
| 29 | 6 | 0 | 2.904737 | 2.687844 | 1.046866 |
| 30 | 8 | 0 | 2.119024 | -0.821128 | -2.909623 |
| 31 | 8 | 0 | -2.010560 | 0.567449 | -2.296399 |
| 32 | 6 | 0 | 0.183494 | -1.738645 | -1.827033 |
| 33 | 8 | 0 | 0.518639 | -3.116161 | -2.021587 |
| 34 | 8 | 0 | -1.736421 | 3.312963 | 1.748429 |
| 35 | 8 | 0 | -1.289165 | 5.483116 | 1.136028 |
| 36 | 6 | 0 | -0.044280 | 5.378742 | 1.827161 |
| 37 | 8 | 0 | 4.311373 | -0.728992 | -1.473647 |
| 38 | 6 | 0 | 5.540001 | -0.076700 | -1.158842 |
| 39 | 8 | 0 | 4.366510 | -2.829432 | 2.806086 |
| 40 | 6 | 0 | 3.688682 | -3.508103 | 3.857004 |
| 41 | 8 | 0 | -5.860280 | -3.432375 | 0.173290 |
| 42 | 6 | 0 | -6.582279 | -3.197567 | 1.374956 |
| 43 | 1 | 0 | 5.396983 | -1.808652 | 0.778100 |
| 44 | 1 | 0 | 1.604020 | -2.798748 | 2.523479 |
| 45 | 1 | 0 | -0.399203 | 0.507339 | 0.561674 |
| 46 | 1 | 0 | 0.425265 | 0.945383 | -2.289121 |
| 47 | 1 | 0 | -3.890506 | 5.325847 | 0.896255 |
| 48 | 1 | 0 | -3.043959 | 4.944593 | -0.622256 |
| 49 | 1 | 0 | -4.296711 | 2.821010 | 1.210828 |
| 50 | 1 | 0 | -5.159204 | 3.605248 | -0.111741 |
| 51 | 1 | 0 | -4.346534 | 1.501110 | -0.944508 |
| 52 | 1 | 0 | -3.413149 | 2.772416 | -1.739434 |
| 53 | 1 | 0 | -2.070760 | 1.830065 | 0.640938 |
| 54 | 1 | 0 | -2.129461 | -2.973997 | -2.234031 |
| 55 | 1 | 0 | -4.401198 | -3.781882 | -1.857652 |
| 56 | 1 | 0 | -4.442146 | -1.925364 | 2.026417 |
| 57 | 1 | 0 | -2.181543 | -1.115855 | 1.666922 |
| 58 | 1 | 0 | 1.540469 | 1.074548 | 1.336714 |
| 59 | 1 | 0 | 2.192432 | 2.358525 | -2.741830 |
| 60 | 1 | 0 | 3.770549 | 4.064027 | -1.932182 |
| 61 | 1 | 0 | 4.245943 | 4.279317 | 0.494180 |
| 62 | 1 | 0 | 3.118630 | 2.770638 | 2.109488 |
| 63 | 1 | 0 | 3.061518 | -0.664669 | -2.703424 |
| 64 | 1 | 0 | -0.388694 | -1.434714 | -2.712520 |
| 65 | 1 | 0 | 1.219021 | -3.118242 | -2.701567 |
| 66 | 1 | 0 | 0.475976 | 6.336181 | 1.736763 |
| 67 | 1 | 0 | -0.210646 | 5.172539 | 2.888964 |
| 68 | 1 | 0 | 0.577341 | 4.602647 | 1.373428 |
| 69 | 1 | 0 | 5.800436 | 0.565961 | -2.005383 |
| 70 | 1 | 0 | 5.447318 | 0.560339 | -0.272455 |
| 71 | 1 | 0 | 6.344222 | -0.808729 | -1.036451 |
| 72 | 1 | 0 | 4.442752 | -3.863066 | 4.566042 |
| 73 | 1 | 0 | 3.019463 | -2.828937 | 4.395261 |
| 74 | 1 | 0 | 3.147297 | -4.382573 | 3.480783 |
| 75 | 1 | 0 | -7.559882 | -3.679515 | 1.277323 |
| 76 | 1 | 0 | -6.074758 | -3.646083 | 2.235347 |
| 77 | 1 | 0 | -6.753371 | -2.127077 | 1.529916 |
| C3： | | | | | |
| Center  Number | Atomic  Number | Atomic  type | Coordinates (Angstroms) | | |
|  |  |  | x | y | z |
| 1 | 6 | 0 | 4.491334 | -1.630441 | 0.892776 |
| 2 | 6 | 0 | 3.651477 | -2.222735 | 1.839867 |
| 3 | 6 | 0 | 2.274212 | -2.175000 | 1.652752 |
| 4 | 6 | 0 | 1.748989 | -1.559685 | 0.515826 |
| 5 | 6 | 0 | 2.579219 | -0.994530 | -0.459440 |
| 6 | 6 | 0 | 3.965781 | -1.001064 | -0.241438 |
| 7 | 8 | 0 | 0.382969 | -1.527250 | 0.431849 |
| 8 | 6 | 0 | -0.209573 | -0.870386 | -0.728692 |
| 9 | 6 | 0 | -0.052505 | 0.679188 | -0.552977 |
| 10 | 6 | 0 | 1.282925 | 1.034947 | -1.282039 |
| 11 | 6 | 0 | 1.895317 | -0.345505 | -1.651245 |
| 12 | 6 | 0 | -1.167511 | 1.448957 | -1.244118 |
| 13 | 6 | 0 | -3.771924 | 2.116734 | 2.050015 |
| 14 | 6 | 0 | -4.602864 | 2.872109 | 1.037607 |
| 15 | 6 | 0 | -4.258608 | 2.549030 | -0.415975 |
| 16 | 6 | 0 | -2.931414 | 3.147444 | -0.884724 |
| 17 | 7 | 0 | -1.803746 | 2.361884 | -0.437599 |
| 18 | 6 | 0 | -1.660926 | -1.344617 | -0.708144 |
| 19 | 6 | 0 | -2.445864 | -1.130067 | 0.439893 |
| 20 | 6 | 0 | -3.783500 | -1.531374 | 0.486797 |
| 21 | 6 | 0 | -4.374326 | -2.159769 | -0.607576 |
| 22 | 6 | 0 | -3.608428 | -2.391359 | -1.747594 |
| 23 | 6 | 0 | -2.265498 | -1.991169 | -1.794935 |
| 24 | 6 | 0 | 2.371854 | 2.069131 | 0.812009 |
| 25 | 6 | 0 | 2.221151 | 2.007700 | -0.580894 |
| 26 | 6 | 0 | 3.006471 | 2.867278 | -1.369432 |
| 27 | 6 | 0 | 3.899026 | 3.767875 | -0.785207 |
| 28 | 6 | 0 | 4.027755 | 3.820231 | 0.598944 |
| 29 | 6 | 0 | 3.267108 | 2.969618 | 1.396121 |
| 30 | 8 | 0 | 2.698433 | -0.277852 | -2.829012 |
| 31 | 8 | 0 | -1.447631 | 1.281924 | -2.427678 |
| 32 | 6 | 0 | 0.622815 | -1.148529 | -1.997787 |
| 33 | 8 | 0 | 0.899890 | -2.525349 | -2.273214 |
| 34 | 8 | 0 | -2.551173 | 2.183789 | 2.137058 |
| 35 | 8 | 0 | -4.573452 | 1.387717 | 2.873485 |
| 36 | 6 | 0 | -3.879947 | 0.694449 | 3.911969 |
| 37 | 8 | 0 | 4.747094 | -0.392671 | -1.191558 |
| 38 | 6 | 0 | 5.949663 | 0.200115 | -0.704488 |
| 39 | 8 | 0 | 4.299560 | -2.798299 | 2.898701 |
| 40 | 6 | 0 | 3.494167 | -3.506077 | 3.833920 |
| 41 | 8 | 0 | -5.689182 | -2.494240 | -0.435841 |
| 42 | 6 | 0 | -6.328695 | -3.148413 | -1.524610 |
| 43 | 1 | 0 | 5.562975 | -1.685836 | 1.058069 |
| 44 | 1 | 0 | 1.580517 | -2.605300 | 2.366780 |
| 45 | 1 | 0 | -0.038905 | 0.930866 | 0.511484 |
| 46 | 1 | 0 | 1.031538 | 1.530375 | -2.232918 |
| 47 | 1 | 0 | -5.659378 | 2.624961 | 1.201143 |
| 48 | 1 | 0 | -4.500896 | 3.942971 | 1.245941 |
| 49 | 1 | 0 | -4.267742 | 1.464750 | -0.576569 |
| 50 | 1 | 0 | -5.053711 | 2.973498 | -1.041555 |
| 51 | 1 | 0 | -2.922951 | 3.198053 | -1.978828 |
| 52 | 1 | 0 | -2.799681 | 4.164757 | -0.502391 |
| 53 | 1 | 0 | -1.559631 | 2.418035 | 0.561211 |
| 54 | 1 | 0 | -2.012639 | -0.648607 | 1.314246 |
| 55 | 1 | 0 | -4.369389 | -1.354224 | 1.384563 |
| 56 | 1 | 0 | -4.018599 | -2.881788 | -2.623972 |
| 57 | 1 | 0 | -1.706582 | -2.197523 | -2.704936 |
| 58 | 1 | 0 | 1.818170 | 1.407599 | 1.472904 |
| 59 | 1 | 0 | 2.927531 | 2.839132 | -2.455478 |
| 60 | 1 | 0 | 4.491933 | 4.427445 | -1.413282 |
| 61 | 1 | 0 | 4.721353 | 4.520566 | 1.056219 |
| 62 | 1 | 0 | 3.372546 | 3.001218 | 2.477715 |
| 63 | 1 | 0 | 3.622584 | -0.175650 | -2.526855 |
| 64 | 1 | 0 | 0.151421 | -0.744361 | -2.902472 |
| 65 | 1 | 0 | 1.670550 | -2.518055 | -2.872493 |
| 66 | 1 | 0 | -4.615146 | 0.129825 | 4.491923 |
| 67 | 1 | 0 | -3.156191 | -0.009832 | 3.491692 |
| 68 | 1 | 0 | -3.383969 | 1.406382 | 4.579018 |
| 69 | 1 | 0 | 6.311658 | 0.892652 | -1.470380 |
| 70 | 1 | 0 | 5.778743 | 0.775349 | 0.212044 |
| 71 | 1 | 0 | 6.720248 | -0.562161 | -0.553252 |
| 72 | 1 | 0 | 4.160282 | -3.947226 | 4.581568 |
| 73 | 1 | 0 | 2.810803 | -2.828198 | 4.355753 |
| 74 | 1 | 0 | 2.949070 | -4.323260 | 3.349802 |
| 75 | 1 | 0 | -7.363701 | -3.349588 | -1.231951 |
| 76 | 1 | 0 | -6.352706 | -2.506902 | -2.411774 |
| 77 | 1 | 0 | -5.852030 | -4.109795 | -1.743163 |
| C4： | | | | | |
| Center  Number | Atomic  Number | Atomic  type | Coordinates (Angstroms) | | |
|  |  |  | x | y | z |
| 1 | 6 | 0 | 4.491564 | -1.710156 | 0.783703 |
| 2 | 6 | 0 | 3.622826 | -2.439091 | 1.600255 |
| 3 | 6 | 0 | 2.253469 | -2.375409 | 1.365244 |
| 4 | 6 | 0 | 1.765607 | -1.607111 | 0.307510 |
| 5 | 6 | 0 | 2.626449 | -0.901873 | -0.541945 |
| 6 | 6 | 0 | 4.003123 | -0.928804 | -0.269753 |
| 7 | 8 | 0 | 0.403611 | -1.572635 | 0.173012 |
| 8 | 6 | 0 | -0.151120 | -0.762353 | -0.906924 |
| 9 | 6 | 0 | -0.023457 | 0.747428 | -0.505690 |
| 10 | 6 | 0 | 1.335948 | 1.213916 | -1.119303 |
| 11 | 6 | 0 | 1.981771 | -0.094439 | -1.656371 |
| 12 | 6 | 0 | -1.120795 | 1.597371 | -1.127354 |
| 13 | 6 | 0 | -3.925301 | 1.708202 | 2.073354 |
| 14 | 6 | 0 | -4.729248 | 2.570490 | 1.126654 |
| 15 | 6 | 0 | -4.279311 | 2.485810 | -0.331298 |
| 16 | 6 | 0 | -2.946973 | 3.183447 | -0.609094 |
| 17 | 7 | 0 | -1.823366 | 2.359530 | -0.224690 |
| 18 | 6 | 0 | -1.595912 | -1.245494 | -1.015457 |
| 19 | 6 | 0 | -2.156458 | -1.703709 | -2.214208 |
| 20 | 6 | 0 | -3.494172 | -2.111418 | -2.271057 |
| 21 | 6 | 0 | -4.302973 | -2.080923 | -1.135549 |
| 22 | 6 | 0 | -3.758085 | -1.644677 | 0.068374 |
| 23 | 6 | 0 | -2.421560 | -1.231577 | 0.125618 |
| 24 | 6 | 0 | 3.034514 | 3.054549 | -0.869242 |
| 25 | 6 | 0 | 2.231073 | 2.082987 | -0.246460 |
| 26 | 6 | 0 | 2.325452 | 1.943144 | 1.145734 |
| 27 | 6 | 0 | 3.183576 | 2.757035 | 1.890509 |
| 28 | 6 | 0 | 3.962919 | 3.720843 | 1.256794 |
| 29 | 6 | 0 | 3.889939 | 3.868550 | -0.124568 |
| 30 | 8 | 0 | 2.830256 | 0.148546 | -2.777711 |
| 31 | 8 | 0 | -1.336670 | 1.612882 | -2.335787 |
| 32 | 6 | 0 | 0.735569 | -0.850121 | -2.166566 |
| 33 | 8 | 0 | 1.042118 | -2.171764 | -2.621179 |
| 34 | 8 | 0 | -2.717381 | 1.809802 | 2.252972 |
| 35 | 8 | 0 | -4.738981 | 0.833975 | 2.727692 |
| 36 | 6 | 0 | -4.078648 | 0.045311 | 3.719150 |
| 37 | 8 | 0 | 4.813202 | -0.184395 | -1.090049 |
| 38 | 6 | 0 | 5.989008 | 0.339571 | -0.475474 |
| 39 | 8 | 0 | 4.235819 | -3.155707 | 2.591759 |
| 40 | 6 | 0 | 3.405371 | -4.006231 | 3.373418 |
| 41 | 8 | 0 | -5.589468 | -2.499825 | -1.332709 |
| 42 | 6 | 0 | -6.453960 | -2.470939 | -0.204666 |
| 43 | 1 | 0 | 5.556466 | -1.780299 | 0.983503 |
| 44 | 1 | 0 | 1.538013 | -2.909117 | 1.981169 |
| 45 | 1 | 0 | -0.057225 | 0.845296 | 0.583251 |
| 46 | 1 | 0 | 1.117460 | 1.839141 | -1.999154 |
| 47 | 1 | 0 | -5.779737 | 2.257764 | 1.178266 |
| 48 | 1 | 0 | -4.695897 | 3.601290 | 1.496805 |
| 49 | 1 | 0 | -4.239175 | 1.439680 | -0.655946 |
| 50 | 1 | 0 | -5.047361 | 2.981169 | -0.938306 |
| 51 | 1 | 0 | -2.874311 | 3.414160 | -1.677352 |
| 52 | 1 | 0 | -2.871530 | 4.126490 | -0.058051 |
| 53 | 1 | 0 | -1.630711 | 2.268668 | 0.782514 |
| 54 | 1 | 0 | -1.570880 | -1.752194 | -3.129029 |
| 55 | 1 | 0 | -3.906005 | -2.456736 | -3.216346 |
| 56 | 1 | 0 | -4.341699 | -1.614130 | 0.981042 |
| 57 | 1 | 0 | -2.018172 | -0.898897 | 1.080436 |
| 58 | 1 | 0 | 2.999472 | 3.183394 | -1.950323 |
| 59 | 1 | 0 | 1.755845 | 1.187741 | 1.680374 |
| 60 | 1 | 0 | 3.245932 | 2.632239 | 2.968685 |
| 61 | 1 | 0 | 4.627721 | 4.353379 | 1.838686 |
| 62 | 1 | 0 | 4.497873 | 4.617240 | -0.625661 |
| 63 | 1 | 0 | 3.740687 | 0.209313 | -2.427008 |
| 64 | 1 | 0 | 0.296319 | -0.324911 | -3.023855 |
| 65 | 1 | 0 | 1.827331 | -2.072426 | -3.192511 |
| 66 | 1 | 0 | -4.816418 | -0.630273 | 4.160638 |
| 67 | 1 | 0 | -3.283707 | -0.557000 | 3.269894 |
| 68 | 1 | 0 | -3.677690 | 0.686686 | 4.510127 |
| 69 | 1 | 0 | 6.372197 | 1.137088 | -1.119186 |
| 70 | 1 | 0 | 5.775878 | 0.776265 | 0.506344 |
| 71 | 1 | 0 | 6.761972 | -0.431817 | -0.404197 |
| 72 | 1 | 0 | 4.047856 | -4.547782 | 4.074373 |
| 73 | 1 | 0 | 2.688947 | -3.423062 | 3.961210 |
| 74 | 1 | 0 | 2.895404 | -4.747000 | 2.748424 |
| 75 | 1 | 0 | -7.438483 | -2.821914 | -0.528683 |
| 76 | 1 | 0 | -6.101123 | -3.148025 | 0.580372 |
| 77 | 1 | 0 | -6.572952 | -1.451113 | 0.176316 |
| C5： | | | | | |
| Center  Number | Atomic  Number | Atomic  type | Coordinates (Angstroms) | | |
|  |  |  | x | y | z |
| 1 | 6 | 0 | 4.483445 | -1.534473 | 0.957042 |
| 2 | 6 | 0 | 3.661472 | -2.051352 | 1.959096 |
| 3 | 6 | 0 | 2.279090 | -2.023247 | 1.787513 |
| 4 | 6 | 0 | 1.723749 | -1.504889 | 0.619886 |
| 5 | 6 | 0 | 2.533745 | -1.007925 | -0.406150 |
| 6 | 6 | 0 | 3.923872 | -0.991808 | -0.209106 |
| 7 | 8 | 0 | 0.357494 | -1.495867 | 0.554474 |
| 8 | 6 | 0 | -0.260197 | -0.934089 | -0.642016 |
| 9 | 6 | 0 | -0.121632 | 0.625736 | -0.580355 |
| 10 | 6 | 0 | 1.200616 | 0.943846 | -1.350263 |
| 11 | 6 | 0 | 1.825788 | -0.452161 | -1.630268 |
| 12 | 6 | 0 | -1.256160 | 1.329415 | -1.309322 |
| 13 | 6 | 0 | -3.832718 | 2.175434 | 1.971285 |
| 14 | 6 | 0 | -4.698197 | 2.832191 | 0.919853 |
| 15 | 6 | 0 | -4.363443 | 2.416256 | -0.512239 |
| 16 | 6 | 0 | -3.055212 | 3.008143 | -1.039234 |
| 17 | 7 | 0 | -1.905776 | 2.277391 | -0.555814 |
| 18 | 6 | 0 | -1.705123 | -1.422696 | -0.568253 |
| 19 | 6 | 0 | -2.472974 | -1.149738 | 0.578839 |
| 20 | 6 | 0 | -3.805003 | -1.561469 | 0.672472 |
| 21 | 6 | 0 | -4.407268 | -2.258183 | -0.373110 |
| 22 | 6 | 0 | -3.658309 | -2.547516 | -1.511237 |
| 23 | 6 | 0 | -2.320785 | -2.137197 | -1.605103 |
| 24 | 6 | 0 | 2.301070 | 2.135233 | 0.652192 |
| 25 | 6 | 0 | 2.133907 | 1.975325 | -0.731100 |
| 26 | 6 | 0 | 2.892803 | 2.792427 | -1.587518 |
| 27 | 6 | 0 | 3.774410 | 3.748088 | -1.078954 |
| 28 | 6 | 0 | 3.918708 | 3.898970 | 0.296541 |
| 29 | 6 | 0 | 3.185077 | 3.090988 | 1.160744 |
| 30 | 8 | 0 | 2.611356 | -0.457259 | -2.821937 |
| 31 | 8 | 0 | -1.540978 | 1.083304 | -2.477976 |
| 32 | 6 | 0 | 0.558346 | -1.291817 | -1.900480 |
| 33 | 8 | 0 | 0.847950 | -2.681017 | -2.084057 |
| 34 | 8 | 0 | -2.613298 | 2.282383 | 2.033658 |
| 35 | 8 | 0 | -4.601733 | 1.487188 | 2.858356 |
| 36 | 6 | 0 | -3.873892 | 0.889020 | 3.932066 |
| 37 | 8 | 0 | 4.690868 | -0.457084 | -1.215123 |
| 38 | 6 | 0 | 5.822619 | 0.291107 | -0.771488 |
| 39 | 8 | 0 | 4.079233 | -2.595207 | 3.142706 |
| 40 | 6 | 0 | 5.481570 | -2.728511 | 3.335139 |
| 41 | 8 | 0 | -5.715366 | -2.595148 | -0.159631 |
| 42 | 6 | 0 | -6.361951 | -3.328883 | -1.191959 |
| 43 | 1 | 0 | 5.560204 | -1.557284 | 1.067384 |
| 44 | 1 | 0 | 1.628314 | -2.415231 | 2.565029 |
| 45 | 1 | 0 | -0.098194 | 0.953781 | 0.462995 |
| 46 | 1 | 0 | 0.931362 | 1.368166 | -2.330247 |
| 47 | 1 | 0 | -5.745004 | 2.568021 | 1.116291 |
| 48 | 1 | 0 | -4.624630 | 3.917717 | 1.049769 |
| 49 | 1 | 0 | -4.350583 | 1.323512 | -0.596936 |
| 50 | 1 | 0 | -5.175138 | 2.778938 | -1.155134 |
| 51 | 1 | 0 | -3.061881 | 2.984477 | -2.134171 |
| 52 | 1 | 0 | -2.940170 | 4.051550 | -0.728236 |
| 53 | 1 | 0 | -1.653916 | 2.402492 | 0.434934 |
| 54 | 1 | 0 | -2.029831 | -0.614954 | 1.416600 |
| 55 | 1 | 0 | -4.376827 | -1.339519 | 1.569284 |
| 56 | 1 | 0 | -4.077784 | -3.091854 | -2.350549 |
| 57 | 1 | 0 | -1.775289 | -2.390361 | -2.511346 |
| 58 | 1 | 0 | 1.766776 | 1.511810 | 1.364544 |
| 59 | 1 | 0 | 2.799515 | 2.689141 | -2.667972 |
| 60 | 1 | 0 | 4.345088 | 4.374947 | -1.758993 |
| 61 | 1 | 0 | 4.601900 | 4.643972 | 0.694986 |
| 62 | 1 | 0 | 3.300754 | 3.200631 | 2.236192 |
| 63 | 1 | 0 | 3.540467 | -0.342395 | -2.541877 |
| 64 | 1 | 0 | 0.069972 | -0.957086 | -2.824288 |
| 65 | 1 | 0 | 1.606988 | -2.707981 | -2.697023 |
| 66 | 1 | 0 | -4.584336 | 0.346622 | 4.561854 |
| 67 | 1 | 0 | -3.135681 | 0.177270 | 3.551333 |
| 68 | 1 | 0 | -3.389813 | 1.660234 | 4.539150 |
| 69 | 1 | 0 | 6.130002 | 0.946140 | -1.592245 |
| 70 | 1 | 0 | 5.585424 | 0.923012 | 0.091267 |
| 71 | 1 | 0 | 6.658602 | -0.378475 | -0.547041 |
| 72 | 1 | 0 | 5.641716 | -3.211636 | 4.303874 |
| 73 | 1 | 0 | 5.927857 | -3.367847 | 2.566140 |
| 74 | 1 | 0 | 5.968957 | -1.748581 | 3.367277 |
| 75 | 1 | 0 | -7.388409 | -3.526596 | -0.868475 |
| 76 | 1 | 0 | -6.411525 | -2.747028 | -2.118262 |
| 77 | 1 | 0 | -5.873201 | -4.295017 | -1.356232 |
